# Predicting the Trauma and Injury Severity Score (TRISS) in patients admitted to trauma centers after trauma: A modeling study based on data from the French National Programme for Medicalization of Information

**DOI:** 10.1371/journal.pone.0354700

**Published:** 2026-08-17

**Authors:** Arthur James, David Hajage, Noémie Simon-Tillaux, Sophie Tezenas du Montcel, Pierre Rufat, Bruno Riou, Mathieu Raux

**Affiliations:** 1 Sorbonne University, GRC 29, and Department of Anesthesiology and Critical Care Medicine, Pitié-Salpêtrière Hospital, Assistance Publique–Hôpitaux de Paris (AP-HP), Paris, France; 2 Sorbonne University, Pierre Louis Institute of Epidemiology and Public Health (IPLESP), INSERM UMR-S, Department of Public Health, Pharmacoepidemiology Center (Cephepi), and Clinical Research Unit, Pitié-Salpêtrière Hospital, AP-HP, Paris, France; 3 Department of Biostatistics and Epidemiology, Gustave Roussy Institute, Oncostat INSERM U1018, Paris-Saclay University, Villejuif, France; 4 Sorbonne University, Paris Brain Institute, INSERM–CNRS UMR, Department of Medical Information, Pitié-Salpêtrière Hospital, AP-HP, Inria Paris, France; 5 Department of Medical Information, Pitié-Salpêtrière Hospital, AP-HP, Paris, France; 6 Sorbonne University, INSERM UMR-S, IHU ICAN, and Emergency Department, Pitié-Salpêtrière Hospital, AP-HP, Paris, France; 7 Sorbonne University, INSERM UMR-S, and Department of Anesthesiology and Critical Care Medicine, Pitié-Salpêtrière Hospital, AP-HP, Paris, France; PGIMER: Post Graduate Institute of Medical Education and Research, INDIA

## Abstract

**Background:**

Reliable mortality prediction is central to trauma research, quality assessment, and resource allocation. While the French national hospital discharge database (PMSI) provides comprehensive nationwide data, it does not capture clinical parameters required for established severity scores such as TRISS, which is used for predicting in-hospital survival after trauma and necessary for implementing national-level evaluations of health system performance. This study tested whether TRISS could nonetheless be accurately derived from PMSI data.

**Methods:**

We conducted a retrospective study of adult patients admitted for severe trauma to an academic trauma center between 2012 and 2023. Clinical data from medical records were linked with corresponding PMSI records. Surrogate predictors of TRISS were derived from PMSI codes. An extreme gradient boosting (XGBoost) model was used, model performance was assessed using bootstrap validation and interpretability was explored through SHapley Additive exPlanations analyses.

**Results:**

A total of 2,523 trauma patients were successfully matched between PMSI and medical records. Median age was 35 years, 79% were male, and in-hospital mortality was 15%. Correlation between Simplified Acute Physiology Score II (SAPS II) in PMSI and medical records was strong (ρ = 0.88), while concordance for shock and coma was weak (κ ≈ 0.25). The XGBoost model using individual PMSI codes achieved the best performance (optimism-adjusted R^2^ = 0.72). SAPS II was the main contributor, with additional contributions from shock, coma, and anatomical injury codes.

**Conclusion:**

TRISS can be reliably approximated from PMSI data using an XGBoost model. This approach enables patient-specific assessment of trauma severity and mortality risk from PMSI, opening perspectives for nationwide surveillance and quality evaluation of trauma care in France.

## Introduction

Trauma—resulting from road traffic and domestic accidents, assaults, or self-harm injuries—affects 144,000 patients annually in France [1]. The societal impact as severe trauma predominantly affects a young population, leading to a substantial number of years of life lost [2]. These patients also frequently experience long-term functional impairments, further contributing to the overall burden of trauma. For these reasons, severe trauma is increasingly recognized as the gateway to a chronic disease process [3]. Despite the existence of a national trauma registry in France, there is still no comprehensive national overview of trauma care—yet such a perspective is essential to guide improvements in care quality and to evaluate the effectiveness of healthcare delivery.

The French national hospital discharge database (Programme de Médicalisation des Systèmes d’Information, PMSI) has been used since the 1980s to provide a standardized and comprehensive description of the medical activities of healthcare facilities in France. It includes information such as medico-administrative data, a principal diagnosis, other associated diagnoses and all procedures performed. As part of the National Health Data System (SNDS), the PMSI is exhaustively completed for all patients requiring hospital care as it underpins hospital reimbursement mechanisms. These features make this tool an ideal tool to address the needs of stakeholders involved in trauma care organization.

However, the use of the PMSI has, to date, been limited—primarily due to its inability to accurately capture the clinical severity of trauma patients [4]. Indeed, the PMSI does not directly record key prognostic clinical variables such as arterial blood pressure, level of consciousness, or the nature of the trauma (e.g., penetrating vs blunt injuries). Nevertheless, these variables are critical for calculating standardized trauma severity scores that are used to categorize severe trauma patients in research. Among these scores, the Trauma and Injury Severity Score (TRISS) is one of the most validated and widely used, as it accurately estimates the probability of death for each patient based on a combination of clinical features (such as age, arterial blood pressure, and mechanism) and anatomical injuries assessed by the Abbreviated Injury Scale (AIS) [5]. Originally developed to identify potentially preventable deaths within trauma registries, TRISS has since been primarily employed at a population level to assess injury severity based on predicted mortality. Therefore, TRISS is widely used for epidemiological research purposes or even to improve research methodology in trauma [6,7].

We hypothesized that an approximation of the TRISS, based on surrogate variables available in the PMSI, can be predicted using administrative data. The primary objective of this study was to develop a model to estimate a TRISS-like severity score in severe trauma patients admitted to trauma centers using surrogate data available in the PMSI.

## Methods

This study complies with the Transparent Reporting of a Multivariable Prediction Model for Individual Prognosis or Diagnosis (TRIPOD) Statement [8]. The data used in this study originate exclusively from routine care, with no data collected specifically for research purposes. In accordance with French regulations, all patients are informed that their care data may be used for research and were given the opportunity to object. Therefore, no supplementary authorization was required for this study.

For the purpose of linking TRISS and PMSI, the respective two data sources were combined, with the medical records on one hand and the PMSI records on the other. Data were collected from patients’ medical records and extracted on June 30, 2023. The collected information included the injuries they present using the abbreviated injury score (AIS) [9], the immediate impact of these injuries on their health, and the resources required for their immediate hospital care, such as transfusion or surgery [10]. The intrahospital outcomes of these patients were also recorded, including in-hospital mortality. Candidate trauma patients were identified using the medical record.

The PMSI data of the trauma patients were extracted by the Medical Information Department of the hospital on the October 25, 2022. Within the PMSI, patients are classified into Homogeneous Groups of Patients (GHM) using the International Classification of Diseases (ICD, 10th revision) codes [11], and using a specific group for severe trauma patients (GHM-26) [12]. However, it is known that this coding poorly identifies severe trauma patients [4]. Therefore, in accordance with the method proposed by Bège et al., a modified list of codes was developed from the GHM-26 list to optimize the selection of severe trauma patients from the PMSI [1]. For this purpose, four trauma experts independently recategorized each GHM-26 code as “Non-severe,” “Intermediate,” or “Severe.” The presence of two intermediate codes for the same patient resulted in classifying the trauma as “Severe.” Any patient with at least one “Severe” code was categorized as having sustained severe trauma. The detailed process is described in Supplementary Material 1 in S1 File.

This study included all trauma patients aged over 15 years between January 31, 2012, and December 31, 2021, admitted to the trauma unit of an academic level-1 trauma center (Pitié-Salpêtrière Hospital, Assistance Publique des Hôpitaux de Paris, Paris, France). These patients were admitted to the trauma unit after being identified by pre-hospital medical regulation based on their medical history and clinical examination as being at risk of severe trauma and therefore all received care consistent with the management of severe trauma patients [13–15]. This process prioritizes the admission of patients whose vital or functional prognosis is or may be compromised but may also include patients who ultimately do not present signs of severe injury, reflecting cases of undertriage. Patients with missing data on admission date, birth date, or sex, as well as patients with burn injuries, were excluded.

During data collection, information from the medical records was de-identified and remained strictly anonymous. Therefore, for all patients admitted to the trauma unit, a matching process was performed between the medical record and the PMSI. This matching was done based on the date of birth, hospital admission date, and sex. This was sufficiently discriminative to prevent duplicates.

The primary outcome measure was the predictive performance of the models estimating the TRISS, derived from medical records, using surrogate variables available in the PMSI [5]. This variable is calculated for each patient based on the medical record using the initial arterial blood pressure, Glasgow coma score, respiratory rate, age, type of injury (penetrating or not), and the Injury Severity Score (which reflects all identified traumatic injuries based on the AIS of each anatomical lesion) [9]. The TRISS is expressed as a value between 0 (lowest predicted risk of death) and 1 (highest predicted risk of death) [5].

The TRISS combines anatomical and physiological variables, with the latter reflecting the functional consequences of injuries. Therefore, any attempt to replicate TRISS’s predictive value for mortality must account for the impact of trauma on vital functions. In this regard, the PMSI contains only one severity score: the SAPS II score [16]. This score is well-validated for predicting mortality in critically ill patients and includes information such as mode of admission, age, the presence of chronic conditions, as well as vital signs and laboratory values at admission. However, it does not account for certain trauma-specific features. As result, surrogate predictors of TRISS had to be identified within the PMSI. To achieve this, variables were selected based on both clinical expertise and the observed correlation between candidate PMSI variables and corresponding clinical data from the medical record. Specifically, codes related to shock were used as proxies for arterial blood pressure, codes related to coma as proxies for the Glasgow coma score, and codes related to injuries assessment as proxies for the AIS components of the ISS.

In the medical record, patients were considered as being in shock if the systolic arterial blood pressure was below 90 mmHg or the diastolic blood pressure was below 60 mmHg upon admission, or if vasopressors were used. In comparison, the PMSI does not provide a specific definition of a shock, and shock diagnostic codes (R57, R570, R571, R572, R578, R579, T794) were retrieved for the first hospitalization unit, along with the procedure codes related to the administration of catecholamines (EQLF003, EQLF001) or significant vascular filling (EQLF002). Using these codes, a derived unique variable was created. A unique PMSI shock variable was thus created.

In the medical record, patients were considered as being in coma if the Glasgow coma score was strictly less than 9 upon admission, or if the patient was sedated with rapid-acting hypnotic agent (i.e., propofol, etomidate, or ketamine) in combination with opioids before hospital admission. The PMSI does not provide a specific definition of coma and codes related to a coma were retrieved (R40, R400, R401, R402, R4028, R4018). A unique PMSI coma variable was thus created.

In the medical record, patients’ injuries were coded using the AIS. In the PMSI, the codes from the D-130 modified list of GHM-26 were categorized to fit with the six anatomical topographic regions of the AIS (face, head and neck, chest, abdominal and pelvic, extremities, external)(Supplementary Material 2 in S1 File). Six trauma region variables were thus created.

Finally, in the medical record, patients were caterorized according to the mechanism of injury (Blunt or penetrating). In the PMSI, codes related to penetrating injuries were retrieved from the D-130 list (S0601, S0611, S0621, S0631, S0641, S0651, S0661, S0681, S0691, S2681, S2691, S2701, S2711, S2721, S2731, S2741, S2761, S3601, S3621, S3631, S3641, S3651, S3661, S3681, S3701, S3711, S3721, S3731, S3741, S3751, S3761, S3771, S37810, S37818, S054, S055, S056). A unique PMSI mechanism variable was thus created. All ICD-10 codes are compiled in Supplementary Material 3 in S1 File.

## Statistical analysis

Continuous variables are reported as median and interquartile range (IQR) and categorical variables are reported as numbers and relative percentages. Effect estimates are reported with 95% confidence intervals.

This is a retrospective and monocentric study, and no sample size was computed a priori. According to Riley et al. [17], with a number of predictor variables of 10, an anticipated proportion of variance explained (R-squared) of 50%, an average TRISS value of 0.12 with a standard error of 0.24, the minimum sample size required is 245 subjects overall [18]. With a number of predictor variables of 45, the minimum sample size required is 554 overall.

As the TRISS is a probability between 0 and 1, a logit transformation of its value was first performed and used as the dependent variable in all models. Three types of models were a priori chosen to predict the logit of the TRISS: linear regression, ridge regression and extreme-gradient boosting regression.

The whole database of observations linked from the medical record and the PMSI was used to train the models and evaluate their performance. Indeed, using all available data to estimate model parameters, rather than partitioning the dataset, may improve the generalizability of prediction models. [19] Nonetheless, to correct the apparent performance of the model from the optimism due to using only one cohort of patients, bootstrap validation was performed (n = 200 iterations). For Ridge regression models, the penalty term (lambda) was selected using a separate bootstrap procedure (n = 200 iterations) to minimize the mean squared prediction error; this procedure was distinct from the bootstrap used to estimate the optimism-adjusted R^2^, ensuring that parameter selection and performance correction were independent.

The contribution of the different variables in the proposed models was described using a SHAP (SHapley Additive exPlanations) diagram for the extreme gradient boosting regression model and the beta coefficients associated with each parameter of the regressions for the linear regression and Ridge regression models. Models’ performances were evaluated using: 1) the apparent and adjusted R^2^ coefficient: the “apparent” R^2^ was calculated on the full dataset without correction, whereas the “optimism-adjusted” R^2^ was estimated using bootstrap validation (n = 200 iterations) to correct for potential overfitting of the prediction models; 2) the apparent and optimism-adjusted root mean squared error (RMSE); 3) the SHAP (SHapley Additive exPlanations) diagram for the extreme gradient boosting models; and 4) the associated beta regression coefficients for the various variables in the linear regression and ridge regression models.

The correlation between the SAPS II on one side and AIS on the other side was calculated using a Spearman correlation coefficient and the 95% confidence interval of the Spearman correlation coefficient was estimated using bootstrap (9999 iterations). The concordance between the coma and the shock variable in the hospital data and that in the PMSI was studied using Cohen’s Kappa coefficient. Correlation and concordance values were considered very weak when their absolute value was between 0 and 0.19, weak between 0.2 and 0.39, moderate between 0.40 and 0.59, strong between 0.6 and 0.79, and very strong between 0.8 and 1 [20].

A sensitivity analysis was conducted using a second and larger set of predictors and using Ridge regression, extreme-gradien boosting regression only. This analysis was based on individual codes that were used to derive all the previous predictors mentioned (shock, coma, penetrating lesion, and codes used to categorize the number of lesions according to anatomical topography), as well as SAPS II and age. In this analysis, all the predictors with a near zero variance were removed to avoid introducing uninformative variables. A set of 45 codes that may predict the TRISS were thus selected (S017, S021, S022, S023, S024, S061, S062, S063, S064, S065, S066, S067, S069, S122, S220, S222, S223, S224, S225, S269, S270, S271, S272, S273, S320, S321, S323, S324, S325, S327, S360, S361, S368, S370, S420, S421, S729, S822, T792, T794, T796, T797, R571, R578, R402).

Missing data imputation was not performed and only complete cases were analyzed, except for the SAPS II variable in the PMSI, due to the importance of this variable in prediction. Thus, in cases of missing data, the SAPS II variable was imputed using k-nearest neighbors for models based on aggregated variables, and by the median for models based on individual codes due to high computation times.

All tests were performed at a type-one error rate of 5% (two-tailed formulation) without any adjustment for multiplicity. Wilcoxon rank sum test or Pearson’s Chi-squared test were used as appropriate. All analyses were performed using R version 4.3.0. The following key packages were used: caret (version 6.0–94), xgboost (version 1.7.5.1), and SHAPforxgboost (version 0.1.3).

## Results

**Fig 1:**
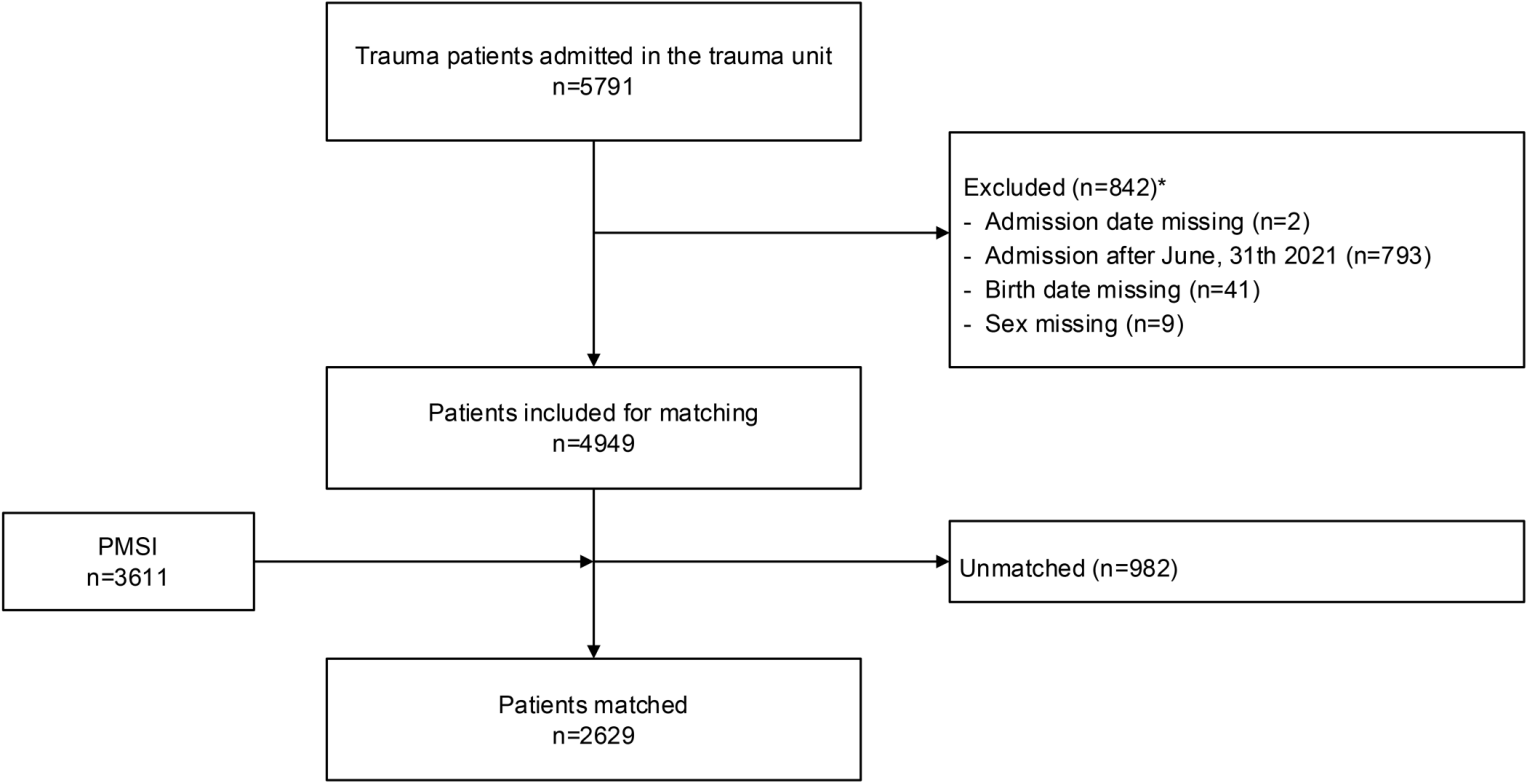
Study flowchart. *: Some patients can have several reasons for exclusion. PMSI, French national hospital discharge database (Programme de médicalisation des systèmes d’information).

From the 5,791 patients identified from the medical records during the study period, 842 were excluded due to missing key variables for the linkage, leading to the inclusion of 4,949 unique patients. During the same period, 3,611 severe trauma patients were identified in the local PMSI based on pre-specified inclusion criteria. Among these two populations, 2,629 unique patients were matched, of which 2,523 patients had an available TRISS score (Fig 1)

Among patients matched, the median age was 35 years [IQR, 25−51], with a male predominance (n = 2079, 79%). The median ISS score was 19 [IQR 11−29], with an in-hospital mortality rate of 15% (n = 383). The median TRISS score was 0.03 [IQR 0.01–0.18], while the mean TRISS score corresponded to a predicted mortality of 18%, compared with an observed mortality of 15% (n = 383). The main characteristics of the patients are presented in Table 1. Supplementary Material 2 in S1 File presents a comparison between matched and unmatched patients, with patients being significantly more severely traumatized in the matched group compared to the unmatched group.

**Table 1:** Baseline characteristics from the matched population according to the medical record.

Variables	N (MV)	Matched population
**General informations**		
Age (years)	2629 (0)	35 [25; 51]
Sex		
Female	2629 (0)	550 (21)
Male		2079 (79)
Mechanism of injury		
Fall from height	2629 (0)	696 (27)
Penetrating		488 (19)
Public road accident		1310 (50)
Other and unknown		135 (5)
Nightime Working hours	2629 (0)	1872 (71)
**Baseline severity**		
First GCS	2612 (17)	15 [9; 15]
Coma	2628 (1)	986 (38)
Cardiac arrest before hospital admission	2628 (1)	172 (7)
First Systolic blood pressure (mmHg)	1957 (672)	123 [105; 140]
Hemorrhagic shock within the first 6 hours	2629 (0)	389 (16)
Traumatic Brain Injury (according to CT scan)	2621 (8)	1031 (39)
Lactate (mmol/l) at hospital arrival	2385 (244)	2 [1; 3]
**Ressources use and outcomes**		
Mechanical ventilation at hospital admission	2617 (12)	1106 (42)
Surgery or interventional radiology procedure within the first 24 hours		
before CT scan	2408 (221)	162 (7)
during hospital stay	1031 (1598)	1503 (94)
Transfusion in resuscitation room		
RBC units	1809 (820)	0 [0; 1]
Platelet		0 [0; 0]
FPP		0 [0; 0]
In-hospital death	2629 (0)	383 (14.6)
Number of ventilator free days (days, at day 28)	2292 (337)	26 [6; 28]
Acute respiratory distress syndrome	2386 (243)	252 (11)
Renal replacement therapy	1500 (1129)	36 (3)
Length of hospital stay (days)	2629 (0)	11 [4; 30]
**Injury descriptions**		
Abbreviated Injury Scale		
Head and neck	2625 (4)	2 [0; 3]
Face		0 [0; 1]
Chest		2 [0; 3]
Abdomen, pelvic contents		0 [0; 2]
Extremity, pelvic girdle		2 [0; 3]
External	0 [0; 0]
**Scores**		
Injury Severity Score	2625 (4)	19 [11; 29]
Trauma Injury Severity Score	2523 (106)	0.03 [0.01; 0.18]
Simplified Acute Physiology Score 2	2621 (8)	30 [18; 48]

Continuous variables are reported as median and IQR and categorial variables are reported as numbers and relative percentages. CT, Comptuted tomography; FFP, Fresh Frozen Plasma; GCS, Glasgow Coma Score; IQR, Inter Quartile Range; MV, Missing Values; RBC, Red Blood Cell; SOFA, Sequential Organ Failure Assessment. The mean (Standard deviation) Trauma Injury Severity Score is 0.18 (0.29).

In the PMSI, 28% of patients (n = 730) presented with shock and 12% of patients (n = 319) with coma. The proportion of patients with penetrating trauma was 3% (n = 70). The reported median SAPS II score was 32 [IQR 18–52]. These data are reported in Table 2 and in full in Supplementary Materials 4 and 5 in S1 File.

**Table 2:** Relevant codes according to the French national hospital discharge database (Programme de médicalisation des systèmes d’information, PMSI).

Label	N (Missing values)	Value
*Shock requiring fluid resuscitation or catecholamines (admission)*	None	
Yes		1899 (72)
No		730 (28)
*Coma*	None	
*Yes*		319 (12)
*No*		2310 (88)
*SAPS II (admission)*	2594 (35)	30 [17; 48]
*Penetrating trauma*	None	
Yes		70 (3)
No		2559 (97)
*Shock requiring fluid resuscitation or catecholamines during hospitalization*		
Yes	None	868 (33)
No		1761 (67)

Continuous variables are reported as median and IQR and categorial variables are reported as numbers and relative percentages. A total of 29 variables were imputed for the SAPS II. *In the PMSI, it is not possible to distinguish a ‘no’ from missing data; one can only observe that the code is absent. MV, Missing Values. SAPS II, Simplified Acute Physiology Score 2

The comparison of variables available both in the medical record and the PMSI indicated a Spearman’s correlation of 0.88 [95% CI 0.87–0.90] for SAPS II, as well as Kappa coefficients of 0.28 [95% CI 0.25–0.32] and 0.24 [95% CI 0.21–0.27] for the occurrence of shock and coma, respectively (Supplementary Material 6 in S1 File). Similarly, strong correlations were established between the coded AIS in the medical record and the number of codes related to the affected areas reported in the PMSI (Supplementary Material 7 in S1 File).

Several models were tested to predict the TRISS from the PMSI of the 2,523 matched patients for whom a TRISS had been calculated from the medical records. The predictive performance of these models are reported in Table 3. The extreme gradient boosting model based on individual codes was the most performant, with an apparent R^2^ of 0.84. This value dropped to 0.72 after adjustment for optimism. Similarly, the extreme gradient boosting model based on aggregated codes reported an apparent R^2^ of 0.82 and an optimism-adjusted R^2^ of 0.70.

**Table 3:** Predictive models performances.

	Predictive model with aggregated covariates for hospital coding			Predictive models with individual codes as covariates	
	**Linear regression**	**Coefficients from the ridge regression**	**Extreme gradient boosting regression**	**Coefficients from the ridge regression**	**Extreme gradient boosting regression**
Penality term lambda	–	0.18	–	0.19	–
Apparent R squared	0.63	0.63	0.82	0.64	0.84
Optimism-adjusted R squared	0.63	0.63	0.70	0.63	0.72
Apparent RMSE	1.52	1.52	1.07	1.50	0.99
Optimism-adjusted RMSE	1.52	1.52	1.41	1.53	1.39

The list of individual codes corresponds to the codes from the GHM26 table D-130, as well as synonymous codes for coma, shock, and penetrating injuries taken individually (rather than aggregated into a presence/absence category or a cumulative sum of injuries in a given area). Some non-informative codes (those not present in any patient) or very infrequent codes (those present in very few patients) were excluded from the regressions. RMSE: root mean squared error.

**Fig 2:**
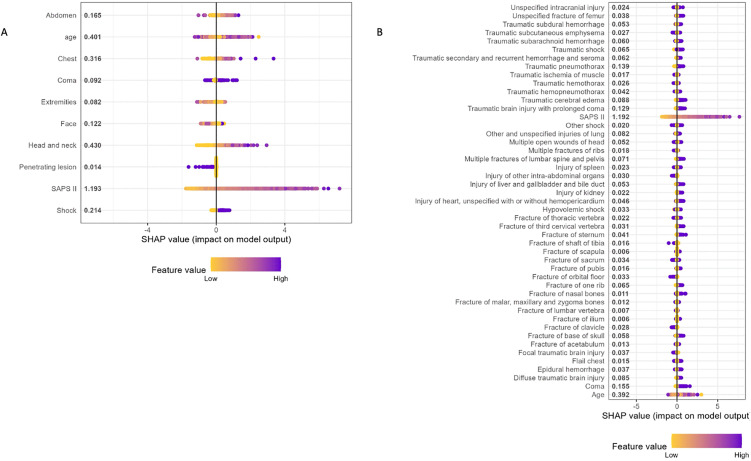
SHAP (SHapley Additive exPlanations) values to visualize the influence of each input variable on the predicted TRISS value. **A: SHAP for the model using aggregated codes as covariates. B: SHAP for the model using individual codes as covariates.** SAPSII, Simplified Acute Physiology Score 2. A SHAP summary plot illustrates the contribution of each variable to the model’s prediction of mortality. Each dot represents a patient, with the position on the x-axis indicating the variable’s impact on the predicted probability of death (positive values increase predicted mortality; negative values reduce it). The color reflects the actual value of the variable for that patient (e.g., purple for higher values, orange for lower ones). In this graph, it can thus be understood that the higher the SAPS II value (in purple), the more it is associated with a higher probability of death. Conversely, it can be observed that the presence of a penetrating lesion is associated with a reduced probability of death.

The SHAP diagrams indicate that the SAPS II is the variable contributing most to the model with a positive association (Fig 2A and 2B). The coefficients of the different models and the percentage of variability explained by each covariate in the extreme gradient boosting model are reported in Supplementary Material 8 and 9 in S1 File.

**Fig 3:**
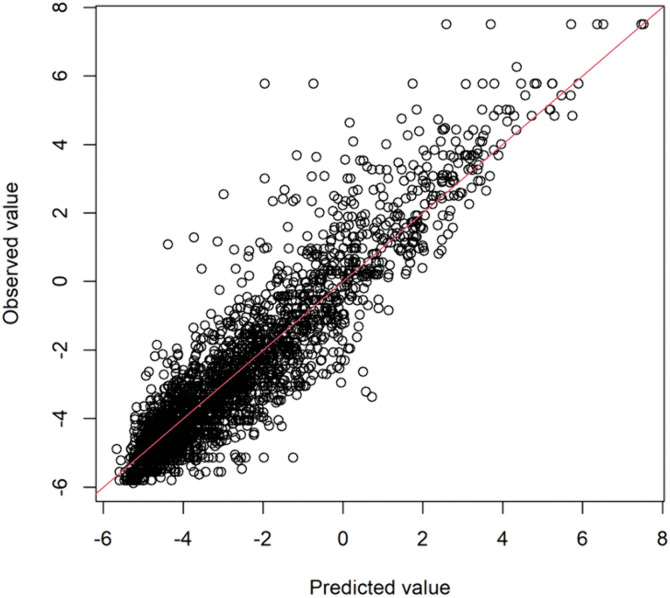
Concordance between observed and predicted values (logit(TRISS)) – Extreme gradient boosting with individual codes. The observed values of the Trauma and Injury Severity Score (TRISS) are those calculated based on the covariates collected directly in the medical record (n = 2.523). The predicted values of the TRISS are those estimated with the extreme gradient boosting with individual codes. Observed and predicted TRISS values demonstrated a strong linear relationship, suggesting good calibration of the extreme gradient boosting model across the range of predicted scores, with larger prediction errors observed at the extremes of the TRISS distribution.

“The agreement between the TRISS values predicted by the extreme gradient boosting model based on individual codes and those calculated from the medical record is presented in Fig 3**.**

## Discussion

This study demonstrates that a TRISS-like severity score, derived from surrogate PMSI variables, can be accurately estimated in patients admitted to a level-1 trauma center. Although this model is not intended to be integrated into trauma care workflows, it opens the way for national longitudinal studies using PMSI data, with a precise characterization of estimated injury severity at hospital admission. In fact, the extreme gradient boosting method, with an apparent R^2^ of 0.84 for predicting TRISS, highlights the potential of machine learning in leveraging administrative health data. This opens the door to severity prediction for severe trauma patients directly within a nationwide database. However, this prediction appears to be strongly influenced by the SAPS II score and, to a lesser extent, by age. Variables with a weaker correlation such as shock or coma had less impact on the prediction. This suggests that while the model is effective, its reliance on these variables may limit its ability to fully capture the complexity of trauma severity beyond general critical illness indicators.

Perozziello et al. compared data from a medical records data with PMSI records and found that the GHM-26 classification is only partially effective in identifying severe trauma patients [4]. In their study, 35% of patients who did not have injuries coded under the GHM-26 classification had an ISS greater than 15, indicating that a substantial proportion of severe trauma cases were not adequately captured—an undertriage-like situation [4]. To address this limitation, and following the approach proposed by Bège et al. [1], we applied a refined version of the GHM-26 classification to improve patient selection and better identify severe trauma cases within the PMSI.

Historically, efforts to predict mortality using ICD data date back to 1996, when the ICD-9 Injury Severity Score (ICISS) achieved a performance with an area under the receiver-operating characteristic curve (AROC) of 0.92—outperforming the ISS, which had an AROC of 0.87 [21]. Similarly, another ICD-based injury severity score, originally developed in Victoria State, Australia, was designed to identify and monitor severe injuries. However, as these scores were derived from populations with mortality rates below 1%, their applicability to populations of severe trauma patients remains limited [22]. Moreover, evidence suggests that ICD-based models may not be directly generalizable across countries [23]. More recently, a study using the American National Trauma Data Bank, encompassing over one million patients, demonstrated that a machine learning model (eXtreme Gradient Boosting) achieved an AROC of 0.95 for mortality prediction, compared to 0.91 for TRISS [24].

In France, Bège et al. published a study in 2019 based on PMSI data [1]. Unlike our work, their objective was not to predict mortality but rather to describe a cohort of trauma patients using selection criteria inspired by the British Trauma Audit and Research Network (TARN) registry. Patients were identified in the PMSI based on criteria including a length of stay in hospital (LOS) of three days or more, ICD trauma codes, admission to the ICU, or death. Applying these criteria, the authors selected 144,058 severe trauma patients in 2016, providing the first comprehensive estimate of trauma case volume in France [1]. However, despite this large dataset, patient characterization remained imprecise, particularly regarding injury severity and lesion patterns, although authors attempted to address this issue using the ICD-9 Injury Severity Score. Finally, the RISC II score, developed within the DGU registry and shown to moderately outperform TRISS with an AUC of 0.953 versus 0.939, could have been used [25]. However, it was not applied because its performance in France has not yet been established.

The present study has several limitations. First, this is a monocentric study, and external validation of the models could not be conducted. Broader validation across multiple centers will therefore be necessary to confirm the robustness and generalizability of the model in diverse clinical settings. Second, despite centralized validation efforts, the accuracy of ICD-10 coding—particularly for traumatic injuries—may be questioned. For example, penetrating injuries appear to be poorly captured by the PMSI, with only 3% of patients identified as having a penetrating trauma in PMSI data compared with 19% in medical records. Additionally, the nature of shock is imprecisely documented in ICD-10 coding, whereas this variable is more accurately characterized in clinical registries. Moreover, the use of individual diagnostic codes to identify patients is insufficient to fully capture the complexity of clinical presentations and is therefore inherently imperfect. For example, an injury to a cervical artery may range from a minor dissection to a severe lesion requiring surgical intervention and associated with a life-threatening prognosis. Nevertheless, the objective of identifying a population with severe injuries appears to have been achieved, as reflected by a median Injury Severity Score of 19, a 28% rate of shock on admission and a median SAPS II of 30. Third, the usual data used to categorize patients as severely injured, such as the Injury Severity Score (ISS), are not available in the PMSI, which necessitated selecting patients based on diagnostic codes. This previously published method offers the advantage of being reproducible and transparent. The median ISS of the matched patients was 19 (IQR, 11–29), confirming that the selected population indeed consisted of severely injured trauma patients. Fourth, coding practices may vary significantly between centers, which could also impact the overall performance of the models. Fifth, frailty and other forms of patient vulnerability are poorly represented in ICD-10 coding, although they are highly relevant in severe trauma populations. Sixth, the prediction model relies heavily on the SAPS II score—a validated but not trauma-specific mortality score—and patient age. Consequently, the predicted mortality appears minimally influenced by trauma-specific factors, which may limit its specificity. Importantly, this model should not be interpreted as reproducing the TRISS itself, but rather as an estimation of a TRISS-like construct based on administrative data proxies. Finally, patients with matched data were significantly more severely ill than unmatched patients, which may restrict the external validity of the findings. However, the main value of this analysis lies in its applicability to severely injured patients rather than a broader, over-selected cohort.

## Conclusion

The proposed prediction model provides a patient-specific tool for assessing trauma severity and risk of death in France using PMSI data. This approach also demonstrates that a similar methodology could be applied to other administrative databases in different countries, enabling national-level studies to evaluate the quality of care delivered to severe trauma patients even when well-recognized trauma scores have not been recorded. Moreover, this method paves the way for the identification of severe trauma patients within the SNDS, enabling the conduct of longitudinal, population-based studies at the national level.

## Supporting information

S1 File Supplementary Materials. (DOCX)
