# Supplementary material for: Predicting the Trauma and Injury Severity Score (TRISS) in patients admitted to trauma centers after trauma: A modeling study based on data from the French National Programme for Medicalization of Information
Source: PLoS One. 2026 Aug 17;21(8):e0354700. doi: 10.1371/journal.pone.0354700 (PMC13480638; doi:10.1371/journal.pone.0354700)
Supplement: Supplementary file 1 [file pone.0354700.s001.docx]

# Supplementary Materials

## **Supplementary Material 1: List of ICD-10 codes that categorize a patient as having severe trauma**. Codes associated with non-severe trauma alone are not sufficient to classify a patient as having severe trauma. The presence of two intermediate codes is required to categorize a patient as having severe trauma. The presence of a severe code categorizes a patient as having severe trauma. Classification into GHM-26 occurs if the primary diagnosis is part of the D-130 list (the diagnostic codes for traumatic injuries, whether severe or not) and if the primary or associated diagnosis falls within the D-131 to D-138 lists (sub-lists of D-130, which refer to codes for severe trauma by anatomical site). However, the D-131 to D-138 lists are sometimes imprecise because they are too general, leading to an overclassification of patients as severely injured. For example, the system categorizes any patient with a simple pneumothorax as severely injured if it is associated with any other injury coded in the D-130 list (including less severe conditions such as a sprained limb or a finger fracture). Conversely, some diagnoses might be considered severe trauma by experts, even if not associated with another diagnosis. Therefore, in accordance with the method proposed by Bege et al., a modified list of codes was developed from the GHM-26 list[12]. Four experts were involved in recategorizing the GHM-26 diagnoses. These experts were experienced anesthesiologists and intensivists accustomed to treating severely injured trauma patients and performing coding during the initial management of severe trauma patients. They were asked to independently categorize each GHM-26 diagnosis to refine the selection of severely injured patients. After this step, one investigator (AJ) synthesized the responses, and discrepancies were resolved through discussion, involving an additional expert (MR) when necessary. Thus, each GHM-26 code was associated with one of the following trauma severities: “Non-severe”, “Intermediate”, or “Severe”. The presence of two intermediate codes for the same patient led to the classification of the trauma as “Severe”. For example, a superficial nasal injury or a limb sprain were categorized as “Non-severe”, a fracture of the maxilla or sternum as “Intermediate”, and a flail chest as “Severe”. Therefore, a maxillary fracture associated with a sternal fracture would be classified as “Severe”.

| **Code ICD-1** | **Name** | **Severity** | Head & Neck | Face | Skin | Chest | Abdo-  minal | Upper extre-  mities | Lower extre-  mities | Non catago-  rized |
| --- | --- | --- | --- | --- | --- | --- | --- | --- | --- | --- |
| S00.0 | Superficial scalp injury | Non severe | 0 | 0 | 1 | 0 | 0 | 0 | 0 | 0 |
| S00.1 | Contusion of eyelid and periocular region | Non severe | 0 | 1 | 0 | 0 | 0 | 0 | 0 | 0 |
| S00.2 | Superficial injury of eyelid and periocular region, NEC | Non severe | 0 | 0 | 1 | 0 | 0 | 0 | 0 | 0 |
| S00.3 | Superficial injury of the nose | Non severe | 0 | 0 | 1 | 0 | 0 | 0 | 0 | 0 |
| S00.4 | Superficial injury of the ear | Non severe | 0 | 0 | 1 | 0 | 0 | 0 | 0 | 0 |
| S00.5 | Superficial injury of the lip and oral cavity | Non severe | 0 | 0 | 1 | 0 | 0 | 0 | 0 | 0 |
| S00.7 | Multiple superficial injuries of the head | Non severe | 0 | 0 | 1 | 0 | 0 | 0 | 0 | 0 |
| S00.8 | Superficial injury of other parts of the head | Non severe | 0 | 0 | 1 | 0 | 0 | 0 | 0 | 0 |
| S01.0 | Open wound of the scalp | Non severe | 0 | 1 | 0 | 0 | 0 | 0 | 0 | 0 |
| S01.1 | Open wound of the eyelid and periocular region | Non severe | 0 | 1 | 0 | 0 | 0 | 0 | 0 | 0 |
| S01.2 | Open wound of the nose | Non severe | 0 | 1 | 0 | 0 | 0 | 0 | 0 | 0 |
| S01.3 | Open wound of the ear | Non severe | 0 | 1 | 0 | 0 | 0 | 0 | 0 | 0 |
| S01.4 | Open wound of the cheek and temporomaxillary region | Non severe | 0 | 1 | 0 | 0 | 0 | 0 | 0 | 0 |
| S01.5 | Open wound of the lip and oral cavity | Non severe | 0 | 1 | 0 | 0 | 0 | 0 | 0 | 0 |
| S01.7 | Multiple open wounds of the head | Mild | 1 | 1 | 0 | 0 | 0 | 0 | 0 | 0 |
| S01.8 | Open wound of other parts of the head | Non severe | 1 | 1 | 0 | 0 | 0 | 0 | 0 | 0 |
| S02.00 | Closed fracture of the vault of the skull | Mild | 1 | 0 | 0 | 0 | 0 | 0 | 0 | 0 |
| S02.01 | Open fracture of the vault of the skull | Severe | 1 | 0 | 0 | 0 | 0 | 0 | 0 | 0 |
| S02.10 | Closed fracture of the base of the skull | Mild | 1 | 0 | 0 | 0 | 0 | 0 | 0 | 0 |
| S02.11 | Open fracture of the base of the skull | Severe | 1 | 0 | 0 | 0 | 0 | 0 | 0 | 0 |
| S02.20 | Closed fracture of the nasal bones | Non severe | 0 | 1 | 0 | 0 | 0 | 0 | 0 | 0 |
| S02.21 | Open fracture of the nasal bones | Non severe | 0 | 1 | 0 | 0 | 0 | 0 | 0 | 0 |
| S02.30 | Closed fracture of the orbital floor | Non severe | 0 | 1 | 0 | 0 | 0 | 0 | 0 | 0 |
| S02.31 | Open fracture of the orbital floor | Mild | 0 | 1 | 0 | 0 | 0 | 0 | 0 | 0 |
| S02.40 | Closed fracture of the zygomatic and maxillary bones | Non severe | 0 | 1 | 0 | 0 | 0 | 0 | 0 | 0 |
| S02.41 | Open fracture of the zygomatic and maxillary bones | Non severe | 0 | 1 | 0 | 0 | 0 | 0 | 0 | 0 |
| S02.5 | Dental fracture | Non severe | 0 | 1 | 0 | 0 | 0 | 0 | 0 | 0 |
| S02.50 | Closed dental fracture | Non severe | 0 | 1 | 0 | 0 | 0 | 0 | 0 | 0 |
| S02.51 | Open dental fracture | Non severe | 0 | 1 | 0 | 0 | 0 | 0 | 0 | 0 |
| S02.60 | Closed fracture of the lower jaw | Non severe | 0 | 1 | 0 | 0 | 0 | 0 | 0 | 0 |
| S02.61 | Open fracture of the lower jaw | Mild | 0 | 1 | 0 | 0 | 0 | 0 | 0 | 0 |
| S02.70 | Closed multiple fractures of the skull and facial bones | Mild | 1 | 1 | 0 | 0 | 0 | 0 | 0 | 0 |
| S02.71 | Open multiple fractures of the skull and facial bones | Severe | 1 | 1 | 0 | 0 | 0 | 0 | 0 | 0 |
| S02.80 | Closed fractures of other bones of the skull and face | Non severe | 1 | 1 | 0 | 0 | 0 | 0 | 0 | 0 |
| S02.81 | Open fractures of other bones of the skull and face | Non severe | 1 | 1 | 0 | 0 | 0 | 0 | 0 | 0 |
| S03.0 | Dislocation of the jaw | Non severe | 0 | 1 | 0 | 0 | 0 | 0 | 0 | 0 |
| S03.1 | Nasal septum luxation | Mild | 0 | 1 | 0 | 0 | 0 | 0 | 0 | 0 |
| S03.2 | Dental luxation | Mild | 0 | 1 | 0 | 0 | 0 | 0 | 0 | 0 |
| S03.3 | Luxation of parts of the head (not coded elsewhere or Without further information) | Mild | 1 | 1 | 0 | 0 | 0 | 0 | 0 | 0 |
| S03.4 | Jaw sprain | Mild | 0 | 1 | 0 | 0 | 0 | 0 | 0 | 0 |
| S04.0 | Trauma to the optic nerve and pathways | Mild | 1 | 1 | 0 | 0 | 0 | 0 | 0 | 0 |
| S04.1 | Trauma to the oculomotor nerve | Mild | 1 | 0 | 0 | 0 | 0 | 0 | 0 | 0 |
| S04.2 | Trauma to the trochlear nerve | Mild | 1 | 0 | 0 | 0 | 0 | 0 | 0 | 0 |
| S04.3 | Trauma to the trigeminal nerve | Mild | 1 | 0 | 0 | 0 | 0 | 0 | 0 | 0 |
| S04.4 | Trauma to the external oculomotor nerve | Mild | 1 | 0 | 0 | 0 | 0 | 0 | 0 | 0 |
| S04.5 | Trauma to the facial nerve | Mild | 0 | 1 | 0 | 0 | 0 | 0 | 0 | 0 |
| S04.6 | Trauma to the auditory nerve | Mild | 1 | 0 | 0 | 0 | 0 | 0 | 0 | 0 |
| S04.7 | Trauma to the spinal nerve | Mild | 1 | 0 | 0 | 0 | 0 | 0 | 0 | 0 |
| S04.8 | Trauma to other cranial nerves | Mild | 1 | 0 | 0 | 0 | 0 | 0 | 0 | 0 |
| S04.9 | Trauma to a cranial nerve, Without further information | Mild | 1 | 0 | 0 | 0 | 0 | 0 | 0 | 0 |
| S05.0 | Conjunctival injury and corneal abrasion without mention of foreign body | Mild | 0 | 1 | 0 | 0 | 0 | 0 | 0 | 0 |
| S05.1 | Contusion of the eyeball and orbital tissues | Mild | 0 | 1 | 0 | 0 | 0 | 0 | 0 | 0 |
| S05.2 | Laceration and rupture of the eye with intraocular tissue loss | Mild | 0 | 1 | 0 | 0 | 0 | 0 | 0 | 0 |
| S05.3 | Ocular laceration | Mild | 0 | 1 | 0 | 0 | 0 | 0 | 0 | 0 |
| S05.4 | Penetrating wound of the orbit, with or without foreign body | Mild | 0 | 1 | 0 | 0 | 0 | 0 | 0 | 0 |
| S05.5 | Penetrating wound of the eyeball, with foreign body | Mild | 0 | 1 | 0 | 0 | 0 | 0 | 0 | 0 |
| S05.6 | Penetrating wound of the eyeball, Without further information | Mild | 0 | 1 | 0 | 0 | 0 | 0 | 0 | 0 |
| S05.7 | Eye avulsion | Severe | 0 | 1 | 0 | 0 | 0 | 0 | 0 | 0 |
| S05.8 | Trauma to the eye and orbit, not coded elsewhere | Mild | 0 | 1 | 0 | 0 | 0 | 0 | 0 | 0 |
| S05.9 | Trauma to the eye and orbit, Without further information | Mild | 0 | 1 | 0 | 0 | 0 | 0 | 0 | 0 |
| S06.0 | Concussion | Mild | 1 | 0 | 0 | 0 | 0 | 0 | 0 | 0 |
| S06.00 | Concussion, without intracranial injury | Mild | 1 | 0 | 0 | 0 | 0 | 0 | 0 | 0 |
| S06.01 | Concussion, with intracranial injury | Severe | 1 | 0 | 0 | 0 | 0 | 0 | 0 | 0 |
| S06.1 | Cerebral edema trauma | Severe | 1 | 0 | 0 | 0 | 0 | 0 | 0 | 0 |
| S06.10 | Cerebral edema trauma, without intracranial injury | Severe | 1 | 0 | 0 | 0 | 0 | 0 | 0 | 0 |
| S06.11 | Cerebral edema trauma, with intracranial injury | Severe | 1 | 0 | 0 | 0 | 0 | 0 | 0 | 0 |
| S06.2 | Diffuse cerebral trauma | Severe | 1 | 0 | 0 | 0 | 0 | 0 | 0 | 0 |
| S06.20 | Diffuse cerebral trauma, without intracranial injury | Severe | 1 | 0 | 0 | 0 | 0 | 0 | 0 | 0 |
| S06.21 | Diffuse cerebral trauma, with intracranial injury | Severe | 1 | 0 | 0 | 0 | 0 | 0 | 0 | 0 |
| S06.3 | Focal cerebral trauma | Severe | 1 | 0 | 0 | 0 | 0 | 0 | 0 | 0 |
| S06.30 | Focal cerebral trauma, without intracranial injury | Severe | 1 | 0 | 0 | 0 | 0 | 0 | 0 | 0 |
| S06.31 | Focal cerebral trauma, with intracranial injury | Severe | 1 | 0 | 0 | 0 | 0 | 0 | 0 | 0 |
| S06.4 | Epidural hemorrhage | Severe | 1 | 0 | 0 | 0 | 0 | 0 | 0 | 0 |
| S06.40 | Epidural hemorrhage, without intracranial injury | Severe | 1 | 0 | 0 | 0 | 0 | 0 | 0 | 0 |
| S06.41 | Epidural hemorrhage, with intracranial injury | Severe | 1 | 0 | 0 | 0 | 0 | 0 | 0 | 0 |
| S06.5 | Subdural hemorrhage trauma | Severe | 1 | 0 | 0 | 0 | 0 | 0 | 0 | 0 |
| S06.50 | Subdural hemorrhage trauma, without intracranial injury | Severe | 1 | 0 | 0 | 0 | 0 | 0 | 0 | 0 |
| S06.51 | Subdural hemorrhage trauma, with intracranial injury | Severe | 1 | 0 | 0 | 0 | 0 | 0 | 0 | 0 |
| S06.6 | Subarachnoid hemorrhage trauma | Mild | 1 | 0 | 0 | 0 | 0 | 0 | 0 | 0 |
| S06.60 | Subarachnoid hemorrhage trauma, without intracranial injury | Mild | 1 | 0 | 0 | 0 | 0 | 0 | 0 | 0 |
| S06.61 | Subarachnoid hemorrhage trauma, with intracranial injury | Severe | 1 | 0 | 0 | 0 | 0 | 0 | 0 | 0 |
| S06.7 | Intracranial trauma with prolonged coma | Severe | 1 | 0 | 0 | 0 | 0 | 0 | 0 | 0 |
| S06.70 | Intracranial trauma with prolonged coma, without intracranial injury | Severe | 1 | 0 | 0 | 0 | 0 | 0 | 0 | 0 |
| S06.71 | Intracranial trauma with prolonged coma, with intracranial injury | Severe | 1 | 0 | 0 | 0 | 0 | 0 | 0 | 0 |
| S06.8 | Intracranial trauma, not coded elsewhere | Mild | 1 | 0 | 0 | 0 | 0 | 0 | 0 | 0 |
| S06.80 | Intracranial trauma, not coded elsewhere, without intracranial injury | Mild | 1 | 0 | 0 | 0 | 0 | 0 | 0 | 0 |
| S06.81 | Intracranial trauma, not coded elsewhere, with intracranial injury | Mild | 1 | 0 | 0 | 0 | 0 | 0 | 0 | 0 |
| S06.9 | Intracranial trauma, Without further information | Mild | 1 | 0 | 0 | 0 | 0 | 0 | 0 | 0 |
| S06.90 | Intracranial trauma, Without further information, without intracranial injury | Mild | 1 | 0 | 0 | 0 | 0 | 0 | 0 | 0 |
| S06.91 | Intracranial trauma, Without further information, with intracranial injury | Mild | 1 | 0 | 0 | 0 | 0 | 0 | 0 | 0 |
| S07.0 | Face crushing injury | Severe | 0 | 1 | 0 | 0 | 0 | 0 | 0 | 0 |
| S07.1 | Skull crushing injury | Severe | 1 | 0 | 0 | 0 | 0 | 0 | 0 | 0 |
| S07.8 | Crushing injury to other parts of the head | Severe | 1 | 1 | 0 | 0 | 0 | 0 | 0 | 0 |
| S07.9 | Crushing injury of the head, part Without further information | Mild | 1 | 1 | 0 | 0 | 0 | 0 | 0 | 0 |
| S08.0 | Scalp avulsion | Mild | 1 | 0 | 0 | 0 | 0 | 0 | 0 | 0 |
| S08.1 | Ear amputation trauma | Mild | 0 | 1 | 0 | 0 | 0 | 0 | 0 | 0 |
| S08.8 | Amputations of other parts of the head | Mild | 1 | 1 | 0 | 0 | 0 | 0 | 0 | 0 |
| S08.9 | Amputations of a part of the head, Without further information | Mild | 1 | 1 | 0 | 0 | 0 | 0 | 0 | 0 |
| S09.0 | Trauma to blood vessels in the head, not coded elsewhere | Mild | 1 | 1 | 0 | 0 | 0 | 0 | 0 | 0 |
| S09.1 | Trauma to muscles and tendons of the head | Mild | 1 | 1 | 0 | 0 | 0 | 0 | 0 | 0 |
| S09.2 | Rupture of the eardrum | Mild | 0 | 1 | 0 | 0 | 0 | 0 | 0 | 0 |
| S09.7 | Multiple trauma to the head | Mild | 1 | 1 | 0 | 0 | 0 | 0 | 0 | 0 |
| S09.8 | Trauma to the head, specific, not coded elsewhere | Mild | 1 | 1 | 0 | 0 | 0 | 0 | 0 | 0 |
| S10.0 | Contusion of the throat | Mild | 1 | 0 | 0 | 0 | 0 | 0 | 0 | 0 |
| S10.1 | Superficial trauma to the throat, not coded elsewhere or Without further information | Mild | 1 | 0 | 0 | 0 | 0 | 0 | 0 | 0 |
| S10.7 | Multiple superficial trauma to the neck | Mild | 1 | 0 | 0 | 0 | 0 | 0 | 0 | 0 |
| S10.8 | Superficial trauma to other parts of the neck | Mild | 1 | 0 | 0 | 0 | 0 | 0 | 0 | 0 |
| S10.9 | Superficial trauma to the neck, Without further information | Mild | 1 | 0 | 0 | 0 | 0 | 0 | 0 | 0 |
| S11.0 | Open wound with injury to the larynx and trachea | Severe | 1 | 0 | 0 | 1 | 0 | 0 | 0 | 0 |
| S11.1 | Open wound with injury to the thyroid | Mild | 1 | 0 | 0 | 0 | 0 | 0 | 0 | 0 |
| S11.2 | Open wound with injury to the pharynx and cervical esophagus | Severe | 1 | 0 | 0 | 0 | 0 | 0 | 0 | 0 |
| S11.7 | Multiple open wounds of the neck | Mild | 1 | 0 | 0 | 0 | 0 | 0 | 0 | 0 |
| S11.8 | Open wound of other parts of the neck | Mild | 1 | 0 | 0 | 0 | 0 | 0 | 0 | 0 |
| S11.9 | Open wound of the neck, part Without further information | Mild | 1 | 0 | 0 | 0 | 0 | 0 | 0 | 0 |
| S12.00 | Closed fracture of the 1st cervical vertebra | Mild | 1 | 0 | 0 | 0 | 0 | 0 | 0 | 0 |
| S12.01 | Open fracture of the 1st cervical vertebra | Mild | 1 | 0 | 0 | 0 | 0 | 0 | 0 | 0 |
| S12.10 | Closed fracture of the 2nd cervical vertebra | Mild | 1 | 0 | 0 | 0 | 0 | 0 | 0 | 0 |
| S12.11 | Open fracture of the 2nd cervical vertebra | Mild | 1 | 0 | 0 | 0 | 0 | 0 | 0 | 0 |
| S12.20 | Closed fracture of other cervical vertebrae | Mild | 1 | 0 | 0 | 0 | 0 | 0 | 0 | 0 |
| S12.21 | Open fracture of other cervical vertebrae | Mild | 1 | 0 | 0 | 0 | 0 | 0 | 0 | 0 |
| S12.70 | Multiple closed fractures of the cervical spine | Mild | 1 | 0 | 0 | 0 | 0 | 0 | 0 | 0 |
| S12.71 | Open multiple fractures of the cervical spine | Severe | 1 | 0 | 0 | 0 | 0 | 0 | 0 | 0 |
| S12.80 | Closed fracture of other parts of the neck | Mild | 1 | 0 | 0 | 0 | 0 | 0 | 0 | 0 |
| S12.81 | Open fracture of other parts of the neck | Mild | 1 | 0 | 0 | 0 | 0 | 0 | 0 | 0 |
| S12.90 | Closed fracture of the neck, part Without further information | Mild | 1 | 0 | 0 | 0 | 0 | 0 | 0 | 0 |
| S12.91 | Open fracture of the neck, part Without further information | Mild | 1 | 0 | 0 | 0 | 0 | 0 | 0 | 0 |
| S13.0 | Rupture of a cervical intervertebral disc | Mild | 1 | 0 | 0 | 0 | 0 | 0 | 0 | 0 |
| S13.1 | Luxation of a cervical vertebra | Mild | 1 | 0 | 0 | 0 | 0 | 0 | 0 | 0 |
| S13.2 | Luxation of parts of the neck (not coded elsewhere or Without further information) | Mild | 1 | 0 | 0 | 0 | 0 | 0 | 0 | 0 |
| S13.3 | Multiple luxations of the neck | Mild | 1 | 0 | 0 | 0 | 0 | 0 | 0 | 0 |
| S13.4 | Cervical spine sprain | Mild | 1 | 0 | 0 | 0 | 0 | 0 | 0 | 0 |
| S13.5 | Sprain of the thyroid region | Mild | 1 | 0 | 0 | 0 | 0 | 0 | 0 | 0 |
| S13.6 | Sprain of the joints and ligaments of parts of the neck (not coded elsewhere or Without further information) | Mild | 1 | 0 | 0 | 0 | 0 | 0 | 0 | 0 |
| S14.0 | Concussion and edema of the cervical spinal cord | Severe | 1 | 0 | 0 | 0 | 0 | 0 | 0 | 0 |
| S14.1 | Trauma to the cervical spinal cord, not coded elsewhere or Without further information | Severe | 1 | 0 | 0 | 0 | 0 | 0 | 0 | 0 |
| S14.2 | TRAUMA OF A CERVICAL SPINAL ROOT | Non severe | 1 | 0 | 0 | 0 | 0 | 0 | 0 | 0 |
| S14.3 | TRAUMA OF THE BRACHIAL PLEXUS | Non severe | 1 | 0 | 0 | 0 | 0 | 0 | 0 | 0 |
| S14.4 | TRAUMA OF THE PERIPHERAL NERVES OF THE NECK | Non severe | 1 | 0 | 0 | 0 | 0 | 0 | 0 | 0 |
| S14.5 | TRAUMA OF THE CERVICAL SYMPATHETIC NERVES | Non severe | 1 | 0 | 0 | 0 | 0 | 0 | 0 | 0 |
| S14.6 | TRAUMA OF THE NECK NERVES, not coded elsewhere OR Without further information | Non severe | 1 | 0 | 0 | 0 | 0 | 0 | 0 | 0 |
| S15.0 | TRAUMA OF THE CAROTID ARTERY | Severe | 1 | 0 | 0 | 0 | 0 | 0 | 0 | 0 |
| S15.1 | TRAUMA OF THE VERTEBRAL ARTERY | Severe | 1 | 0 | 0 | 0 | 0 | 0 | 0 | 0 |
| S15.2 | TRAUMA OF THE EXTERNAL JUGULAR VEIN | Mild | 1 | 0 | 0 | 0 | 0 | 0 | 0 | 0 |
| S15.3 | TRAUMA OF THE INTERNAL JUGULAR VEIN | Severe | 1 | 0 | 0 | 0 | 0 | 0 | 0 | 0 |
| S15.7 | TRAUMA OF MULTIPLE BLOOD VESSELS IN THE NECK | Mild | 1 | 0 | 0 | 0 | 0 | 0 | 0 | 0 |
| S15.8 | TRAUMA OF OTHER BLOOD VESSELS IN THE NECK | Non severe | 1 | 0 | 0 | 0 | 0 | 0 | 0 | 0 |
| S15.9 | TRAUMA OF BLOOD VESSELS IN THE NECK, Without further information | Non severe | 1 | 0 | 0 | 0 | 0 | 0 | 0 | 0 |
| S16 | TRAUMA OF THE MUSCLES AND TENDONS IN THE NECK | Non severe | 1 | 0 | 0 | 0 | 0 | 0 | 0 | 0 |
| S17.0 | CRUSHING OF THE LARYNX AND TRACHEA | Severe | 1 | 0 | 0 | 0 | 0 | 0 | 0 | 0 |
| S17.8 | CRUSHING OF OTHER PARTS OF THE NECK | Mild | 1 | 0 | 0 | 0 | 0 | 0 | 0 | 0 |
| S17.9 | CRUSHING OF THE NECK, Without further information PART | Mild | 1 | 0 | 0 | 0 | 0 | 0 | 0 | 0 |
| S18 | AMPUTATION TRAUMA OF THE NECK | Severe | 1 | 0 | 0 | 0 | 0 | 0 | 0 | 0 |
| S19.7 | MULTIPLE TRAUMA OF THE NECK | Mild | 1 | 0 | 0 | 0 | 0 | 0 | 0 | 0 |
| S19.8 | TRAUMA OF THE NECK, PRECISION not coded elsewhere | Non severe | 1 | 0 | 0 | 0 | 0 | 0 | 0 | 0 |
| S20.0 | CONTUSION OF THE BREAST | Non severe | 0 | 0 | 1 | 0 | 0 | 0 | 0 | 0 |
| S20.1 | SUPERFICIAL TRAUMA OF THE BREAST, not coded elsewhere OR Without further information | Non severe | 0 | 0 | 1 | 0 | 0 | 0 | 0 | 0 |
| S20.2 | CONTUSION OF THE CHEST | Non severe | 0 | 0 | 0 | 1 | 0 | 0 | 0 | 0 |
| S20.3 | SUPERFICIAL TRAUMA OF THE ANTERIOR THORACIC WALL, not coded elsewhere | Non severe | 0 | 0 | 1 | 0 | 0 | 0 | 0 | 0 |
| S20.4 | SUPERFICIAL TRAUMA OF THE POSTERIOR THORACIC WALL, not coded elsewhere | Non severe | 0 | 0 | 1 | 0 | 0 | 0 | 0 | 0 |
| S20.7 | MULTIPLE SUPERFICIAL TRAUMA OF THE CHEST | Non severe | 0 | 0 | 1 | 0 | 0 | 0 | 0 | 0 |
| S20.8 | SUPERFICIAL TRAUMA OF OTHER not coded elsewhere OR Without further information PARTS OF THE CHEST | Non severe | 0 | 0 | 1 | 0 | 0 | 0 | 0 | 0 |
| S21.0 | OPEN WOUND OF THE BREAST | Non severe | 0 | 0 | 0 | 1 | 0 | 0 | 0 | 0 |
| S21.1 | OPEN WOUND OF THE ANTERIOR THORACIC WALL | Non severe | 0 | 0 | 0 | 1 | 0 | 0 | 0 | 0 |
| S21.2 | OPEN WOUND OF THE POSTERIOR THORACIC WALL | Non severe | 0 | 0 | 0 | 1 | 0 | 0 | 0 | 0 |
| S21.7 | MULTIPLE OPEN WOUNDS OF THE THORACIC WALL | Mild | 0 | 0 | 0 | 1 | 0 | 0 | 0 | 0 |
| S21.8 | OPEN WOUNDS OF OTHER PARTS OF THE CHEST | Mild | 0 | 0 | 0 | 1 | 0 | 0 | 0 | 0 |
| S21.9 | OPEN WOUND OF THE CHEST, Without further information | Non severe | 0 | 0 | 0 | 1 | 0 | 0 | 0 | 0 |
| S22.00 | CLOSED FRACTURE OF A DORSAL VERTEBRA | Non severe | 0 | 0 | 0 | 1 | 0 | 0 | 0 | 0 |
| S22.01 | OPEN FRACTURE OF A DORSAL VERTEBRA | Mild | 0 | 0 | 0 | 1 | 0 | 0 | 0 | 0 |
| S22.10 | MULTIPLE CLOSED FRACTURES OF THE DORSAL SPINE | Mild | 0 | 0 | 0 | 1 | 0 | 0 | 0 | 0 |
| S22.11 | MULTIPLE OPEN FRACTURES OF THE DORSAL SPINE | Severe | 0 | 0 | 0 | 1 | 0 | 0 | 0 | 0 |
| S22.20 | CLOSED FRACTURE OF THE STERNUM | Mild | 0 | 0 | 0 | 1 | 0 | 0 | 0 | 0 |
| S22.21 | OPEN FRACTURE OF THE STERNUM | Mild | 0 | 0 | 0 | 1 | 0 | 0 | 0 | 0 |
| S22.30 | CLOSED FRACTURE OF A RIB | Non severe | 0 | 0 | 0 | 1 | 0 | 0 | 0 | 0 |
| S22.31 | OPEN FRACTURE OF A RIB | Non severe | 0 | 0 | 0 | 1 | 0 | 0 | 0 | 0 |
| S22.40 | MULTIPLE CLOSED RIB FRACTURES | Mild | 0 | 0 | 0 | 1 | 0 | 0 | 0 | 0 |
| S22.41 | MULTIPLE OPEN RIB FRACTURES | Mild | 0 | 0 | 0 | 1 | 0 | 0 | 0 | 0 |
| S22.50 | COSTAL FLAIL CHEST WITH CLOSED FRACTURE | Severe | 0 | 0 | 0 | 1 | 0 | 0 | 0 | 0 |
| S22.51 | COSTAL FLAIL CHEST WITH OPEN FRACTURE | Severe | 0 | 0 | 0 | 1 | 0 | 0 | 0 | 0 |
| S22.80 | CLOSED FRACTURE OF OTHER BONES OF THE CHEST | Non severe | 0 | 0 | 0 | 1 | 0 | 0 | 0 | 0 |
| S22.81 | OPEN FRACTURE OF OTHER BONES OF THE CHEST | Non severe | 0 | 0 | 0 | 1 | 0 | 0 | 0 | 0 |
| S23.0 | TRAUMA OF A DORSAL INTERVERTEBRAL DISC | Non severe | 0 | 0 | 0 | 1 | 0 | 0 | 0 | 0 |
| S23.1 | DISLOCATION OF A DORSAL VERTEBRA | Mild | 0 | 0 | 0 | 1 | 0 | 0 | 0 | 0 |
| S23.2 | DISLOCATION OF not coded elsewhere OR Without further information PARTS OF THE CHEST | Non severe | 0 | 0 | 0 | 1 | 0 | 0 | 0 | 0 |
| S23.3 | SPRAIN OF THE DORSAL SPINE | Non severe | 0 | 0 | 0 | 1 | 0 | 0 | 0 | 0 |
| S23.4 | SPRAIN OF THE RIBS AND STERNUM | Non severe | 0 | 0 | 0 | 1 | 0 | 0 | 0 | 0 |
| S23.5 | SPRAIN OF not coded elsewhere OR Without further information PARTS OF THE CHEST | Non severe | 0 | 0 | 0 | 1 | 0 | 0 | 0 | 0 |
| S24.0 | Concussion and edema of the dorsal spinal cord | Severe | 0 | 0 | 0 | 1 | 0 | 0 | 0 | 0 |
| S24.1 | Non-complicated or complicated spinal cord injury | Mild | 0 | 0 | 0 | 1 | 0 | 0 | 0 | 0 |
| S24.2 | Nerve root injury of the dorsal spine | Non severe | 0 | 0 | 0 | 1 | 0 | 0 | 0 | 0 |
| S24.3 | Peripheral nerve injury in the thoracic region | Non severe | 0 | 0 | 0 | 1 | 0 | 0 | 0 | 0 |
| S24.4 | Sympathetic nerve injury in the thoracic region | Mild | 0 | 0 | 0 | 1 | 0 | 0 | 0 | 0 |
| S24.5 | Injury to other nerves in the thoracic region | Non severe | 0 | 0 | 0 | 1 | 0 | 0 | 0 | 0 |
| S24.6 | Injury to a sympathetic nerve in the thoracic region | Non severe | 0 | 0 | 0 | 1 | 0 | 0 | 0 | 0 |
| S25.0 | Injury to the thoracic aorta | Severe | 0 | 0 | 0 | 1 | 0 | 0 | 0 | 0 |
| S25.1 | Injury to the innominate or subclavian artery | Severe | 0 | 0 | 0 | 1 | 0 | 0 | 0 | 0 |
| S25.2 | Injury to the superior vena cava | Severe | 0 | 0 | 0 | 1 | 0 | 0 | 0 | 0 |
| S25.3 | Injury to the innominate or subclavian vein | Mild | 0 | 0 | 0 | 1 | 0 | 0 | 0 | 0 |
| S25.4 | Injury to blood vessels in the lung | Severe | 0 | 0 | 0 | 1 | 0 | 0 | 0 | 0 |
| S25.5 | Injury to blood vessels in the intercostal space | Mild | 0 | 0 | 0 | 1 | 0 | 0 | 0 | 0 |
| S25.7 | Injury to multiple blood vessels in the thorax | Severe | 0 | 0 | 0 | 1 | 0 | 0 | 0 | 0 |
| S25.80 | Injury to the azygos vein | Mild | 0 | 0 | 0 | 1 | 0 | 0 | 0 | 0 |
| S25.88 | Injury to other blood vessels in the thorax (except azygos) | Mild | 0 | 0 | 0 | 1 | 0 | 0 | 0 | 0 |
| S25.9 | Injury to a blood vessel in the thorax | Mild | 0 | 0 | 0 | 1 | 0 | 0 | 0 | 0 |
| S26.0 | Injury to the heart with hemopericardium | Severe | 0 | 0 | 0 | 1 | 0 | 0 | 0 | 0 |
| S26.00 | Injury to the heart with hemopericardium, no intrathoracic injury | Severe | 0 | 0 | 0 | 1 | 0 | 0 | 0 | 0 |
| S26.01 | Injury to the heart with hemopericardium and intrathoracic injury | Severe | 0 | 0 | 0 | 1 | 0 | 0 | 0 | 0 |
| S26.8 | Non-complicated injury to the heart | Severe | 0 | 0 | 0 | 1 | 0 | 0 | 0 | 0 |
| S26.80 | Non-complicated heart injury, no intrathoracic injury | Severe | 0 | 0 | 0 | 1 | 0 | 0 | 0 | 0 |
| S26.81 | Non-complicated heart injury, with intrathoracic injury | Severe | 0 | 0 | 0 | 1 | 0 | 0 | 0 | 0 |
| S26.9 | Injury to the heart, sympathetic | Severe | 0 | 0 | 0 | 1 | 0 | 0 | 0 | 0 |
| S26.90 | Injury to the heart, sympathetic, no intrathoracic injury | Severe | 0 | 0 | 0 | 1 | 0 | 0 | 0 | 0 |
| S26.91 | Injury to the heart, sympathetic, with intrathoracic injury | Severe | 0 | 0 | 0 | 1 | 0 | 0 | 0 | 0 |
| S27.0 | Traumatic pneumothorax | Severe | 0 | 0 | 0 | 1 | 0 | 0 | 0 | 0 |
| S27.00 | Traumatic pneumothorax, no intrathoracic injury | Mild | 0 | 0 | 0 | 1 | 0 | 0 | 0 | 0 |
| S27.01 | Traumatic pneumothorax, with intrathoracic injury | Mild | 0 | 0 | 0 | 1 | 0 | 0 | 0 | 0 |
| S27.1 | Traumatic hemothorax | Mild | 0 | 0 | 0 | 1 | 0 | 0 | 0 | 0 |
| S27.10 | Traumatic hemothorax, no intrathoracic injury | Mild | 0 | 0 | 0 | 1 | 0 | 0 | 0 | 0 |
| S27.11 | Traumatic hemothorax, with intrathoracic injury | Severe | 0 | 0 | 0 | 1 | 0 | 0 | 0 | 0 |
| S27.2 | Hemopneumothorax trauma | Mild | 0 | 0 | 0 | 1 | 0 | 0 | 0 | 0 |
| S27.20 | Hemopneumothorax trauma, without intrathoracic injury | Mild | 0 | 0 | 0 | 1 | 0 | 0 | 0 | 0 |
| S27.21 | Hemopneumothorax trauma, with intrathoracic injury | Severe | 0 | 0 | 0 | 1 | 0 | 0 | 0 | 0 |
| S27.3 | Non-complicated lung injury | Mild | 0 | 0 | 0 | 1 | 0 | 0 | 0 | 0 |
| S27.30 | Non-complicated lung injury, without intrathoracic injury | Non severe | 0 | 0 | 0 | 1 | 0 | 0 | 0 | 0 |
| S27.31 | Non-complicated lung injury, with intrathoracic injury | Mild | 0 | 0 | 0 | 1 | 0 | 0 | 0 | 0 |
| S27.4 | Bronchial injury | Mild | 0 | 0 | 0 | 1 | 0 | 0 | 0 | 0 |
| S27.40 | Bronchial injury, without intrathoracic injury | Mild | 0 | 0 | 0 | 1 | 0 | 0 | 0 | 0 |
| S27.41 | Bronchial injury, with intrathoracic injury | Severe | 0 | 0 | 0 | 1 | 0 | 0 | 0 | 0 |
| S27.5 | Tracheal injury | Severe | 0 | 0 | 0 | 1 | 0 | 0 | 0 | 0 |
| S27.50 | Tracheal injury, without intrathoracic injury | Severe | 0 | 0 | 0 | 1 | 0 | 0 | 0 | 0 |
| S27.51 | Tracheal injury, with intrathoracic injury | Severe | 0 | 0 | 0 | 1 | 0 | 0 | 0 | 0 |
| S27.6 | Pleural injury | Mild | 0 | 0 | 0 | 1 | 0 | 0 | 0 | 0 |
| S27.60 | Pleural injury, without intrathoracic injury | Mild | 0 | 0 | 0 | 1 | 0 | 0 | 0 | 0 |
| S27.61 | Pleural injury, with intrathoracic injury | Mild | 0 | 0 | 0 | 1 | 0 | 0 | 0 | 0 |
| S27.7 | Multiple injuries to intrathoracic organs | Non severe | 0 | 0 | 0 | 1 | 0 | 0 | 0 | 0 |
| S27.70 | Multiple injuries to intrathoracic organs, without intrathoracic injury | Non severe | 0 | 0 | 0 | 1 | 0 | 0 | 0 | 0 |
| S27.71 | Multiple injuries to intrathoracic organs, with intrathoracic injury | Mild | 0 | 0 | 0 | 1 | 0 | 0 | 0 | 0 |
| S27.8 | Injury to other intrathoracic organs, unspecified | Non severe | 0 | 0 | 0 | 1 | 0 | 0 | 0 | 0 |
| S27.80 | Injury to other intrathoracic organs, unspecified, without intrathoracic injury | Mild | 0 | 0 | 0 | 1 | 0 | 0 | 0 | 0 |
| S27.81 | Injury to other intrathoracic organs, unspecified, with intrathoracic injury | Mild | 0 | 0 | 0 | 1 | 0 | 0 | 0 | 0 |
| S28.0 | Crushing injury to the thorax | Severe | 0 | 0 | 0 | 1 | 0 | 0 | 0 | 0 |
| S28.1 | Amputation of part of the thorax | Severe | 0 | 0 | 0 | 1 | 0 | 0 | 0 | 0 |
| S29.0 | Injury to muscles and tendons of the thorax | Non severe | 0 | 0 | 0 | 1 | 0 | 0 | 0 | 0 |
| S29.7 | Multiple injuries to the thorax | Mild | 0 | 0 | 0 | 1 | 0 | 0 | 0 | 0 |
| S29.8 | Injury to unspecified part of the thorax | Non severe | 0 | 0 | 0 | 1 | 0 | 0 | 0 | 0 |
| S30.0 | Contusion of the lower back, pelvis | Non severe | 0 | 0 | 0 | 0 | 1 | 0 | 0 | 0 |
| S30.1 | Contusion of the abdominal wall | Non severe | 0 | 0 | 0 | 0 | 1 | 0 | 0 | 0 |
| S30.2 | Contusion of external genital organs | Non severe | 0 | 0 | 0 | 0 | 1 | 0 | 0 | 0 |
| S30.7 | Superficial multiple injuries to the abdomen, lower back, pelvis | Non severe | 0 | 0 | 1 | 0 | 0 | 0 | 0 | 0 |
| S30.8 | Superficial injury to unspecified part of the abdomen, lower back, pelvis | Non severe | 0 | 0 | 1 | 0 | 0 | 0 | 0 | 0 |
| S31.0 | Open wound of the lower back, pelvis | Non severe | 0 | 0 | 0 | 0 | 1 | 0 | 0 | 0 |
| S31.1 | Open wound of the abdominal wall | Non severe | 0 | 0 | 0 | 0 | 1 | 0 | 0 | 0 |
| S31.2 | Open wound of the penis | Non severe | 0 | 0 | 0 | 0 | 1 | 0 | 0 | 0 |
| S31.3 | Open wound of the scrotum and testicles | Non severe | 0 | 0 | 0 | 0 | 1 | 0 | 0 | 0 |
| S31.4 | Open wound of the vagina and vulva | Non severe | 0 | 0 | 0 | 0 | 1 | 0 | 0 | 0 |
| S31.5 | Open wound of external genital organs, not coded elsewhere or Without further information | Non severe | 0 | 0 | 0 | 0 | 1 | 0 | 0 | 0 |
| S31.7 | Multiple open wounds of the abdomen, lower back, pelvis | Mild | 0 | 0 | 0 | 0 | 1 | 0 | 0 | 0 |
| S31.8 | Open wound of parts not coded elsewhere or Without further information of the abdomen | Non severe | 0 | 0 | 0 | 0 | 1 | 0 | 0 | 0 |
| S32.00 | Closed fracture of a lumbar vertebra | Non severe | 0 | 0 | 0 | 0 | 1 | 0 | 0 | 0 |
| S32.01 | Open fracture of a lumbar vertebra | Mild | 0 | 0 | 0 | 0 | 1 | 0 | 0 | 0 |
| S32.10 | Closed fracture of the sacrum | Mild | 0 | 0 | 0 | 0 | 1 | 0 | 0 | 0 |
| S32.11 | Open fracture of the sacrum | Mild | 0 | 0 | 0 | 0 | 1 | 0 | 0 | 0 |
| S32.20 | Closed fracture of the coccyx | Mild | 0 | 0 | 0 | 0 | 1 | 0 | 0 | 0 |
| S32.21 | Open fracture of the coccyx | Mild | 0 | 0 | 0 | 0 | 1 | 0 | 0 | 0 |
| S32.30 | Closed fracture of the ilium | Mild | 0 | 0 | 0 | 0 | 1 | 0 | 0 | 0 |
| S32.31 | Open fracture of the ilium | Mild | 0 | 0 | 0 | 0 | 1 | 0 | 0 | 0 |
| S32.40 | Closed fracture of the acetabulum | Mild | 0 | 0 | 0 | 0 | 1 | 0 | 0 | 0 |
| S32.41 | Open fracture of the acetabulum | Mild | 0 | 0 | 0 | 0 | 1 | 0 | 0 | 0 |
| S32.50 | Closed fracture of the pubis | Mild | 0 | 0 | 0 | 0 | 1 | 0 | 0 | 0 |
| S32.51 | Open fracture of the pubis | Mild | 0 | 0 | 0 | 0 | 1 | 0 | 0 | 0 |
| S32.70 | Closed multiple fractures of the lumbar spine and pelvis | Severe | 0 | 0 | 0 | 0 | 1 | 0 | 0 | 0 |
| S32.71 | Open multiple fractures of the lumbar spine and pelvis | Severe | 0 | 0 | 0 | 0 | 1 | 0 | 0 | 0 |
| S32.80 | Closed fracture of parts not coded elsewhere or Without further information of the lumbar spine and pelvis | Non severe | 0 | 0 | 0 | 0 | 1 | 0 | 0 | 0 |
| S32.81 | Open fracture of parts not coded elsewhere or Without further information of the lumbar spine and pelvis | Mild | 0 | 0 | 0 | 0 | 1 | 0 | 0 | 0 |
| S33.0 | Trauma rupture of a lumbar intervertebral disc | Non severe | 0 | 0 | 0 | 0 | 1 | 0 | 0 | 0 |
| S33.1 | Luxation of a lumbar vertebra | Mild | 0 | 0 | 0 | 0 | 1 | 0 | 0 | 0 |
| S33.2 | Luxation of the sacroiliac and sacrococcygeal joints | Mild | 0 | 0 | 0 | 0 | 1 | 0 | 0 | 0 |
| S33.3 | Luxation of parts not coded elsewhere or Without further information of the lumbar spine and pelvis | Mild | 0 | 0 | 0 | 0 | 1 | 0 | 0 | 0 |
| S33.4 | Trauma rupture of the pubic symphysis | Severe | 0 | 0 | 0 | 0 | 1 | 0 | 0 | 0 |
| S33.5 | Sprain of the lumbar spine | Mild | 0 | 0 | 0 | 0 | 1 | 0 | 0 | 0 |
| S33.6 | Sprain of the sacroiliac joint | Non severe | 0 | 0 | 0 | 0 | 1 | 0 | 0 | 0 |
| S33.7 | Sprain of parts not coded elsewhere or Without further information of the lumbar spine and pelvis | Mild | 0 | 0 | 0 | 0 | 1 | 0 | 0 | 0 |
| S34.0 | Concussion and edema of the lumbar spinal cord | Mild | 0 | 0 | 0 | 0 | 1 | 0 | 0 | 0 |
| S34.1 | Trauma to the lumbar spinal cord, not coded elsewhere | Mild | 0 | 0 | 0 | 0 | 1 | 0 | 0 | 0 |
| S34.2 | Trauma to the nerve root of the lumbar and sacral spine | Mild | 0 | 0 | 0 | 0 | 1 | 0 | 0 | 0 |
| S34.3 | Trauma to the cauda equina | Mild | 0 | 0 | 0 | 0 | 1 | 0 | 0 | 0 |
| S34.4 | Trauma to the lumbosacral plexus | Mild | 0 | 0 | 0 | 0 | 1 | 0 | 0 | 0 |
| S34.5 | Trauma to the sympathetic nerves of the lumbar, sacral, and pelvic regions | Non severe | 0 | 0 | 0 | 0 | 1 | 0 | 0 | 0 |
| S34.6 | Trauma to peripheral nerves of the abdomen, lower back, pelvis | Non severe | 0 | 0 | 0 | 0 | 1 | 0 | 0 | 0 |
| S34.8 | Trauma to nerves not coded elsewhere or Without further information in the abdomen, lower back, pelvis | Non severe | 0 | 0 | 0 | 0 | 1 | 0 | 0 | 0 |
| S35.0 | Trauma to the abdominal aorta | Severe | 0 | 0 | 0 | 0 | 1 | 0 | 0 | 0 |
| S35.1 | Trauma to the inferior vena cava | Severe | 0 | 0 | 0 | 0 | 1 | 0 | 0 | 0 |
| S35.2 | Trauma to the celiac or mesenteric artery | Severe | 0 | 0 | 0 | 0 | 1 | 0 | 0 | 0 |
| S35.3 | Trauma to the portal or splenic vein | Severe | 0 | 0 | 0 | 0 | 1 | 0 | 0 | 0 |
| S35.4 | Trauma to the renal blood vessels | Severe | 0 | 0 | 0 | 0 | 1 | 0 | 0 | 0 |
| S35.5 | Trauma to the iliac blood vessels | Severe | 0 | 0 | 0 | 0 | 1 | 0 | 0 | 0 |
| S35.7 | Trauma to multiple blood vessels in the abdomen, lower back, pelvis | Severe | 0 | 0 | 0 | 0 | 1 | 0 | 0 | 0 |
| S35.8 | Trauma to other blood vessels in the abdomen, lower back, pelvis | Non severe | 0 | 0 | 0 | 0 | 1 | 0 | 0 | 0 |
| S35.9 | Trauma to blood vessels, unspecified, in the abdomen, lower back, pelvis | Non severe | 0 | 0 | 0 | 0 | 1 | 0 | 0 | 0 |
| S36.0 | Trauma to the spleen | Mild | 0 | 0 | 0 | 0 | 1 | 0 | 0 | 0 |
| S36.00 | Trauma to the spleen, without intra-abdominal injury | Mild | 0 | 0 | 0 | 0 | 1 | 0 | 0 | 0 |
| S36.01 | Trauma to the spleen, with intra-abdominal injury | Severe | 0 | 0 | 0 | 0 | 1 | 0 | 0 | 0 |
| S36.1 | Trauma to the liver and gallbladder | Mild | 0 | 0 | 0 | 0 | 1 | 0 | 0 | 0 |
| S36.10 | Trauma to the liver and gallbladder, without intra-abdominal injury | Mild | 0 | 0 | 0 | 0 | 1 | 0 | 0 | 0 |
| S36.11 | Trauma to the liver and gallbladder, with intra-abdominal injury | Severe | 0 | 0 | 0 | 0 | 1 | 0 | 0 | 0 |
| S36.2 | Trauma to the pancreas | Mild | 0 | 0 | 0 | 0 | 1 | 0 | 0 | 0 |
| S36.20 | Trauma to the pancreas, without intra-abdominal injury | Mild | 0 | 0 | 0 | 0 | 1 | 0 | 0 | 0 |
| S36.21 | Trauma to the pancreas, with intra-abdominal injury | Severe | 0 | 0 | 0 | 0 | 1 | 0 | 0 | 0 |
| S36.3 | Trauma to the stomach | Mild | 0 | 0 | 0 | 0 | 1 | 0 | 0 | 0 |
| S36.30 | Trauma to the stomach, without intra-abdominal injury | Mild | 0 | 0 | 0 | 0 | 1 | 0 | 0 | 0 |
| S36.31 | Trauma to the stomach, with intra-abdominal injury | Severe | 0 | 0 | 0 | 0 | 1 | 0 | 0 | 0 |
| S36.4 | Trauma to the small intestine | Mild | 0 | 0 | 0 | 0 | 1 | 0 | 0 | 0 |
| S36.40 | Trauma to the small intestine, without intra-abdominal injury | Mild | 0 | 0 | 0 | 0 | 1 | 0 | 0 | 0 |
| S36.41 | Trauma to the small intestine, with intra-abdominal injury | Severe | 0 | 0 | 0 | 0 | 1 | 0 | 0 | 0 |
| S36.5 | Trauma to the colon | Mild | 0 | 0 | 0 | 0 | 1 | 0 | 0 | 0 |
| S36.50 | Trauma to the colon, without intra-abdominal injury | Mild | 0 | 0 | 0 | 0 | 1 | 0 | 0 | 0 |
| S36.51 | Trauma to the colon, with intra-abdominal injury | Severe | 0 | 0 | 0 | 0 | 1 | 0 | 0 | 0 |
| S36.6 | Trauma to the rectum | Mild | 0 | 0 | 0 | 0 | 1 | 0 | 0 | 0 |
| S36.60 | Trauma to the rectum, without intra-abdominal injury | Mild | 0 | 0 | 0 | 0 | 1 | 0 | 0 | 0 |
| S36.61 | Trauma to the rectum, with intra-abdominal injury | Severe | 0 | 0 | 0 | 0 | 1 | 0 | 0 | 0 |
| S36.7 | Trauma to multiple intra-abdominal organs | Severe | 0 | 0 | 0 | 0 | 1 | 0 | 0 | 0 |
| S36.70 | Trauma to multiple intra-abdominal organs, without intra-abdominal injury | Severe | 0 | 0 | 0 | 0 | 1 | 0 | 0 | 0 |
| S36.71 | Trauma to multiple intra-abdominal organs, with intra-abdominal injury | Severe | 0 | 0 | 0 | 0 | 1 | 0 | 0 | 0 |
| S36.8 | Trauma to other intra-abdominal organs | Mild | 0 | 0 | 0 | 0 | 1 | 0 | 0 | 0 |
| S36.80 | Trauma to other intra-abdominal organs, without intra-abdominal injury | Mild | 0 | 0 | 0 | 0 | 1 | 0 | 0 | 0 |
| S36.81 | Trauma to other intra-abdominal organs, with intra-abdominal injury | Mild | 0 | 0 | 0 | 0 | 1 | 0 | 0 | 0 |
| S37.0 | Trauma to the kidney | Mild | 0 | 0 | 0 | 0 | 1 | 0 | 0 | 0 |
| S37.00 | Trauma to the kidney, without intra-abdominal injury | Mild | 0 | 0 | 0 | 0 | 1 | 0 | 0 | 0 |
| S37.01 | Trauma to the kidney, with intra-abdominal injury | Severe | 0 | 0 | 0 | 0 | 1 | 0 | 0 | 0 |
| S37.1 | Trauma to the ureter | Mild | 0 | 0 | 0 | 0 | 1 | 0 | 0 | 0 |
| S37.10 | Trauma to the ureter, without intra-abdominal injury | Mild | 0 | 0 | 0 | 0 | 1 | 0 | 0 | 0 |
| S37.11 | Trauma to the ureter, with intra-abdominal injury | Mild | 0 | 0 | 0 | 0 | 1 | 0 | 0 | 0 |
| S37.2 | Trauma to the bladder | Mild | 0 | 0 | 0 | 0 | 1 | 0 | 0 | 0 |
| S37.20 | Trauma to the bladder, without intra-pelvic injury | Mild | 0 | 0 | 0 | 0 | 1 | 0 | 0 | 0 |
| S37.21 | Trauma to the bladder, with intra-pelvic injury | Mild | 0 | 0 | 0 | 0 | 1 | 0 | 0 | 0 |
| S37.3 | Trauma to the urethra | Mild | 0 | 0 | 0 | 0 | 1 | 0 | 0 | 0 |
| S37.30 | Trauma to the urethra, without intra-pelvic injury | Mild | 0 | 0 | 0 | 0 | 1 | 0 | 0 | 0 |
| S37.31 | Trauma to the urethra, with intra-pelvic injury | Mild | 0 | 0 | 0 | 0 | 1 | 0 | 0 | 0 |
| S37.4 | Trauma to the ovary | Mild | 0 | 0 | 0 | 0 | 1 | 0 | 0 | 0 |
| S37.40 | Trauma to the ovary, without intra-pelvic injury | Mild | 0 | 0 | 0 | 0 | 1 | 0 | 0 | 0 |
| S37.41 | Trauma to the ovary, with intra-pelvic injury | Mild | 0 | 0 | 0 | 0 | 1 | 0 | 0 | 0 |
| S37.5 | Trauma to the fallopian tube | Mild | 0 | 0 | 0 | 0 | 1 | 0 | 0 | 0 |
| S37.50 | Trauma to the fallopian tube, without intra-pelvic injury | Mild | 0 | 0 | 0 | 0 | 1 | 0 | 0 | 0 |
| S37.51 | Trauma to the fallopian tube, with intra-pelvic injury | Mild | 0 | 0 | 0 | 0 | 1 | 0 | 0 | 0 |
| S37.6 | Trauma to the uterus | Mild | 0 | 0 | 0 | 0 | 1 | 0 | 0 | 0 |
| S37.60 | Trauma to the uterus, without intra-pelvic injury | Mild | 0 | 0 | 0 | 0 | 1 | 0 | 0 | 0 |
| S37.61 | Trauma to the uterus, with intra-pelvic injury | Mild | 0 | 0 | 0 | 0 | 1 | 0 | 0 | 0 |
| S37.7 | Trauma to multiple pelvic, urinary organs | Severe | 0 | 0 | 0 | 0 | 1 | 0 | 0 | 0 |
| S37.70 | Trauma to multiple pelvic, urinary organs, without intra-pelvic injury | Severe | 0 | 0 | 0 | 0 | 1 | 0 | 0 | 0 |
| S37.71 | Trauma to multiple pelvic, urinary organs, with intra-pelvic injury | Severe | 0 | 0 | 0 | 0 | 1 | 0 | 0 | 0 |
| S37.800 | Trauma to the adrenal gland, without intra-abdominal injury | Mild | 0 | 0 | 0 | 0 | 1 | 0 | 0 | 0 |
| S37.808 | Trauma to pelvic organs, urinary, unspecified, except adrenal gland, without injury | Mild | 0 | 0 | 0 | 0 | 1 | 0 | 0 | 0 |
| S37.810 | Trauma to the adrenal gland, with intra-abdominal injury | Severe | 0 | 0 | 0 | 0 | 1 | 0 | 0 | 0 |
| S37.818 | Trauma to pelvic organs, urinary, unspecified, except adrenal gland, with injury | Mild | 0 | 0 | 0 | 0 | 1 | 0 | 0 | 0 |
| S38.0 | Crushing injury to external genital organs | Mild | 0 | 0 | 0 | 0 | 1 | 0 | 0 | 0 |
| S38.10 | Crushing injury to the buttocks | Mild | 0 | 0 | 0 | 0 | 1 | 0 | 0 | 0 |
| S38.18 | Crushing injury to parts not coded elsewhere of the abdomen, lower back, pelvis (except buttocks) | Mild | 0 | 0 | 0 | 0 | 1 | 0 | 0 | 0 |
| S38.2 | Amputated trauma to external genital organs | Mild | 0 | 0 | 0 | 0 | 1 | 0 | 0 | 0 |
| S38.3 | Amputated trauma to parts not coded elsewhere or Without further information of the abdomen, lower back, pelvis | Mild | 0 | 0 | 0 | 0 | 1 | 0 | 0 | 0 |
| S39.0 | Trauma to abdominal tendons | Non severe | 0 | 0 | 0 | 0 | 1 | 0 | 0 | 0 |
| S39.6 | Trauma to intra-abdominal organs with pelvic organs | Severe | 0 | 0 | 0 | 0 | 1 | 0 | 0 | 0 |
| S39.7 | Multiple trauma to the abdomen, lower back, pelvis, not coded elsewhere | Severe | 0 | 0 | 0 | 0 | 1 | 0 | 0 | 0 |
| S39.8 | Precise trauma to the abdomen, lower back, pelvis, not coded elsewhere | Mild | 0 | 0 | 0 | 0 | 1 | 0 | 0 | 0 |
| S40.0 | Contusion of the shoulder, arm | Non severe | 0 | 0 | 0 | 0 | 0 | 1 | 0 | 0 |
| S40.7 | Superficial trauma to multiple parts of the shoulder, arm | Non severe | 0 | 0 | 0 | 0 | 0 | 0 | 0 | 0 |
| S40.8 | Superficial trauma to parts of the shoulder, arm, unspecified | Non severe | 0 | 0 | 0 | 0 | 0 | 0 | 0 | 0 |
| S40.9 | Superficial trauma to parts of the shoulder, arm, Without further information | Non severe | 0 | 0 | 0 | 0 | 0 | 0 | 0 | 0 |
| S41.0 | Open wound of the shoulder | Non severe | 0 | 0 | 0 | 0 | 0 | 1 | 0 | 0 |
| S41.1 | Open wound of the arm | Non severe | 0 | 0 | 0 | 0 | 0 | 1 | 0 | 0 |
| S41.7 | Multiple open wounds of the shoulder, arm | Non severe | 0 | 0 | 0 | 0 | 0 | 1 | 0 | 0 |
| S41.8 | Open wound of unspecified parts of the scapular belt | Non severe | 0 | 0 | 0 | 0 | 0 | 1 | 0 | 0 |
| S42.00 | Closed fracture of the clavicle | Non severe | 0 | 0 | 0 | 0 | 0 | 1 | 0 | 0 |
| S42.01 | Open fracture of the clavicle | Non severe | 0 | 0 | 0 | 0 | 0 | 1 | 0 | 0 |
| S42.10 | Closed fracture of the scapula | Non severe | 0 | 0 | 0 | 0 | 0 | 1 | 0 | 0 |
| S42.11 | Open fracture of the scapula | Non severe | 0 | 0 | 0 | 0 | 0 | 1 | 0 | 0 |
| S42.20 | Closed fracture of the proximal humerus | Non severe | 0 | 0 | 0 | 0 | 0 | 1 | 0 | 0 |
| S42.21 | Fracture open of the upper extremity of the humerus | Mild | 0 | 0 | 0 | 0 | 0 | 1 | 0 | 0 |
| S42.30 | Closed fracture of the diaphysis of the humerus | Mild | 0 | 0 | 0 | 0 | 0 | 1 | 0 | 0 |
| S42.31 | Open fracture of the diaphysis of the humerus | Mild | 0 | 0 | 0 | 0 | 0 | 1 | 0 | 0 |
| S42.40 | Closed fracture of the lower extremity of the humerus | Mild | 0 | 0 | 0 | 0 | 0 | 1 | 0 | 0 |
| S42.41 | Open fracture of the lower extremity of the humerus | Mild | 0 | 0 | 0 | 0 | 0 | 1 | 0 | 0 |
| S42.70 | Multiple closed fractures of the clavicle, scapula, and humerus | Mild | 0 | 0 | 0 | 0 | 0 | 1 | 0 | 0 |
| S42.71 | Multiple open fractures of the clavicle, scapula, and humerus | Mild | 0 | 0 | 0 | 0 | 0 | 1 | 0 | 0 |
| S42.80 | Closed fracture of other parts of the shoulder, arm | Mild | 0 | 0 | 0 | 0 | 0 | 1 | 0 | 0 |
| S42.81 | Open fracture of other parts of the shoulder, arm | Mild | 0 | 0 | 0 | 0 | 0 | 1 | 0 | 0 |
| S42.90 | Closed fracture of the scapular girdle, part Without further information | Mild | 0 | 0 | 0 | 0 | 0 | 1 | 0 | 0 |
| S42.91 | Open fracture of the scapular girdle, part Without further information | Mild | 0 | 0 | 0 | 0 | 0 | 1 | 0 | 0 |
| S43.0 | Luxation of the shoulder joint | Mild | 0 | 0 | 0 | 0 | 0 | 1 | 0 | 0 |
| S43.1 | Luxation of the acromioclavicular joint | Mild | 0 | 0 | 0 | 0 | 0 | 1 | 0 | 0 |
| S43.2 | Luxation of the sternoclavicular joint | Mild | 0 | 0 | 0 | 0 | 0 | 1 | 0 | 0 |
| S43.3 | Luxation of non-classifiable or Without further information parts of the scapular girdle | Mild | 0 | 0 | 0 | 0 | 0 | 1 | 0 | 0 |
| S43.4 | Sprain of the shoulder joint | Mild | 0 | 0 | 0 | 0 | 0 | 1 | 0 | 0 |
| S43.5 | Sprain of the acromioclavicular joint | Mild | 0 | 0 | 0 | 0 | 0 | 1 | 0 | 0 |
| S43.6 | Sprain of the sternoclavicular joint | Mild | 0 | 0 | 0 | 0 | 0 | 1 | 0 | 0 |
| S43.7 | Sprain of non-classifiable or Without further information parts of the scapular girdle | Mild | 0 | 0 | 0 | 0 | 0 | 1 | 0 | 0 |
| S44.0 | Traumatic lesion of the ulnar nerve at the arm level | Mild | 0 | 0 | 0 | 0 | 0 | 1 | 0 | 0 |
| S44.1 | Traumatic lesion of the median nerve at the arm level | Mild | 0 | 0 | 0 | 0 | 0 | 1 | 0 | 0 |
| S44.2 | Traumatic lesion of the radial nerve at the arm level | Mild | 0 | 0 | 0 | 0 | 0 | 1 | 0 | 0 |
| S44.3 | Traumatic lesion of the axillary nerve | Mild | 0 | 0 | 0 | 0 | 0 | 1 | 0 | 0 |
| S44.4 | Traumatic lesion of a musculocutaneous nerve | Mild | 0 | 0 | 0 | 0 | 0 | 1 | 0 | 0 |
| S44.5 | Traumatic lesion of a cutaneous sensory nerve at the shoulder, arm | Mild | 0 | 0 | 0 | 0 | 0 | 1 | 0 | 0 |
| S44.7 | Traumatic lesion of multiple nerves at the shoulder, arm | Mild | 0 | 0 | 0 | 0 | 0 | 1 | 0 | 0 |
| S44.8 | Traumatic lesion of other nerves at the shoulder, arm | Mild | 0 | 0 | 0 | 0 | 0 | 1 | 0 | 0 |
| S44.9 | Traumatic lesion of a Without further information nerve at the shoulder, arm | Mild | 0 | 0 | 0 | 0 | 0 | 1 | 0 | 0 |
| S45.0 | Traumatic lesion of the axillary artery | Severe | 0 | 0 | 0 | 0 | 0 | 1 | 0 | 0 |
| S45.1 | Traumatic lesion of the brachial artery | Severe | 0 | 0 | 0 | 0 | 0 | 1 | 0 | 0 |
| S45.2 | Traumatic lesion of the axillary and brachial veins | Mild | 0 | 0 | 0 | 0 | 0 | 1 | 0 | 0 |
| S45.3 | Traumatic lesion of a superficial vein at the shoulder, arm | Mild | 0 | 0 | 0 | 0 | 0 | 1 | 0 | 0 |
| S45.7 | Traumatic lesion of multiple veins at the shoulder, arm | Mild | 0 | 0 | 0 | 0 | 0 | 1 | 0 | 0 |
| S45.8 | Traumatic lesion of other veins at the shoulder, arm | Mild | 0 | 0 | 0 | 0 | 0 | 1 | 0 | 0 |
| S45.9 | Traumatic lesion of a Without further information vein at the shoulder, arm | Mild | 0 | 0 | 0 | 0 | 0 | 1 | 0 | 0 |
| S46.0 | Traumatic lesion of the rotator cuff muscles and tendons | Mild | 0 | 0 | 0 | 0 | 0 | 1 | 0 | 0 |
| S46.1 | Traumatic lesion of the long biceps tendon | Mild | 0 | 0 | 0 | 0 | 0 | 1 | 0 | 0 |
| S46.2 | Traumatic lesion of tendons of other parts of the biceps | Mild | 0 | 0 | 0 | 0 | 0 | 1 | 0 | 0 |
| S46.3 | Traumatic lesion of the triceps tendon | Mild | 0 | 0 | 0 | 0 | 0 | 1 | 0 | 0 |
| S46.7 | Traumatic lesion of multiple tendons at the shoulder, arm | Mild | 0 | 0 | 0 | 0 | 0 | 1 | 0 | 0 |
| S46.8 | Traumatic lesion of other tendons at the shoulder, arm | Mild | 0 | 0 | 0 | 0 | 0 | 1 | 0 | 0 |
| S46.9 | Traumatic lesion of muscle and tendon Without further information at the shoulder, arm | Mild | 0 | 0 | 0 | 0 | 0 | 1 | 0 | 0 |
| S47.0 | Crushing injury to the scapular region | Mild | 0 | 0 | 0 | 0 | 0 | 1 | 0 | 0 |
| S47.8 | Crushing injury to non-classifiable or Without further information parts of the shoulder, arm, except scapular region | Mild | 0 | 0 | 0 | 0 | 0 | 1 | 0 | 0 |
| S48.0 | Traumatic amputation of the shoulder joint | Severe | 0 | 0 | 0 | 0 | 0 | 1 | 0 | 0 |
| S48.1 | Traumatic amputation between the shoulder and elbow | Severe | 0 | 0 | 0 | 0 | 0 | 1 | 0 | 0 |
| S48.9 | Traumatic amputation of the shoulder, arm, Without further information level | Severe | 0 | 0 | 0 | 0 | 0 | 1 | 0 | 0 |
| S49.7 | Traumatic lesion of multiple parts of the shoulder, arm | Mild | 0 | 0 | 0 | 0 | 0 | 1 | 0 | 0 |
| S49.8 | Traumatic lesion of unspecified parts of the shoulder, arm | Mild | 0 | 0 | 0 | 0 | 0 | 1 | 0 | 0 |
| S50.0 | Contusion of the elbow | Mild | 0 | 0 | 0 | 0 | 0 | 1 | 0 | 0 |
| S50.1 | Contusion of non-classifiable or Without further information parts of the forearm | Mild | 0 | 0 | 0 | 0 | 0 | 1 | 0 | 0 |
| S50.7 | Superficial traumatic lesions of multiple forearm parts | Mild | 0 | 0 | 1 | 0 | 0 | 0 | 0 | 0 |
| S50.8 | Superficial traumatic lesion of non-classifiable forearm parts | Mild | 0 | 0 | 1 | 0 | 0 | 0 | 0 | 0 |
| S50.9 | Superficial traumatic lesion of Without further information parts of the forearm | Mild | 0 | 0 | 1 | 0 | 0 | 0 | 0 | 0 |
| S51.0 | Open wound of the elbow | Mild | 0 | 0 | 0 | 0 | 0 | 1 | 0 | 0 |
| S51.7 | Multiple open wounds of the forearm | Mild | 0 | 0 | 0 | 0 | 0 | 1 | 0 | 0 |
| S51.8 | Open wound of other parts of the forearm | Mild | 0 | 0 | 0 | 0 | 0 | 1 | 0 | 0 |
| S51.9 | Open wound of the forearm, Without further information part | Mild | 0 | 0 | 0 | 0 | 0 | 1 | 0 | 0 |
| S52.00 | Closed fracture of the upper part of the ulna | Mild | 0 | 0 | 0 | 0 | 0 | 1 | 0 | 0 |
| S52.01 | Open fracture of the upper part of the ulna | Mild | 0 | 0 | 0 | 0 | 0 | 1 | 0 | 0 |
| S52.10 | Closed fracture of the upper extremity of the radius | Mild | 0 | 0 | 0 | 0 | 0 | 1 | 0 | 0 |
| S52.11 | Open fracture of the upper extremity of the radius | Mild | 0 | 0 | 0 | 0 | 0 | 1 | 0 | 0 |
| S52.20 | Closed fracture of the diaphysis of the ulna | Mild | 0 | 0 | 0 | 0 | 0 | 1 | 0 | 0 |
| S52.21 | Open fracture of the diaphysis of the ulna | Mild | 0 | 0 | 0 | 0 | 0 | 1 | 0 | 0 |
| S52.30 | Closed fracture of the diaphysis of the radius | Mild | 0 | 0 | 0 | 0 | 0 | 1 | 0 | 0 |
| S52.31 | Open fracture of the diaphysis of the radius | Mild | 0 | 0 | 0 | 0 | 0 | 1 | 0 | 0 |
| S52.40 | Closed fractures of both the diaphyses, ulnar and radial | Mild | 0 | 0 | 0 | 0 | 0 | 1 | 0 | 0 |
| S52.41 | Open fractures of both the diaphyses, ulnar and radial | Mild | 0 | 0 | 0 | 0 | 0 | 1 | 0 | 0 |
| S52.50 | Closed fracture of the lower extremity of the radius | Mild | 0 | 0 | 0 | 0 | 0 | 1 | 0 | 0 |
| S52.51 | Open fracture of the lower extremity of the radius | Mild | 0 | 0 | 0 | 0 | 0 | 1 | 0 | 0 |
| S52.60 | Closed fracture of the lower extremity of the ulna and radius | Mild | 0 | 0 | 0 | 0 | 0 | 1 | 0 | 0 |
| S52.61 | Open fracture of the lower extremity of the ulna and radius | Mild | 0 | 0 | 0 | 0 | 0 | 1 | 0 | 0 |
| S52.70 | Multiple closed fractures of the forearm | Mild | 0 | 0 | 0 | 0 | 0 | 1 | 0 | 0 |
| S52.71 | Multiple open fractures of the forearm | Mild | 0 | 0 | 0 | 0 | 0 | 1 | 0 | 0 |
| S52.80 | Closed fracture of other parts of the forearm | Mild | 0 | 0 | 0 | 0 | 0 | 1 | 0 | 0 |
| S52.81 | Open fracture of other parts of the forearm | Mild | 0 | 0 | 0 | 0 | 0 | 1 | 0 | 0 |
| S52.90 | Closed fracture of the forearm, Without further information part | Mild | 0 | 0 | 0 | 0 | 0 | 1 | 0 | 0 |
| S52.91 | Open fracture of the forearm, Without further information part | Mild | 0 | 0 | 0 | 0 | 0 | 1 | 0 | 0 |
| S53.0 | Luxation of the radial head | Mild | 0 | 0 | 0 | 0 | 0 | 1 | 0 | 0 |
| S53.1 | Luxation of the elbow, Without further information | Mild | 0 | 0 | 0 | 0 | 0 | 1 | 0 | 0 |
| S53.2 | Rupture of the lateral external ligament of the elbow | Mild | 0 | 0 | 0 | 0 | 0 | 1 | 0 | 0 |
| S53.3 | Rupture of the lateral internal ligament of the elbow | Mild | 0 | 0 | 0 | 0 | 0 | 1 | 0 | 0 |
| S53.4 | Sprain of the elbow | Mild | 0 | 0 | 0 | 0 | 0 | 1 | 0 | 0 |
| S54.0 | Traumatic lesion of the ulnar nerve at the forearm or Without further information | Mild | 0 | 0 | 0 | 0 | 0 | 1 | 0 | 0 |
| S54.1 | Traumatic lesion of the median nerve at the forearm or Without further information | Mild | 0 | 0 | 0 | 0 | 0 | 1 | 0 | 0 |
| S54.2 | Traumatic lesion of the radial nerve at the forearm or Without further information | Mild | 0 | 0 | 0 | 0 | 0 | 1 | 0 | 0 |
| S54.3 | Lesion of a cutaneous sensory nerve due to trauma | Non severe | 0 | 0 | 0 | 0 | 0 | 1 | 0 | 0 |
| S54.7 | Lesion of multiple nerves at a cervical level, intermédiaire | Non severe | 0 | 0 | 0 | 0 | 0 | 1 | 0 | 0 |
| S54.8 | Lesion of other nerves at a cervical level, intermédiaire | Non severe | 0 | 0 | 0 | 0 | 0 | 1 | 0 | 0 |
| S54.9 | Lesion of a nerve at a spinal level, intermédiaire | Non severe | 0 | 0 | 0 | 0 | 0 | 1 | 0 | 0 |
| S55.0 | Lesion of the cubital artery at a cervical level, intermédiaire | Mild | 0 | 0 | 0 | 0 | 0 | 1 | 0 | 0 |
| S55.1 | Lesion of the radial artery at a cervical level, intermédiaire | Mild | 0 | 0 | 0 | 0 | 0 | 1 | 0 | 0 |
| S55.2 | Lesion of a vein at a cervical level, intermédiaire | Non severe | 0 | 0 | 0 | 0 | 0 | 1 | 0 | 0 |
| S55.7 | Lesion of multiple vessels and blood at a cervical level, intermédiaire | Mild | 0 | 0 | 0 | 0 | 0 | 1 | 0 | 0 |
| S55.8 | Lesion of other vessels and blood at a cervical level, intermédiaire | Non severe | 0 | 0 | 0 | 0 | 0 | 1 | 0 | 0 |
| S55.9 | Lesion of a blood vessel at a spinal level, intermédiaire | Non severe | 0 | 0 | 0 | 0 | 0 | 1 | 0 | 0 |
| S56.0 | Lesion of the flexor tendon of the thumb at a cervical level, intermédiaire | Non severe | 0 | 0 | 0 | 0 | 0 | 1 | 0 | 0 |
| S56.1 | Lesion of the flexor tendons of other fingers at a cervical level, intermédiaire | Non severe | 0 | 0 | 0 | 0 | 0 | 1 | 0 | 0 |
| S56.2 | Lesion of another flexor tendon at a cervical level, intermédiaire | Non severe | 0 | 0 | 0 | 0 | 0 | 1 | 0 | 0 |
| S56.3 | Lesion of the extensor and abductor tendons of the thumb at a cervical level, intermédiaire | Non severe | 0 | 0 | 0 | 0 | 0 | 1 | 0 | 0 |
| S56.4 | Lesion of the extensor tendons of other fingers at a cervical level, intermédiaire | Non severe | 0 | 0 | 0 | 0 | 0 | 1 | 0 | 0 |
| S56.5 | Lesion of another extensor tendon at a cervical level, intermédiaire | Non severe | 0 | 0 | 0 | 0 | 0 | 1 | 0 | 0 |
| S56.7 | Lesion of multiple tendons at a cervical level, intermédiaire | Non severe | 0 | 0 | 0 | 0 | 0 | 1 | 0 | 0 |
| S56.8 | Lesion of tendons from the neck or spinal level, intermédiaire | Non severe | 0 | 0 | 0 | 0 | 0 | 1 | 0 | 0 |
| S57.0 | Crush injury of the elbow | Mild | 0 | 0 | 0 | 0 | 0 | 1 | 0 | 0 |
| S57.8 | Crush injury of other parts of the upper body | Mild | 0 | 0 | 0 | 0 | 0 | 1 | 0 | 0 |
| S57.9 | Crush injury of the upper body, unspecified part | Mild | 0 | 0 | 0 | 0 | 0 | 1 | 0 | 0 |
| S58.0 | Amputation trauma, elbow level | Severe | 0 | 0 | 0 | 0 | 0 | 1 | 0 | 0 |
| S58.1 | Amputation trauma, between the elbow and wrist | Severe | 0 | 0 | 0 | 0 | 0 | 1 | 0 | 0 |
| S58.9 | Amputation trauma, intermédiaire level, Without further information | Severe | 0 | 0 | 0 | 0 | 0 | 1 | 0 | 0 |
| S59.7 | Lesion trauma, multiple intermédiaire | Non severe | 0 | 0 | 0 | 0 | 0 | 1 | 0 | 0 |
| S59.8 | Lesion trauma, specific intermédiaire, not coded elsewhere | Non severe | 0 | 0 | 0 | 0 | 0 | 1 | 0 | 0 |
| S60.0 | Contusion of finger without nail lesion | Non severe | 0 | 0 | 0 | 0 | 0 | 1 | 0 | 0 |
| S60.1 | Contusion of finger with nail lesion | Non severe | 0 | 0 | 0 | 0 | 0 | 1 | 0 | 0 |
| S60.2 | Contusion of other parts of wrist, hand | Non severe | 0 | 0 | 0 | 0 | 0 | 1 | 0 | 0 |
| S60.7 | Superficial lesion trauma, multiple wrist, hand | Non severe | 0 | 0 | 1 | 0 | 0 | 0 | 0 | 0 |
| S60.8 | Superficial lesion trauma, wrist, hand, not coded elsewhere | Non severe | 0 | 0 | 1 | 0 | 0 | 0 | 0 | 0 |
| S60.9 | Superficial lesion trauma, wrist, hand, Without further information | Non severe | 0 | 0 | 1 | 0 | 0 | 0 | 0 | 0 |
| S61.0 | Open wound of finger, Without further information | Non severe | 0 | 0 | 0 | 0 | 0 | 1 | 0 | 0 |
| S61.1 | Open wound of finger with nail lesion | Non severe | 0 | 0 | 0 | 0 | 0 | 1 | 0 | 0 |
| S61.7 | Multiple open wounds, wrist, hand | Non severe | 0 | 0 | 0 | 0 | 0 | 1 | 0 | 0 |
| S61.8 | Open wound of other parts, wrist, hand | Non severe | 0 | 0 | 0 | 0 | 0 | 1 | 0 | 0 |
| S61.9 | Open wound of wrist, hand, Without further information | Non severe | 0 | 0 | 0 | 0 | 0 | 1 | 0 | 0 |
| S62.00 | Closed fracture of scaphoid bone of hand | Non severe | 0 | 0 | 0 | 0 | 0 | 1 | 0 | 0 |
| S62.01 | Open fracture of scaphoid bone of hand | Non severe | 0 | 0 | 0 | 0 | 0 | 1 | 0 | 0 |
| S62.10 | Closed fracture of another carpal bone | Non severe | 0 | 0 | 0 | 0 | 0 | 1 | 0 | 0 |
| S62.11 | Open fracture of another carpal bone | Non severe | 0 | 0 | 0 | 0 | 0 | 1 | 0 | 0 |
| S62.20 | Closed fracture of 1st metacarpal | Non severe | 0 | 0 | 0 | 0 | 0 | 1 | 0 | 0 |
| S62.21 | Open fracture of 1st metacarpal | Non severe | 0 | 0 | 0 | 0 | 0 | 1 | 0 | 0 |
| S62.30 | Closed fracture of another metacarpal bone | Non severe | 0 | 0 | 0 | 0 | 0 | 1 | 0 | 0 |
| S62.31 | Open fracture of another metacarpal bone | Non severe | 0 | 0 | 0 | 0 | 0 | 1 | 0 | 0 |
| S62.40 | Closed fractures, multiple metacarpals | Non severe | 0 | 0 | 0 | 0 | 0 | 1 | 0 | 0 |
| S62.41 | Open fractures, multiple metacarpals | Non severe | 0 | 0 | 0 | 0 | 0 | 1 | 0 | 0 |
| S62.50 | Closed fracture of thumb | Non severe | 0 | 0 | 0 | 0 | 0 | 1 | 0 | 0 |
| S62.51 | Open fracture of thumb | Non severe | 0 | 0 | 0 | 0 | 0 | 1 | 0 | 0 |
| S62.60 | Closed fracture of another finger | Non severe | 0 | 0 | 0 | 0 | 0 | 1 | 0 | 0 |
| S62.61 | Open fracture of another finger | Non severe | 0 | 0 | 0 | 0 | 0 | 1 | 0 | 0 |
| S62.70 | Closed fractures, multiple fingers | Non severe | 0 | 0 | 0 | 0 | 0 | 1 | 0 | 0 |
| S62.71 | Open fractures, multiple fingers | Non severe | 0 | 0 | 0 | 0 | 0 | 1 | 0 | 0 |
| S62.80 | Closed fracture of other parts, not coded elsewhere or Without further information wrist, hand | Non severe | 0 | 0 | 0 | 0 | 0 | 1 | 0 | 0 |
| S62.81 | Open fracture of other parts, not coded elsewhere or Without further information wrist, hand | Non severe | 0 | 0 | 0 | 0 | 0 | 1 | 0 | 0 |
| S63.0 | Dislocation of wrist | Non severe | 0 | 0 | 0 | 0 | 0 | 1 | 0 | 0 |
| S63.1 | Dislocation of finger | Non severe | 0 | 0 | 0 | 0 | 0 | 1 | 0 | 0 |
| S63.2 | Dislocation, multiple fingers | Non severe | 0 | 0 | 0 | 0 | 0 | 1 | 0 | 0 |
| S63.3 | Rupture trauma of wrist and carpal ligaments | Non severe | 0 | 0 | 0 | 0 | 0 | 1 | 0 | 0 |
| S63.4 | Rupture trauma of finger ligaments, metacarpal-phalangeal, interphalangeal | Non severe | 0 | 0 | 0 | 0 | 0 | 1 | 0 | 0 |
| S63.5 | Sprain of wrist | Non severe | 0 | 0 | 0 | 0 | 0 | 1 | 0 | 0 |
| S63.6 | Sprain of finger | Non severe | 0 | 0 | 0 | 0 | 0 | 1 | 0 | 0 |
| S63.7 | Sprain of other wrist or hand joints | Non severe | 0 | 0 | 0 | 0 | 0 | 1 | 0 | 0 |
| S64.0 | Lesion trauma of ulnar nerve, wrist, hand | Non severe | 0 | 0 | 0 | 0 | 0 | 1 | 0 | 0 |
| S64.1 | Lesion trauma of median nerve, wrist, hand | Non severe | 0 | 0 | 0 | 0 | 0 | 1 | 0 | 0 |
| S64.2 | Lesion trauma of radial nerve, wrist, hand | Non severe | 0 | 0 | 0 | 0 | 0 | 1 | 0 | 0 |
| S64.3 | Lesion trauma of collateral nerve of thumb | Non severe | 0 | 0 | 0 | 0 | 0 | 1 | 0 | 0 |
| S64.4 | Lesion trauma of collateral nerve of another finger | Non severe | 0 | 0 | 0 | 0 | 0 | 1 | 0 | 0 |
| S64.7 | Lesion trauma of multiple nerves, wrist, hand | Non severe | 0 | 0 | 0 | 0 | 0 | 1 | 0 | 0 |
| S64.8 | Lesion trauma of other nerves, wrist, hand | Non severe | 0 | 0 | 0 | 0 | 0 | 1 | 0 | 0 |
| S64.9 | Lesion trauma of a nerve, Without further information, wrist, hand | Non severe | 0 | 0 | 0 | 0 | 0 | 1 | 0 | 0 |
| S65.0 | Lesion trauma of cubital artery, wrist, hand | Non severe | 0 | 0 | 0 | 0 | 0 | 1 | 0 | 0 |
| S65.1 | Lesion trauma of radial artery, wrist, hand | Mild | 0 | 0 | 0 | 0 | 0 | 1 | 0 | 0 |
| S65.2 | Lesion trauma of superficial palmar arch | Non severe | 0 | 0 | 0 | 0 | 0 | 1 | 0 | 0 |
| S65.3 | Lesion trauma of deep palmar arch | Non severe | 0 | 0 | 0 | 0 | 0 | 1 | 0 | 0 |
| S65.4 | Lesion trauma of vessel, thumb | Non severe | 0 | 0 | 0 | 0 | 0 | 1 | 0 | 0 |
| S65.5 | Lesion trauma of vessel, another finger | Non severe | 0 | 0 | 0 | 0 | 0 | 1 | 0 | 0 |
| S65.7 | Lesion trauma of multiple blood vessels, wrist, hand | Non severe | 0 | 0 | 0 | 0 | 0 | 1 | 0 | 0 |
| S65.8 | Lesion trauma of other blood vessels, wrist, hand | Non severe | 0 | 0 | 0 | 0 | 0 | 1 | 0 | 0 |
| S65.9 | Lesion trauma of vessel, Without further information, wrist, hand | Non severe | 0 | 0 | 0 | 0 | 0 | 1 | 0 | 0 |
| S66.0 | Lesion trauma of long flexor tendon of thumb, wrist, hand | Non severe | 0 | 0 | 0 | 0 | 0 | 1 | 0 | 0 |
| S66.1 | Lesion trauma of flexor tendon of another finger, wrist, hand | Non severe | 0 | 0 | 0 | 0 | 0 | 1 | 0 | 0 |
| S66.2 | Lesion trauma of extensor tendon of thumb, wrist, hand | Non severe | 0 | 0 | 0 | 0 | 0 | 1 | 0 | 0 |
| S66.3 | Lesion trauma of extensor tendon of another finger, wrist, hand | Non severe | 0 | 0 | 0 | 0 | 0 | 1 | 0 | 0 |
| S66.4 | Lesion trauma of intrinsic tendons of thumb, wrist, hand | Non severe | 0 | 0 | 0 | 0 | 0 | 1 | 0 | 0 |
| S66.5 | Lesion trauma of intrinsic tendons of another finger, wrist, hand | Non severe | 0 | 0 | 0 | 0 | 0 | 1 | 0 | 0 |
| S66.6 | LES. TRAUMA. OF MULTIPLE FLEXOR TENDONS OF WRIST, HAND | Non severe | 0 | 0 | 0 | 0 | 0 | 1 | 0 | 0 |
| S66.7 | LES. TRAUMA. OF MULTIPLE EXTENSOR TENDONS OF WRIST, HAND | Non severe | 0 | 0 | 0 | 0 | 0 | 1 | 0 | 0 |
| S66.8 | LES. TRAUMA. OF OTHER TENDONS OF WRIST, HAND | Non severe | 0 | 0 | 0 | 0 | 0 | 1 | 0 | 0 |
| S66.9 | LES. TRAUMA. OF A TENDON, WRIST, HAND | Non severe | 0 | 0 | 0 | 0 | 0 | 1 | 0 | 0 |
| S67.0 | CRUSH INJURY OF THUMB AND OTHER FINGER | Non severe | 0 | 0 | 0 | 0 | 0 | 1 | 0 | 0 |
| S67.8 | CRUSH INJURY OF OTHER PARTS OF WRIST, HAND | Non severe | 0 | 0 | 0 | 0 | 0 | 1 | 0 | 0 |
| S68.0 | AMPUTATION INJURY OF THUMB | Non severe | 0 | 0 | 0 | 0 | 0 | 1 | 0 | 0 |
| S68.1 | AMPUTATION INJURY OF ANOTHER FINGER | Non severe | 0 | 0 | 0 | 0 | 0 | 1 | 0 | 0 |
| S68.2 | AMPUTATION INJURY OF TWO OR MORE FINGERS | Non severe | 0 | 0 | 0 | 0 | 0 | 1 | 0 | 0 |
| S68.3 | AMPUTATION INJURY ASSOCIATED WITH FINGER AND OTHER PARTS | Non severe | 0 | 0 | 0 | 0 | 0 | 1 | 0 | 0 |
| S68.4 | AMPUTATION INJURY OF THE HAND | Mild | 0 | 0 | 0 | 0 | 0 | 1 | 0 | 0 |
| S68.8 | AMPUTATION INJURY OF OTHER PARTS OF WRIST, HAND | Mild | 0 | 0 | 0 | 0 | 0 | 1 | 0 | 0 |
| S68.9 | AMPUTATION INJURY OF WRIST, HAND, Without further information LEVEL | Mild | 0 | 0 | 0 | 0 | 0 | 1 | 0 | 0 |
| S69.7 | LES. TRAUMA. OF MULTIPLE PARTS OF WRIST, HAND | Non severe | 0 | 0 | 0 | 0 | 0 | 1 | 0 | 0 |
| S69.8 | LES. TRAUMA. OF OTHER PARTS OF WRIST, HAND | Non severe | 0 | 0 | 0 | 0 | 0 | 1 | 0 | 0 |
| S70.0 | CONTUSION OF HIP | Non severe | 0 | 0 | 0 | 0 | 0 | 0 | 1 | 0 |
| S70.1 | CONTUSION OF THIGH | Non severe | 0 | 0 | 0 | 0 | 0 | 0 | 1 | 0 |
| S70.7 | LES. TRAUMA. OF MULTIPLE SURFACE PARTS OF HIP, THIGH | Non severe | 0 | 0 | 1 | 0 | 0 | 0 | 0 | 0 |
| S70.8 | LES. TRAUMA. OF SURFACE PARTS OF HIP, THIGH, OTHER | Non severe | 0 | 0 | 1 | 0 | 0 | 0 | 0 | 0 |
| S70.9 | LES. TRAUMA. OF SURFACE PARTS OF HIP, THIGH, Without further information | Non severe | 0 | 0 | 1 | 0 | 0 | 0 | 0 | 0 |
| S71.0 | OPEN WOUND OF HIP | Non severe | 0 | 0 | 0 | 0 | 0 | 0 | 1 | 0 |
| S71.1 | OPEN WOUND OF THIGH | Non severe | 0 | 0 | 0 | 0 | 0 | 0 | 1 | 0 |
| S71.7 | OPEN WOUNDS OF MULTIPLE PARTS OF HIP, THIGH | Mild | 0 | 0 | 0 | 0 | 0 | 0 | 1 | 0 |
| S71.8 | OPEN WOUNDS OF OTHER PARTS OF PELVIC AREA | Non severe | 0 | 0 | 0 | 0 | 0 | 0 | 1 | 0 |
| S72.00 | CLOSED FRACTURE OF FEMORAL NECK | Non severe | 0 | 0 | 0 | 0 | 0 | 0 | 1 | 0 |
| S72.01 | OPEN FRACTURE OF FEMORAL NECK | Mild | 0 | 0 | 0 | 0 | 0 | 0 | 1 | 0 |
| S72.10 | CLOSED FRACTURE OF TROCHANTER | Non severe | 0 | 0 | 0 | 0 | 0 | 0 | 1 | 0 |
| S72.11 | OPEN FRACTURE OF TROCHANTER | Mild | 0 | 0 | 0 | 0 | 0 | 0 | 1 | 0 |
| S72.20 | CLOSED FRACTURE OF SUBTROCHANTERIC PART OF FEMUR | Non severe | 0 | 0 | 0 | 0 | 0 | 0 | 1 | 0 |
| S72.21 | OPEN FRACTURE OF SUBTROCHANTERIC PART OF FEMUR | Mild | 0 | 0 | 0 | 0 | 0 | 0 | 1 | 0 |
| S72.30 | CLOSED FRACTURE OF FEMORAL SHAFT | Mild | 0 | 0 | 0 | 0 | 0 | 0 | 1 | 0 |
| S72.31 | OPEN FRACTURE OF FEMORAL SHAFT | Mild | 0 | 0 | 0 | 0 | 0 | 0 | 1 | 0 |
| S72.40 | CLOSED FRACTURE OF DISTAL FEMUR | Mild | 0 | 0 | 0 | 0 | 0 | 0 | 1 | 0 |
| S72.41 | OPEN FRACTURE OF DISTAL FEMUR | Mild | 0 | 0 | 0 | 0 | 0 | 0 | 1 | 0 |
| S72.70 | MULTIPLE CLOSED FRACTURES OF FEMUR | Mild | 0 | 0 | 0 | 0 | 0 | 0 | 1 | 0 |
| S72.71 | MULTIPLE OPEN FRACTURES OF FEMUR | Mild | 0 | 0 | 0 | 0 | 0 | 0 | 1 | 0 |
| S72.80 | CLOSED FRACTURE OF OTHER PARTS OF FEMUR | Non severe | 0 | 0 | 0 | 0 | 0 | 0 | 1 | 0 |
| S72.81 | OPEN FRACTURE OF OTHER PARTS OF FEMUR | Non severe | 0 | 0 | 0 | 0 | 0 | 0 | 1 | 0 |
| S72.90 | CLOSED FRACTURE OF FEMUR, Without further information PART | Non severe | 0 | 0 | 0 | 0 | 0 | 0 | 1 | 0 |
| S72.91 | OPEN FRACTURE OF FEMUR, Without further information PART | Mild | 0 | 0 | 0 | 0 | 0 | 0 | 1 | 0 |
| S73.0 | DISLOCATION OF HIP | Non severe | 0 | 0 | 0 | 0 | 0 | 0 | 1 | 0 |
| S73.1 | SPRAIN OF HIP | Non severe | 0 | 0 | 0 | 0 | 0 | 0 | 1 | 0 |
| S74.0 | LES. TRAUMA. OF SCIATIC NERVE IN HIP, THIGH | Non severe | 0 | 0 | 0 | 0 | 0 | 0 | 1 | 0 |
| S74.1 | LES. TRAUMA. OF FEMORAL NERVE IN HIP, THIGH | Non severe | 0 | 0 | 0 | 0 | 0 | 0 | 1 | 0 |
| S74.2 | LES. TRAUMA. OF CUTANEOUS NERVE IN HIP, THIGH | Non severe | 0 | 0 | 0 | 0 | 0 | 0 | 1 | 0 |
| S74.7 | LES. TRAUMA. OF MULTIPLE NERVES IN HIP, THIGH | Non severe | 0 | 0 | 0 | 0 | 0 | 0 | 1 | 0 |
| S74.8 | LES. TRAUMA. OF OTHER NERVES IN HIP, THIGH | Non severe | 0 | 0 | 0 | 0 | 0 | 0 | 1 | 0 |
| S74.9 | LES. TRAUMA. OF A NERVE, Without further information, IN HIP, THIGH | Non severe | 0 | 0 | 0 | 0 | 0 | 0 | 1 | 0 |
| S75.0 | LES. TRAUMA. OF FEMORAL ARTERY | Severe | 0 | 0 | 0 | 0 | 0 | 0 | 1 | 0 |
| S75.1 | LES. TRAUMA. OF FEMORAL VEIN IN HIP, THIGH | Mild | 0 | 0 | 0 | 0 | 0 | 0 | 1 | 0 |
| S75.2 | LES. TRAUMA. OF SAPHENOUS VEIN IN HIP, THIGH | Mild | 0 | 0 | 0 | 0 | 0 | 0 | 1 | 0 |
| S75.7 | LES. TRAUMA. OF MULTIPLE VASCULAR STRUCTURES IN HIP, THIGH | Mild | 0 | 0 | 0 | 0 | 0 | 0 | 1 | 0 |
| S75.8 | LES. TRAUMA. OF OTHER VASCULAR STRUCTURES IN HIP, THIGH | Mild | 0 | 0 | 0 | 0 | 0 | 0 | 1 | 0 |
| S75.9 | LES. TRAUMA. OF A BLOOD VESSEL, Without further information, IN HIP, THIGH | Mild | 0 | 0 | 0 | 0 | 0 | 0 | 1 | 0 |
| S76.0 | LES. TRAUMA. OF TENDONS OF HIP | Non severe | 0 | 0 | 0 | 0 | 0 | 0 | 1 | 0 |
| S76.1 | LES. TRAUMA. OF QUADRICEPS TENDON | Non severe | 0 | 0 | 0 | 0 | 0 | 0 | 1 | 0 |
| S76.2 | LES. TRAUMA. OF ADDUCTOR TENDON IN THIGH | Non severe | 0 | 0 | 0 | 0 | 0 | 0 | 1 | 0 |
| S76.3 | LES. TRAUMA. OF POSTERIOR TENDONS IN THIGH | Non severe | 0 | 0 | 0 | 0 | 0 | 0 | 1 | 0 |
| S76.4 | LES. TRAUMA. OF OTHER TENDONS IN THIGH | Non severe | 0 | 0 | 0 | 0 | 0 | 0 | 1 | 0 |
| S76.7 | LES. TRAUMA. OF MULTIPLE TENDONS IN HIP, THIGH | Non severe | 0 | 0 | 0 | 0 | 0 | 0 | 1 | 0 |
| S77.0 | CRUSH INJURY OF HIP | Severe | 0 | 0 | 0 | 0 | 0 | 0 | 1 | 0 |
| S77.1 | CRUSH INJURY OF THIGH | Mild | 0 | 0 | 0 | 0 | 0 | 0 | 1 | 0 |
| S77.2 | CRUSH INJURY OF HIP WITH THIGH | Severe | 0 | 0 | 0 | 0 | 0 | 0 | 1 | 0 |
| S78.0 | AMPUTATION INJURY OF HIP JOINT | Severe | 0 | 0 | 0 | 0 | 0 | 0 | 1 | 0 |
| S78.1 | AMPUTATION INJURY BETWEEN HIP AND KNEE | Severe | 0 | 0 | 0 | 0 | 0 | 0 | 1 | 0 |
| S78.9 | AMPUTATION INJURY OF HIP, THIGH, Without further information LEVEL | Severe | 0 | 0 | 0 | 0 | 0 | 0 | 1 | 0 |
| S79.7 | LES. TRAUMA. OF MULTIPLE PARTS OF HIP, THIGH | Mild | 0 | 0 | 0 | 0 | 0 | 0 | 1 | 0 |
| S79.8 | LES. TRAUMA. OF OTHER PARTS OF HIP, THIGH | Non severe | 0 | 0 | 0 | 0 | 0 | 0 | 1 | 0 |
| S80.0 | CONTUSION OF KNEE | Non severe | 0 | 0 | 0 | 0 | 0 | 0 | 1 | 0 |
| S80.1 | CONTUSION OF OTHER PARTS OF LEG | Non severe | 0 | 0 | 0 | 0 | 0 | 0 | 1 | 0 |
| S80.7 | LES. TRAUMA. OF MULTIPLE SURFACE PARTS OF LEG | Non severe | 0 | 0 | 1 | 0 | 0 | 0 | 0 | 0 |
| S80.8 | LES. TRAUMA. OF SURFACE PARTS OF LEG, OTHER | Non severe | 0 | 0 | 1 | 0 | 0 | 0 | 0 | 0 |
| S80.9 | LES. TRAUMA. OF SURFACE PARTS OF LEG, Without further information | Non severe | 0 | 0 | 1 | 0 | 0 | 0 | 0 | 0 |
| S81.0 | OPEN WOUND OF KNEE | Non severe | 0 | 0 | 0 | 0 | 0 | 0 | 1 | 0 |
| S81.7 | OPEN WOUNDS OF MULTIPLE PARTS OF LEG | Non severe | 0 | 0 | 0 | 0 | 0 | 0 | 1 | 0 |
| S81.8 | OPEN WOUND OF OTHER PARTS OF LEG | Non severe | 0 | 0 | 0 | 0 | 0 | 0 | 1 | 0 |
| S81.9 | OPEN WOUND OF LEG, Without further information PART | Non severe | 0 | 0 | 0 | 0 | 0 | 0 | 1 | 0 |
| S82.00 | CLOSED FRACTURE OF PATELLA | Non severe | 0 | 0 | 0 | 0 | 0 | 0 | 1 | 0 |
| S82.01 | OPEN FRACTURE OF PATELLA | Non severe | 0 | 0 | 0 | 0 | 0 | 0 | 1 | 0 |
| S82.10 | CLOSED FRACTURE OF UPPER TIBIA | Non severe | 0 | 0 | 0 | 0 | 0 | 0 | 1 | 0 |
| S82.11 | OPEN FRACTURE OF UPPER TIBIA | Non severe | 0 | 0 | 0 | 0 | 0 | 0 | 1 | 0 |
| S82.20 | CLOSED FRACTURE OF TIBIAL SHAFT | Non severe | 0 | 0 | 0 | 0 | 0 | 0 | 1 | 0 |
| S82.21 | OPEN FRACTURE OF TIBIAL SHAFT | Non severe | 0 | 0 | 0 | 0 | 0 | 0 | 1 | 0 |
| S82.30 | CLOSED FRACTURE OF LOWER TIBIA | Non severe | 0 | 0 | 0 | 0 | 0 | 0 | 1 | 0 |
| S82.31 | OPEN FRACTURE OF LOWER TIBIA | Non severe | 0 | 0 | 0 | 0 | 0 | 0 | 1 | 0 |
| S82.40 | CLOSED FRACTURE OF FIBULA ONLY | Non severe | 0 | 0 | 0 | 0 | 0 | 0 | 1 | 0 |
| S82.41 | OPEN FRACTURE OF FIBULA ONLY | Non severe | 0 | 0 | 0 | 0 | 0 | 0 | 1 | 0 |
| S82.50 | Closed fracture of the internal malleolus | Non severe | 0 | 0 | 0 | 0 | 0 | 0 | 1 | 0 |
| S82.51 | Open fracture of the internal malleolus | Non severe | 0 | 0 | 0 | 0 | 0 | 0 | 1 | 0 |
| S82.60 | Closed fracture of the external malleolus | Non severe | 0 | 0 | 0 | 0 | 0 | 0 | 1 | 0 |
| S82.61 | Open fracture of the external malleolus | Non severe | 0 | 0 | 0 | 0 | 0 | 0 | 1 | 0 |
| S82.70 | Multiple closed fractures of the leg | Non severe | 0 | 0 | 0 | 0 | 0 | 0 | 1 | 0 |
| S82.71 | Multiple open fractures of the leg | Non severe | 0 | 0 | 0 | 0 | 0 | 0 | 1 | 0 |
| S82.80 | Closed fractures of other parts of the leg | Non severe | 0 | 0 | 0 | 0 | 0 | 0 | 1 | 0 |
| S82.81 | Open fractures of other parts of the leg | Non severe | 0 | 0 | 0 | 0 | 0 | 0 | 1 | 0 |
| S82.90 | Closed fracture of the leg, specific part | Non severe | 0 | 0 | 0 | 0 | 0 | 0 | 1 | 0 |
| S82.91 | Open fracture of the leg, specific part | Non severe | 0 | 0 | 0 | 0 | 0 | 0 | 1 | 0 |
| S83.0 | Patellar dislocation | Non severe | 0 | 0 | 0 | 0 | 0 | 0 | 1 | 0 |
| S83.1 | Knee dislocation | Non severe | 0 | 0 | 0 | 0 | 0 | 0 | 1 | 0 |
| S83.2 | Recent meniscal tear | Non severe | 0 | 0 | 0 | 0 | 0 | 0 | 1 | 0 |
| S83.3 | Recent cartilage injury of the knee joint | Non severe | 0 | 0 | 0 | 0 | 0 | 0 | 1 | 0 |
| S83.4 | Lateral ligament sprain of the knee | Non severe | 0 | 0 | 0 | 0 | 0 | 0 | 1 | 0 |
| S83.5 | Cruciate ligament sprain of the knee | Non severe | 0 | 0 | 0 | 0 | 0 | 0 | 1 | 0 |
| S83.6 | Sprain of non-cruciate or other knee parts | Non severe | 0 | 0 | 0 | 0 | 0 | 0 | 1 | 0 |
| S83.7 | Trauma to multiple parts of the knee | Non severe | 0 | 0 | 0 | 0 | 0 | 0 | 1 | 0 |
| S84.0 | Trauma to the internal popliteal sciatic nerve | Non severe | 0 | 0 | 0 | 0 | 0 | 0 | 1 | 0 |
| S84.1 | Trauma to the external popliteal sciatic nerve | Non severe | 0 | 0 | 0 | 0 | 0 | 0 | 1 | 0 |
| S84.2 | Trauma to a cutaneous nerve of the leg | Non severe | 0 | 0 | 0 | 0 | 0 | 0 | 1 | 0 |
| S84.7 | Trauma to multiple nerves in the leg | Non severe | 0 | 0 | 0 | 0 | 0 | 0 | 1 | 0 |
| S84.8 | Trauma to other nerves in the leg | Non severe | 0 | 0 | 0 | 0 | 0 | 0 | 1 | 0 |
| S84.9 | Trauma to a sciatic nerve, specific part of the leg | Non severe | 0 | 0 | 0 | 0 | 0 | 0 | 1 | 0 |
| S85.0 | Trauma to the popliteal artery | Mild | 0 | 0 | 0 | 0 | 0 | 0 | 1 | 0 |
| S85.1 | Trauma to the tibial artery | Mild | 0 | 0 | 0 | 0 | 0 | 0 | 1 | 0 |
| S85.2 | Trauma to the peroneal artery | Mild | 0 | 0 | 0 | 0 | 0 | 0 | 1 | 0 |
| S85.3 | Trauma to the internal saphenous vein in the leg | Non severe | 0 | 0 | 0 | 0 | 0 | 0 | 1 | 0 |
| S85.4 | Trauma to the external saphenous vein in the leg | Non severe | 0 | 0 | 0 | 0 | 0 | 0 | 1 | 0 |
| S85.5 | Trauma to the popliteal vein | Mild | 0 | 0 | 0 | 0 | 0 | 0 | 1 | 0 |
| S85.7 | Trauma to multiple blood vessels in the leg | Mild | 0 | 0 | 0 | 0 | 0 | 0 | 1 | 0 |
| S85.8 | Trauma to other blood vessels in the leg | Non severe | 0 | 0 | 0 | 0 | 0 | 0 | 1 | 0 |
| S85.9 | Trauma to a blood vessel, specific part of the leg | Non severe | 0 | 0 | 0 | 0 | 0 | 0 | 1 | 0 |
| S86.0 | Trauma to the Achilles tendon | Non severe | 0 | 0 | 0 | 0 | 0 | 0 | 1 | 0 |
| S86.1 | Trauma to other posterior muscles and tendons in the leg | Non severe | 0 | 0 | 0 | 0 | 0 | 0 | 1 | 0 |
| S86.2 | Trauma to anterior muscles and tendons in the leg | Non severe | 0 | 0 | 0 | 0 | 0 | 0 | 1 | 0 |
| S86.3 | Trauma to peroneal muscles and tendons in the leg | Non severe | 0 | 0 | 0 | 0 | 0 | 0 | 1 | 0 |
| S86.7 | Trauma to multiple tendons in the leg | Non severe | 0 | 0 | 0 | 0 | 0 | 0 | 1 | 0 |
| S86.8 | Trauma to other tendons in the leg | Non severe | 0 | 0 | 0 | 0 | 0 | 0 | 1 | 0 |
| S86.9 | Trauma to a tendon, specific part of the leg | Non severe | 0 | 0 | 0 | 0 | 0 | 0 | 1 | 0 |
| S87.0 | Knee crush injury | Mild | 0 | 0 | 0 | 0 | 0 | 0 | 1 | 0 |
| S87.8 | Crush injury to other parts of the leg | Non severe | 0 | 0 | 0 | 0 | 0 | 0 | 1 | 0 |
| S88.0 | Traumatic amputation of the knee | Mild | 0 | 0 | 0 | 0 | 0 | 0 | 1 | 0 |
| S88.1 | Traumatic amputation between the knee and ankle | Severe | 0 | 0 | 0 | 0 | 0 | 0 | 1 | 0 |
| S88.9 | Traumatic amputation of the leg, specific part | Severe | 0 | 0 | 0 | 0 | 0 | 0 | 1 | 0 |
| S89.7 | Trauma to multiple parts of the leg | Non severe | 0 | 0 | 0 | 0 | 0 | 0 | 1 | 0 |
| S89.8 | Trauma to other parts of the leg | Non severe | 0 | 0 | 0 | 0 | 0 | 0 | 1 | 0 |
| S90.0 | Ankle contusion | Non severe | 0 | 0 | 0 | 0 | 0 | 0 | 1 | 0 |
| S90.1 | Contusion of a toe | Non severe | 0 | 0 | 0 | 0 | 0 | 0 | 1 | 0 |
| S90.2 | Contusion of a toe with nail injury | Non severe | 0 | 0 | 0 | 0 | 0 | 0 | 1 | 0 |
| S90.3 | Contusion of other parts of the foot | Non severe | 0 | 0 | 0 | 0 | 0 | 0 | 1 | 0 |
| S90.7 | Superficial trauma to multiple ankle or foot parts | Non severe | 0 | 0 | 1 | 0 | 0 | 0 | 0 | 0 |
| S90.8 | Superficial trauma to ankle, foot, non-cruciate parts | Non severe | 0 | 0 | 1 | 0 | 0 | 0 | 0 | 0 |
| S90.9 | Superficial trauma to ankle, foot, specific parts | Non severe | 0 | 0 | 1 | 0 | 0 | 0 | 0 | 0 |
| S91.0 | Open wound of the ankle | Non severe | 0 | 0 | 0 | 0 | 0 | 0 | 1 | 0 |
| S91.1 | Open wound of a toe, specific part | Non severe | 0 | 0 | 0 | 0 | 0 | 0 | 1 | 0 |
| S91.2 | Open wound of a toe with nail injury | Non severe | 0 | 0 | 0 | 0 | 0 | 0 | 1 | 0 |
| S91.3 | Open wound of other parts of the foot | Non severe | 0 | 0 | 0 | 0 | 0 | 0 | 1 | 0 |
| S91.7 | Multiple open wounds of ankle, foot | Non severe | 0 | 0 | 0 | 0 | 0 | 0 | 1 | 0 |
| S92.00 | Closed fracture of the calcaneus | Non severe | 0 | 0 | 0 | 0 | 0 | 0 | 1 | 0 |
| S92.01 | Open fracture of the calcaneus | Non severe | 0 | 0 | 0 | 0 | 0 | 0 | 1 | 0 |
| S92.10 | Closed fracture of the astragalus | Non severe | 0 | 0 | 0 | 0 | 0 | 0 | 1 | 0 |
| S92.11 | Open fracture of the astragalus | Non severe | 0 | 0 | 0 | 0 | 0 | 0 | 1 | 0 |
| S92.20 | Closed fracture of other tarsal bones | Non severe | 0 | 0 | 0 | 0 | 0 | 0 | 1 | 0 |
| S92.21 | Open fracture of other tarsal bones | Non severe | 0 | 0 | 0 | 0 | 0 | 0 | 1 | 0 |
| S92.30 | Closed fracture of metatarsal bones | Non severe | 0 | 0 | 0 | 0 | 0 | 0 | 1 | 0 |
| S92.31 | Open fracture of metatarsal bones | Non severe | 0 | 0 | 0 | 0 | 0 | 0 | 1 | 0 |
| S92.40 | Closed fracture of the big toe | Non severe | 0 | 0 | 0 | 0 | 0 | 0 | 1 | 0 |
| S92.41 | Open fracture of the big toe | Non severe | 0 | 0 | 0 | 0 | 0 | 0 | 1 | 0 |
| S92.50 | Closed fracture of another toe | Non severe | 0 | 0 | 0 | 0 | 0 | 0 | 1 | 0 |
| S92.51 | Open fracture of another toe | Non severe | 0 | 0 | 0 | 0 | 0 | 0 | 1 | 0 |
| S92.70 | Multiple closed fractures of the foot | Non severe | 0 | 0 | 0 | 0 | 0 | 0 | 1 | 0 |
| S92.71 | Multiple open fractures of the foot | Non severe | 0 | 0 | 0 | 0 | 0 | 0 | 1 | 0 |
| S92.90 | Closed fracture of the foot, specific part | Non severe | 0 | 0 | 0 | 0 | 0 | 0 | 1 | 0 |
| S92.91 | Open fracture of the foot, specific part | Non severe | 0 | 0 | 0 | 0 | 0 | 0 | 1 | 0 |
| S93.0 | Ankle dislocation | Non severe | 0 | 0 | 0 | 0 | 0 | 0 | 1 | 0 |
| S93.1 | Toe dislocation | Non severe | 0 | 0 | 0 | 0 | 0 | 0 | 1 | 0 |
| S93.2 | Rupture of ligaments in the ankle, foot | Non severe | 0 | 0 | 0 | 0 | 0 | 0 | 1 | 0 |
| S93.3 | Dislocation of non-cruciate or other parts of the foot | Non severe | 0 | 0 | 0 | 0 | 0 | 0 | 1 | 0 |
| S93.4 | Ankle sprain | Non severe | 0 | 0 | 0 | 0 | 0 | 0 | 1 | 0 |
| S93.5 | Toe sprain | Non severe | 0 | 0 | 0 | 0 | 0 | 0 | 1 | 0 |
| S93.6 | Sprain of non-cruciate or other parts of the foot | Non severe | 0 | 0 | 0 | 0 | 0 | 0 | 1 | 0 |
| S94.0 | Trauma to the external plantar nerve | Non severe | 0 | 0 | 0 | 0 | 0 | 0 | 1 | 0 |
| S94.1 | Trauma to the internal plantar nerve | Non severe | 0 | 0 | 0 | 0 | 0 | 0 | 1 | 0 |
| S94.2 | Trauma to the anterior tibial nerve, ankle, foot | Non severe | 0 | 0 | 0 | 0 | 0 | 0 | 1 | 0 |
| S94.3 | Trauma of the sensory cutaneous nerve level ankle, foot | Non severe | 0 | 0 | 0 | 0 | 0 | 0 | 1 | 0 |
| S94.7 | Trauma of multiple nerves level ankle, foot | Non severe | 0 | 0 | 0 | 0 | 0 | 0 | 1 | 0 |
| S94.8 | Trauma of other nerves level ankle, foot | Non severe | 0 | 0 | 0 | 0 | 0 | 0 | 1 | 0 |
| S94.9 | Trauma of a sciatic nerve level ankle, foot | Non severe | 0 | 0 | 0 | 0 | 0 | 0 | 1 | 0 |
| S95.0 | Trauma of the dorsal artery of the foot | Non severe | 0 | 0 | 0 | 0 | 0 | 0 | 1 | 0 |
| S95.1 | Trauma of the plantar artery of the foot | Non severe | 0 | 0 | 0 | 0 | 0 | 0 | 1 | 0 |
| S95.2 | Trauma of the dorsal vein of the foot | Non severe | 0 | 0 | 0 | 0 | 0 | 0 | 1 | 0 |
| S95.7 | Trauma of multiple vascular structures, blood vessels level ankle, foot | Non severe | 0 | 0 | 0 | 0 | 0 | 0 | 1 | 0 |
| S95.8 | Trauma of other vascular structures, blood vessels level ankle, foot | Non severe | 0 | 0 | 0 | 0 | 0 | 0 | 1 | 0 |
| S95.9 | Trauma of a blood vessel sciatic, level ankle, foot | Non severe | 0 | 0 | 0 | 0 | 0 | 0 | 1 | 0 |
| S96.0 | Trauma of a long flexor tendon of a toe, level ankle, foot | Non severe | 0 | 0 | 0 | 0 | 0 | 0 | 1 | 0 |
| S96.1 | Trauma of a long extensor tendon of a toe, level ankle, foot | Non severe | 0 | 0 | 0 | 0 | 0 | 0 | 1 | 0 |
| S96.2 | Trauma of an intrinsic tendon level ankle, foot | Non severe | 0 | 0 | 0 | 0 | 0 | 0 | 1 | 0 |
| S96.7 | Trauma of multiple tendons level ankle, foot | Non severe | 0 | 0 | 0 | 0 | 0 | 0 | 1 | 0 |
| S96.8 | Trauma of other tendons level ankle, foot | Non severe | 0 | 0 | 0 | 0 | 0 | 0 | 1 | 0 |
| S96.9 | Trauma of a tendon sciatic, level ankle, foot | Non severe | 0 | 0 | 0 | 0 | 0 | 0 | 1 | 0 |
| S97.0 | Crush injury of the ankle | Non severe | 0 | 0 | 0 | 0 | 0 | 0 | 1 | 0 |
| S97.1 | Crush injury of a toe | Non severe | 0 | 0 | 0 | 0 | 0 | 0 | 1 | 0 |
| S97.8 | Crush injury of other parts of the ankle, foot | Non severe | 0 | 0 | 0 | 0 | 0 | 0 | 1 | 0 |
| S98.0 | Amputation trauma of the foot, level ankle | Mild | 0 | 0 | 0 | 0 | 0 | 0 | 1 | 0 |
| S98.1 | Amputation trauma of a toe | Non severe | 0 | 0 | 0 | 0 | 0 | 0 | 1 | 0 |
| S98.2 | Amputation trauma of two or more toes | Non severe | 0 | 0 | 0 | 0 | 0 | 0 | 1 | 0 |
| S98.3 | Amputation trauma of other parts of the foot | Non severe | 0 | 0 | 0 | 0 | 0 | 0 | 1 | 0 |
| S98.4 | Amputation trauma of the foot, sciatic level | Mild | 0 | 0 | 0 | 0 | 0 | 0 | 1 | 0 |
| S99.7 | Trauma of multiple parts ankle, foot | Non severe | 0 | 0 | 0 | 0 | 0 | 0 | 1 | 0 |
| S99.8 | Trauma of unknown parts ankle, foot | Non severe | 0 | 0 | 0 | 0 | 0 | 0 | 1 | 0 |
| S99.9 | Trauma of ankle, foot sciatic level | Non severe | 0 | 0 | 0 | 0 | 0 | 0 | 1 | 0 |
| T04.2 | Crush injury of multiple parts of upper limb | Mild | 0 | 0 | 0 | 0 | 0 | 0 | 1 | 0 |
| T04.3 | Crush injury of multiple parts of lower limb | Mild | 0 | 0 | 0 | 0 | 0 | 0 | 1 | 0 |
| T04.4 | Crush injury of multiple parts of upper limb with lower limb | Mild | 0 | 0 | 0 | 0 | 0 | 0 | 1 | 0 |
| T05.0 | Amputation trauma of both hands | Severe | 0 | 0 | 0 | 0 | 0 | 0 | 1 | 0 |
| T05.1 | Amputation trauma of one hand and the other upper limb | Severe | 0 | 0 | 0 | 0 | 0 | 0 | 1 | 0 |
| T05.2 | Amputation trauma of both upper limbs | Severe | 0 | 0 | 0 | 0 | 0 | 0 | 1 | 0 |
| T05.3 | Amputation trauma of both feet | Severe | 0 | 0 | 0 | 0 | 0 | 0 | 1 | 0 |
| T05.4 | Amputation trauma of one foot and the other lower limb | Severe | 0 | 0 | 0 | 0 | 0 | 0 | 1 | 0 |
| T05.5 | Amputation trauma of both lower limbs | Severe | 0 | 0 | 0 | 0 | 0 | 0 | 1 | 0 |
| T05.6 | Amputation trauma of upper and lower limbs, any combination | Severe | 0 | 0 | 0 | 0 | 0 | 0 | 1 | 0 |
| T05.8 | Amputation trauma with other associations of body parts | Mild | 0 | 0 | 0 | 0 | 0 | 0 | 1 | 0 |
| T05.9 | Amputation trauma, multiple, sciatic level | Mild | 0 | 0 | 0 | 0 | 0 | 0 | 1 | 0 |
| T06.0 | Trauma of cervical nerve roots with trauma to nerves, spinal cord level neck | Non severe | 1 | 0 | 0 | 0 | 0 | 0 | 0 | 0 |
| T06.1 | Trauma of nerves and spinal cord of other body parts | Mild | 1 | 1 | 0 | 1 | 1 | 1 | 1 | 0 |
| T06.2 | Trauma of nerves of other parts of the body | Non severe | 1 | 1 | 0 | 1 | 1 | 1 | 1 | 0 |
| T06.3 | Trauma of vascular structures of other parts of the body | Mild | 1 | 1 | 0 | 1 | 1 | 1 | 1 | 0 |
| T06.5 | Trauma of thoracic organs with abdominal and pelvic organ trauma | Mild | 0 | 0 | 0 | 1 | 1 | 0 | 0 | 0 |
| T06.8 | Trauma of unknown parts of the body, unknown cause | Non severe | 1 | 1 | 1 | 1 | 1 | 1 | 1 | 0 |
| T09.4 | Trauma of nerve, nerve root, and plexus of the spine, sciatic level | Non severe | 1 | 0 | 0 | 1 | 1 | 0 | 0 | 0 |
| T09.6 | Amputation trauma of the trunk, sciatic level | Severe | 0 | 0 | 0 | 1 | 1 | 0 | 0 | 0 |
| T09.8 | Trauma of unknown parts of the trunk, sciatic level | Non severe | 1 | 0 | 0 | 1 | 1 | 0 | 0 | 0 |
| T11.3 | Trauma of a sciatic nerve of the upper limb, sciatic level | Non severe | 0 | 0 | 0 | 1 | 1 | 0 | 0 | 0 |
| T11.4 | Trauma of a blood vessel sciatic of the upper limb, sciatic level | Mild | 0 | 0 | 0 | 0 | 0 | 1 | 0 | 0 |
| T11.6 | Amputation trauma of the upper limb, sciatic level | Mild | 0 | 0 | 0 | 0 | 0 | 1 | 0 | 0 |
| T11.8 | Trauma of unknown parts of the upper limb, sciatic level | Non severe | 0 | 0 | 0 | 0 | 0 | 1 | 0 | 0 |
| T13.3 | Trauma of a sciatic nerve of the lower limb, sciatic level | Non severe | 0 | 0 | 0 | 0 | 0 | 0 | 1 | 0 |
| T13.4 | Trauma of a blood vessel sciatic of the lower limb, sciatic level | Mild | 0 | 0 | 0 | 0 | 0 | 0 | 1 | 0 |
| T13.6 | Amputation trauma of the lower limb, sciatic level | Mild | 0 | 0 | 0 | 0 | 0 | 0 | 1 | 0 |
| T13.8 | Trauma of unknown parts of the lower limb, sciatic level | Non severe | 0 | 0 | 0 | 0 | 0 | 0 | 1 | 0 |
| T79.0 | Gas embolism | Mild | 0 | 0 | 0 | 0 | 0 | 0 | 0 | 1 |
| T79.1 | Fat embolism | Mild | 0 | 0 | 0 | 0 | 0 | 0 | 0 | 1 |
| T79.2 | Hemorrhage, secondary and recurrent trauma | Mild | 0 | 0 | 0 | 0 | 0 | 0 | 0 | 1 |
| T79.3 | Post-traumatic infection of a wound, unknown cause | Non severe | 0 | 0 | 0 | 0 | 0 | 0 | 0 | 1 |
| T79.4 | Traumatic shock | Severe | 0 | 0 | 0 | 0 | 0 | 0 | 0 | 1 |
| T79.5 | Traumatic anuria | Mild | 0 | 0 | 0 | 0 | 0 | 0 | 0 | 1 |
| T79.6 | Traumatic ischemia of a muscle | Non severe | 0 | 0 | 0 | 0 | 0 | 0 | 0 | 1 |
| T79.7 | Subcutaneous emphysema of traumatic origin | Mild | 0 | 0 | 0 | 0 | 0 | 0 | 0 | 1 |
| T79.8 | Early complications of trauma, unknown cause | Non severe | 0 | 0 | 0 | 0 | 0 | 0 | 0 | 1 |

## **Supplementary material 2**: Comparison between matched and unmatched patients

| **Variable** | | **Unmatched Cohort** | **Matched Cohort** | **P-value** |
| --- | --- | --- | --- | --- |
| **Comorbidities and mechanism of injury** | | | | |
| *Age (years)* | Min / Max | 13 / 93 | 13 / 96 | < 0. 001 |
|  | Med [IQR] | 32 [24 ; 47] | 35 [25 ; 51] |  |
|  | N (Missing Value) | 2364 (3) | 2629 (0) |  |
| *Gender* | Female | 466 (19.8%) | 550 (20.9%) | 0.31 |
|  | Male | 1892 (80.2%) | 2079 (79.1%) |  |
|  | Missing Value | 9 | 0 |  |
| *Pregnancy* | Male, Not applicable | 1892 (81.9%) | 2079 (82.0%) | 0.70 |
|  | No | 404 (17.5%) | 445 (17.6%) |  |
|  | Yes | 13 (0.6%) | 10 (0.4%) |  |
|  | Missing Value | 58 | 95 |  |
| *Mechanism of injury* | Blunt force trauma (non-penetrating) | 71 (3%) | 53 (2.0%) | < 0.001 |
|  | Fall from height | 516 (21.8%) | 696 (26.5%) |  |
|  | Gunshot | 125 (5.3%) | 126 (4.8%) |  |
|  | Other | 81 (3.4%) | 63 (2.4%) |  |
|  | Other blunt force trauma | 1 (0.0%) | 5 (0.2%) |  |
|  | Other penetrating trauma | 8 (0.3%) | 2 (0.1%) |  |
|  | Public road accident (bike) | 83 (3.5%) | 96 (3.7%) |  |
|  | Public road accident (car, truck, bus) | 315 (13.3%) | 322 (12.3%) |  |
|  | Public road accident (motorized two-wheelers) | 484 (20.5%) | 517 (19.7%) |  |
|  | Public road accident (pedestrian) | 171 (7.2%) | 303 (11.5%) |  |
|  | Public road accident motorized personal mobility device | 15 (0.6%) | 12 (0.5%) |  |
|  | Stabbing weapon | 446 (18.8%) | 360 (13.7%) |  |
|  | Traffic accident (plane, boat, train, other) | 28 (1.2%) | 60 (2.3%) |  |
|  | Unknown | 23 (1.0%) | 14 (0.5%) |  |
| **Initial trauma severity of injury** | | | | |
| *Time elasped between departure of emergency rapid response unit from the hospital and arrival on accident scene (min)* | Min / Max | 0 / 74 | 0 / 49 | 0.016 |
|  | Med [IQR] | 8 [5 ; 12] | 9 [5 ; 13] |  |
|  | N (Missing Value) | 1623 (744) | 1922 (707) |  |
| *First overall GCS assessment* | Min / Max | 3 / 15 | 3 / 15 | < 0.001 |
|  | Med [IQR] | 15 [14 ; 15] | 15 [9 ; 15] |  |
|  | N (Missing Value) | 2338 (29) | 2612 (17) |  |
| *Cardiac arrest before hospital admission* | No | 2297 (97.6%) | 2456 (93.5%) | < 0.001 |
|  | Yes | 57 (2.4%) | 172 (6.5%) |  |
|  | Missing Value | 13 | 1 |  |
| *Diastolic blood pressure at emergency rapid response unit arrival (mmHg)* | Min / Max | 0 / 145 | 0 / 155 | < 0.001 |
|  | Med [IQR] | 80 [70 ; 90] | 78 [62.75 ; 88] |  |
|  | N (Missing Value) | 1701 (666) | 1896 (733) |  |
| *Systolic blood pressure at emergency rapid response unit arrival (mmHg)* | Min / Max | 0 / 222 | 0 / 250 | < 0. 001 |
|  | Med [IQR] | 130 [116 ; 143] | 124 [104 ; 140] |  |
|  | N (Missing Value) | 1726 (641) | 1957 (672) |  |
| **Clinical trauma severity at hospital admission** | | | | |
| *Time elapsed between emergency rapid response unit arrival on accident scene and hospital admission (min)* | Min / Max | 0 / 680 | 1 / 1380 | < 0.001 |
|  | Med [IQR] | 60 [45 ; 9] | 66 [49 ; 90.5] |  |
|  | N (Missing Value) | 1899 (468) | 2219 (410) |  |
| *Working hours* | Daytime | 609 (25.8%) | 757 (28.8%) | 0.020 |
|  | Nightime | 1748 (74.16%) | 1872 (71.2%) |  |
|  | Missing Value | 10 | 0 |  |
| *Sedation at hospital admission* | No | 1992 (84.5%) | 1773 (67.5%) | < 0.001 |
|  | Yes | 366 (15.5%) | 855 (32.5%) |  |
|  | Missing Value | 9 | 1 |  |
| *GCS assessment (for unsedated patients)* | Min / Max | 3 / 15 | 3 / 15 | < 0.001 |
|  | Med [IQR] | 15 [15 ; 15] | 15 [14 ; 15] |  |
|  | N (Missing Value) | 1992 (375) | 1773 (856) |  |
| *Systolic blood pressure (mmHg)* | Min / Max | 0 / 250 | 0 / 241 | < 0.001 |
|  | Med [IQR] | 130 [116 ; 143] | 123 [105 ; 140] |  |
|  | N (Missing Value) | 2327 (40) | 2597 (32) |  |
| *Diastolic blood pressure (mmHg)* | Min / Max | 0 / 150 | 0 / 167 | < 0.001 |
|  | Med [IQR] | 77 [66 ; 88] | 73 [60 ; 85] |  |
|  | N (Missing Value) | 2327 (40) | 2596 (33) |  |
| *Lactate (mmol/l)* | Min / Max | 0 / 27 | 0 / 25 | < 0.001 |
|  | Med [IQR] | 1 [1 ; 2] | 2 [1 ; 3] |  |
|  | N (Missing Value) | 1935 (432) | 2385 (244) |  |
| *Mechanical ventilation at hospital admission* | No | 1809 (77.6%) | 1511 (57.7%) | < 0.001 |
|  | Yes | 523 (22.4%) | 1106 (42.3%) |  |
|  | Missing Value | 35 | 12 |  |
| **Initial management of trauma at hospital admission** | | | | |
| *Surgery or interventional radiology procedure before CT scan* | No | 1973 (95.1%) | 2246 (93.3%) | 0.008 |
|  | Yes | 101 (4.9%) | 162 (6.7%) |  |
|  | Missing Value | 293 | 221 |  |
| *Number of red blood cell units received before leaving the emergency trauma room* | Min / Max | 0 / 10 | 0 / 10 | < 0.001 |
|  | Med [IQR] | 0 [0 ; 0] | 0 [0 ; 1] |  |
|  | N (Missing Value) | 1371 (996) | 1809 (820) |  |
| *Number of platelets concentrates before leaving the emergency trauma room* | Min / Max | 0 / 1 | 0 / 4 | < 0.001 |
|  | Med [IQR] | 0 [0 ; 0] | 0 [0 ; 0] |  |
|  | N (Missing Value) | 1369 (998) | 1809 (820) |  |
| *Number of fresh frozen plasma units before leaving the emergency trauma room* | Min / Max | 0 / 6 | 0 / 10 | < 0.001 |
|  | Med [IQR] | 0 [0 ; 0] | 0 [0 ; 0] |  |
|  | N (Missing Value) | 1372 (995) | 1809 (820) |  |
| **Final diagnosis for trauma injuries and severity scores** | | | | |
| *Hemorrhagic shock (4 RBC units in 6 hours or less)* | No | 2040 (95.5%) | 1998 (83.7%) | < 0.001 |
|  | Yes | 97 (4.5%) | 389 (16.3%) |  |
|  | Missing Value | 230 | 242 |  |
| *Hemorrhagic shock (1 RBC unit in 6 hours or less)* | No | 208 (92.0%) | 179 (74.0%) | < 0.001 |
|  | Yes | 18 (8.0%) | 63 (26.0%) |  |
|  | Missing Value | 2141 | 2387 |  |
| *Head trauma (according to CT scan)* | No | 1945 (82.5%) | 1590 (60.7%) | < 0.001 |
|  | Yes | 414 (17.6%) | 1031 (39.3%) |  |
|  | Missing Value | 8 | 8 |  |
| *SAPS II* | Min / Max | 0 / 111 | 0 / 107 | < 0.001 |
|  | Med [IQR] | 17 [11 ; 28] | 30 [18 ; 48] |  |
|  | N (Missing Value) | 2347 (20) | 2621 (8) |  |
| *SOFA score* | Min / Max | 0 / 20 | 0 / 24 | < 0.001 |
|  | Med [IQR] | 1 [0 ; 3] | 4 [1 ; 9] |  |
|  | N (Missing Value) | 2318 (49) | 2596 (33) |  |
| *Surgery or interventional radiology procedure during hospital stay* | No | 114 (8.8%) | 95 (5.9%) | 0.003 |
|  | Yes | 1182 (91.2%) | 1503 (94.1%) |  |
|  | Missing Value | 1071 | 1031 |  |
| **AIS and Trauma Injury Severity Score** | | | | |
| *AIS Head and neck* | Min / Max | 0 / 6 | 0 / 6 | < 0.001 |
|  | Med [IQR] | 0 [0 ; 2] | 2 [0 ; 3] |  |
|  | N (Missing Value) | 2354 (13) | 2625 (4) |  |
| *AIS Face* | Min / Max | 0 / 4 | 0 / 4 | < 0.001 |
|  | Med [IQR] | 0 [0 ; 1] | 0 [0 ; 1] |  |
|  | N (Missing Value) | 2354 (13) | 2625 (4) |  |
| *AIS Chest* | Min / Max | 0 / 6 | 0 / 6 | < 0.001 |
|  | Med [IQR] | 0 [0 ; 2] | 2 [0 ; 3] |  |
|  | N (Missing Value) | 2354 (13) | 2625 (4) |  |
| *AIS Abdomen, pelvic contents* | Min / Max | 0 / 6 | 0 / 6 | < 0.001 |
|  | Med [IQR] | 0 [0 ; 0] | 0 [0 ; 2] |  |
|  | N (Missing Value) | 2354 (13) | 2625 (4) |  |
| *AIS Extremity, pelvic girdle* | Min / Max | 0 / 5 | 0 / 5 | < 0.001 |
|  | Med [IQR] | 1 [0 ; 2] | 2 [0 ; 3] |  |
|  | N (Missing Value) | 2354 (13) | 2625 (4) |  |
| *AIS External* | Min / Max | 0 / 5 | 0 / 4 | < 0.001 |
|  | Med [IQR] | 0 [0 ; 0] | 0 [0 ; 0] |  |
|  | N (Missing Value) | 2354 (13) | 2625 (4) |  |
| *Injury Severity Score* | Min / Max | 0 / 75 | 0 / 75 | < 0.001 |
|  | Med [IQR] | 9 [4 ; 13] | 19 [11 ; 29] |  |
|  | N (Missing Value) | 2355 (12) | 2625 (4) |  |
| *Trauma Injury Severity Score* | Min / Max | 0 / 1 | 0 / 1 | < 0.001 |
|  | Med [IQR] | 0.01 [0.01 ; 0.02] | 0.03 [0.01 ; 0.18] |  |
|  | N (Missing Value) | 2223 (144) | 2523 (106) |  |
| *Trauma Injury Severity Score by category* | [0, 9)  [9,16)  [16,25)  [25,75]  NA | 1128 (47.9%)  685 (29.09%)  256 (10.87%)  286 (12.14%)  12 | 361 (13.75%)  592 (22.55%)  706 (26.9%)  966 (36.8%)  4 | < 0.001 |
| **Outcomes during hospitalization** | | | | |
| *Death during hospital stay* | No | 2214 (94.5%) | 2246 (85.4%) | < 0.001 |
|  | Yes | 130 (5.6%) | 383 (14.6%) |  |
|  | Missing Value | 23 | 0 |  |
| *Number of ventilator free days* | Min / Max | 0 / 28 | 0 / 28 | < 0.001 |
|  | Med [IQR] | 28 [27 ; 28] | 26 [6 ; 28] |  |
|  | N (Missing Value) | 1699 (668) | 2292 (337) |  |
| *Acute respiratory distress syndrome* | No | 1886 (96.1%) | 2134 (89.4%) | < 0.001 |
|  | Yes | 76 (3.9%) | 252 (10.6%) |  |
|  | Missing Value | 405 | 243 |  |
| *Renal replacement therapy* | No | 707 (98.6%) | 1093 (96.8%) | 0.02 |
|  | Yes | 10 (1.4%) | 36 (3.2%) |  |
|  | Missing Value | 1650 | 1500 |  |
| *Length of hospital stay (days)* | Min / Max | 1 / 454 | 0 / 342 | < 0.001 |
|  | Med [IQR] | 4 [2 ; 12] | 11 [4 ; 30] |  |
|  | N (Missing Value) | 2266 (101) | 2629 (0) |  |

Wilcoxon rank sum test or Pearson's Chi-squared test were used as appropriate. AIS, Abreviated Injury Scale; CT, Comptuted tomography; FFP, Fresh Frozen Plasma; GCS, Glasgow Coma Score; IQR, Inter Quartile Range; MV, Missing Values; RBC, Red Blood Cell; SAPS II, Simplified Acute Physiology Score 2; Std, Standard deviation; SOFA, Sequential Organ Failure Assessment.

## Supplementary **material 3**: Compiled ICD-10 codes

| **Diagnosis** | **ICD-10** |
| --- | --- |
| Shock diagnostic codes | R57, R570, R571, R572, R578, R579, T794 |
| Administration of catecholamines | EQLF003, EQLF001 |
| Significant vascular filling | EQLF002 |
| Coma | R40, R400, R401, R402, R4028, R4018 |
| Penetrating injuries | S0601, S0611, S0621, S0631, S0641, S0651, S0661, S0681, S0691, S2681, S2691, S2701, S2711, S2721, S2731, S2741, S2761, S3601, S3621, S3631, S3641, S3651, S3661, S3681, S3701, S3711, S3721, S3731, S3741, S3751, S3761, S3771, S37810, S37818, S054, S055, S056 |
| Open fracture | **S02.01**, **S02.11**, S02.21, S02.31, S02.41, S02.51, S02.61, **S02.71**, S02.81, S12.01, S12.11, S12.21, **S12.71**, S12.81, S12.91, S22.01, **S22.11**, S22.21, S22.31, S22.41, **S22.51**, S22.81, S32.01, S32.11, S32.21, S32.31, S32.41, S32.51, **S32.71**, S32.81, S42.01, S42.11, S42.21, S42.31, S42.41, S42.71, S42.81, S42.91, S52.01, S52.11, S52.21, S52.31, S52.41, S52.51, S52.61, S52.71, S52.81, S52.91, S62.01, S62.11, S62.21, S62.31, S62.41, S62.51, S62.61, S62.71, S62.81, S72.01, S72.11, S72.21, S72.31, S72.41, S72.71, S72.81, S72.91, S82.01, S82.11, S82.21, S82.31, S82.41, S82.51, S82.61, S82.71, S82.81, S82.91, S92.01, S92.11, S92.21, S92.31, S92.41, S92.51, S92.71, S92.91 |
| Closed fracture | S02.00, S02.10, S02.20, S02.30, S02.40, S02.50, S02.60, S02.70, S02.80, S12.00, S12.10, S12.20, S12.70, S12.80, S12.90, S22.00, S22.10, S22.20, S22.30, S22.40, **S22.50**, S22.80, S32.00, S32.10, S32.20, S32.30, S32.40, S32.50, **S32.70**, S32.80, S42.00, S42.10, S42.20, S42.30, S42.40, S42.70, S42.80, S42.90, S52.00, S52.10, S52.20, S52.30, S52.40, S52.50, S52.60, S52.70, S52.80, S52.90, S62.00, S62.10, S62.20, S62.30, S62.40, S62.50, S62.60, S62.70, S62.80, S72.00, S72.10, S72.20, S72.30, S72.40, S72.70, S72.80, S72.90, S82.00, S82.10, S82.20, S82.30, S82.40, S82.50, S82.60, S82.70, S82.80, S82.90, S92.00, S92.10, S92.20, S92.30, S92.40, S92.50, S92.70, S92.90 |
| Organ traumatic lesion with internal wound | S05.4, S05.5, S05.6, **S06.01**, **S06.11**, **S06.21**, **S06.31**, **S06.41**, **S06.51**, **S06.61**, S06.81, S06.91, **S26.81**, **S26.91**, S27.01, **S27.11**, **S27.21**, S27.31, **S27.41**, S27.61, **S36.01**, **S36.21**, **S36.31**, **S36.41**, **S36.51**, **S36.61**, S36.81, **S37.01**, S37.11, S37.21, S37.31, S37.41, S37.51, S37.61, **S37.71**, **S37.810**, S37.818 |
| Organ traumatic lesion without internal wound | S06.00, **S06.10**, **S06.20**, **S06.30**, **S06.40**, **S06.50**, S06.60, S06.80, S06.90, **S26.80**, **S26.90**, S27.00, S27.10, S27.20, S27.30, S27.40, S27.60, S36.00, S36.20, S36.30, S36.40, S36.50, S36.60, S36.80, S37.00, S37.10, S37.20, S37.30, S37.40, S37.50, S37.60, **S37.70**, S37.800, S37.808 |
| Organ traumatic lesion without details on internal wound | S05.0, S05.1, S05.2, S05.3, S05.4, S05.5, S05.6, **S05.7**, S05.8, S05.9, S06.0, S06.00, **S06.01**, **S06.1**, **S06.10**, **S06.11**, **S06.2**, **S06.20**, **S06.21**, **S06.3**, **S06.30**, **S06.31**, **S06.4**, **S06.40**, **S06.41**, **S06.5**, **S06.50**, **S06.51**, S06.6, S06.60, **S06.61**, **S06.7**, **S06.70**, **S06.71**, S06.8, S06.80, S06.81, S06.9, S06.90, S06.91, **S26.0**, **S26.00**, **S26.01**, **S26.8**, **S26.80**, **S26.81**, **S26.9**, **S26.90**, **S26.91**, **S27.0**, S27.00, S27.01, S27.1, S27.10, **S27.11**, S27.2, S27.20, **S27.21**, S27.3, S27.30, S27.31, S27.4, S27.40, **S27.41**, **S27.5**, **S27.50**, **S27.51**, S27.6, S27.60, S27.61, S27.7, S27.70, S27.71, S27.8, S27.80, S27.81, S36.0, S36.00, **S36.01**, S36.1, S36.10, **S36.11**, S36.2, S36.20, **S36.21**, S36.3, S36.30, **S36.31**, S36.4, S36.40, **S36.41**, S36.5, S36.50, **S36.51**, S36.6, S36.60, **S36.61**, **S36.7**, **S36.70**, **S36.71**, S36.8, S36.80, S36.81, S37.0, S37.00, **S37.01**, S37.1, S37.10, S37.11, S37.2, S37.20, S37.21, S37.3, S37.30, S37.31, S37.4, S37.40, S37.41, S37.5, S37.50, S37.51, S37.6, S37.60, S37.61, **S37.7**, **S37.70**, **S37.71**, S37.800, S37.808, **S37.810**, S37.818 |
| Traumatic lesion of limbs, nerves, vessels, muscles or tendons | S04.0, S04.1, S04.2, S04.3, S04.4, S04.5, S04.6, S04.7, S04.8, S04.9, S09.0, S09.1, S09.7, S09.8, **S14.1**, S14.2, S14.3, S14.4, S14.5, S14.6, **S15.0**, **S15.1**, S15.2, **S15.3**, S15.7, S15.8, S15.9, S16, S19.7, S19.8, S24.1, S24.2, S24.3, S24.4, S24.5, S24.6, **S25.0**, **S25.1**, **S25.2**, S25.3, **S25.4**, S25.5, **S25.7**, S25.80, S25.88, S25.9, S29.0, S29.7, S29.8, S34.1, S34.2, S34.3, S34.4, S34.5, S34.6, S34.8, **S35.0**, **S35.1**, **S35.2**, **S35.3**, **S35.4**, **S35.5**, **S35.7**, S35.8, S35.9, S39.0, **S39.6**, **S39.7**, S39.8, S44.0, S44.1, S44.2, S44.3, S44.4, S44.5, S44.7, S44.8, S44.9, **S45.0**, **S45.1**, S45.2, S45.3, S45.7, S45.8, S45.9, S46.0, S46.1, S46.2, S46.3, S46.7, S46.8, S46.9, S49.7, S49.8, S54.0, S54.1, S54.2, S54.3, S54.7, S54.8, S54.9, S55.0, S55.1, S55.2, S55.7, S55.8, S55.9, S56.0, S56.1, S56.2, S56.3, S56.4, S56.5, S56.7, S56.8, S59.7, S59.8, S64.0, S64.1, S64.2, S64.3, S64.4, S64.7, S64.8, S64.9, S65.0, S65.1, S65.2, S65.3, S65.4, S65.5, S65.7, S65.8, S65.9, S66.0, S66.1, S66.2, S66.3, S66.4, S66.5, S66.6, S66.7, S66.8, S66.9, S69.7, S69.8, S74.0, S74.1, S74.2, S74.7, S74.8, S74.9, **S75.0**, S75.1, S75.2, S75.7, S75.8, S75.9, S76.0, S76.1, S76.2, S76.3, S76.4, S76.7, S79.7, S79.8, S83.7, S84.0, S84.1, S84.2, S84.7, S84.8, S84.9, S85.0, S85.1, S85.2, S85.3, S85.4, S85.5, S85.7, S85.8, S85.9, S86.0, S86.1, S86.2, S86.3, S86.7, S86.8, S86.9, S89.7, S89.8, S94.0, S94.1, S94.2, S94.3, S94.7, S94.8, S94.9, S95.0, S95.1, S95.2, S95.7, S95.8, S95.9, S96.0, S96.1, S96.2, S96.7, S96.8, S96.9, S99.7, S99.8, S99.9, T06.0, T06.1, T06.2, T06.3, T06.5, T06.8, T09.4, T09.8, T11.3, T11.4, T11.8, T13.3, T13.4, T13.8 |
| Open wound | S01.0, S01.1, S01.2, S01.3, S01.4, S01.5, S01.7, S01.8, **S11.0**, S11.1, **S11.2**, S11.7, S11.8, S11.9, S21.0, S21.1, S21.2, S21.7, S21.8, S21.9, S31.0, S31.1, S31.2, S31.3, S31.4, S31.5, S31.7, S31.8, S41.0, S41.1, S41.7, S41.8, S51.0, S51.7, S51.8, S51.9, S61.0, S61.1, S61.7, S61.8, S61.9, S71.0, S71.1, S71.7, S71.8, S81.0, S81.7, S81.8, S81.9, S91.0, S91.1, S91.2, S91.3, S91.7 |
| Internal bleeding | **S06.4**, **S06.40**, **S06.41**, **S06.5**, **S06.50**, **S06.51**, S06.6, S06.60, **S06.61**, **S26.0**, **S26.00**, **S26.01**, S27.1, S27.10, **S27.11**, S27.2, S27.20, **S27.21**, T79.2 |
| Amputation | S08.1, S08.8, S08.9, **S18**, **S28.1**, S38.2, S38.3, **S48.0**, **S48.1**, **S48.9**, **S58.0**, **S58.1**, **S58.9**, S68.0, S68.1, S68.2, S68.3, S68.4, S68.8, S68.9, **S78.0**, **S78.1**, **S78.9**, S88.0, **S88.1**, **S88.9**, S98.0, S98.1, S98.2, S98.3, S98.4, **T05.0**, **T05.1**, **T05.2**, **T05.3**, **T05.4**, **T05.5**, **T05.6**, T05.8, T05.9, **T09.6**, T11.6, T13.6 |
| Laceration | S05.2, S05.3 |
| Organ crushing | **S07.0**, **S07.1**, **S07.8**, S07.9, **S17.0**, S17.8, S17.9, **S28.0**, S38.0, S38.10, S38.18, S47.+0, S47.+8, S57.0, S57.8, S57.9, S67.0, S67.8, **S77.0**, S77.1, **S77.2**, S87.0, S87.8, S97.0, S97.1, S97.8, T04.2, T04.3, T04.4 |
| Superficial wound | S00.0, S00.2, S00.3, S00.4, S00.5, S00.7, S00.8, S10.1, S10.7, S10.8, S10.9, S20.1, S20.3, S20.4, S20.7, S20.8, S30.7, S30.8, S40.7, S40.8, S40.9, S50.7, S50.8, S50.9, S60.7, S60.8, S60.9, S70.7, S70.8, S70.9, S80.7, S80.8, S80.9, S90.7, S90.8, S90.9 |
| Luxation | S03.0, S03.1, S03.2, S03.3, S13.1, S13.2, S13.3, S23.1, S23.2, S33.1, S33.2, S33.3, S43.0, S43.1, S43.2, S43.3, S53.0, S53.1, S63.0, S63.1, S63.2, S73.0, S83.0, S83.1, S93.0, S93.1, S93.3 |
| Contusion | S00.1, S05.1, S10.0, S20.0, S20.2, S30.0, S30.1, S30.2, S40.0, S50.0, S50.1, S60.0, S60.1, S60.2, S70.0, S70.1, S80.0, S80.1, S90.0, S90.1, S90.2, S90.3 |

## **Supplementary Material 4**: Overall Traumatic lesions according to ICD-10 coding

| **Variables** |  | **Value** |
| --- | --- | --- |
| **Location of hospitalization, according the hospital coding unit** | | |
| *At least one ICU admission* | Yes | 2629 (100.0%) |
| *At least one surgical ward admission* | No | 1117 (42.5%) |
|  | Yes | 1512 (57.5%) |
| *At least one medical ward admission* | No | 2284 (86.9%) |
|  | Yes | 345 (13.1%) |
| **Overall traumatic lesions according to ICD-10 coding** | | |
| *Traumatic lesion* | 1 | 99 (3.8%) |
|  | 2 | 159 (6.1%) |
|  | 3 | 257 (9.8%) |
|  | 4 | 260 (9.9%) |
|  | 5 or more | 1854 (70.5%) |
| *Open fracture* | 0 | 2265 (86.2%) |
|  | 1 | 250 (9.5%) |
|  | 2 | 78 (3.0%) |
|  | 3 | 23 (0.9%) |
|  | 4 | 9 (0.3%) |
|  | 5 or more | 4 (0.2%) |
| *Closed fracture* | 0 | 737 (28.0%) |
|  | 1 | 439 (16.7%) |
|  | 2 | 367 (14.0%) |
|  | 3 | 310 (11.8%) |
|  | 4 | 233 (8.9%) |
|  | 5 or more | 543 (20.7%) |
| *Organ traumatic lesion with internal wound* | 0 | 2357 (89.7%) |
|  | 1 | 204 (7.8%) |
|  | 2 | 51 (1.9%) |
|  | 3 | 12 (0.5%) |
|  | 4 | 5 (0.2%) |
| *Organ traumatic lesion without internal wound* | 0 | 960 (36.5%) |
|  | 1 | 584 (22.2%) |
|  | 2 | 471 (17.9%) |
|  | 3 | 302 (11.5%) |
|  | 4 | 164 (6.2%) |
|  | 5 or more | 148 (5.6%) |
| *Organ traumatic lesion without details on internal wound* | 0 | 1358 (51.7%) |
|  | 1 | 546 (20.8%) |
|  | 2 | 338 (12.9%) |
|  | 3 | 190 (7.2%) |
|  | 4 | 110 (4.2%) |
|  | 5 or more | 87 (3.3%) |
| *Traumatic lesion of limbs, nerves, vessels, muscles or tendons* | 0 | 1374 (52.3%) |
|  | 1 | 808 (30.7%) |
|  | 2 | 309 (11.8%) |
|  | 3 | 99 (3.8%) |
|  | 4 | 28 (1.1%) |
|  | 5 or more | 11 (0.4%) |
| *Open wound* | 0 | 1642 (62.5%) |
|  | 1 | 741 (28.2%) |
|  | 2 | 165 (6.3%) |
|  | 3 | 54 (2.1%) |
|  | 4 | 17 (0.7%) |
|  | 5 or more | 10 (0.4%) |
| *Internal bleeding* | 0 | 1154 (43.9%) |
|  | 1 | 748 (28. 5%) |
|  | 2 | 427 (16.2%) |
|  | 3 | 197 (7.5%) |
|  | 4 | 68 (2.6%) |
|  | 5 or more | 35 (1.3%) |
| *Amputation* | 0 | 2588 (98.4%) |
|  | 1 | 25 (1.0%) |
|  | 2 | 11 (0.4%) |
|  | 3 | 4 (0.2%) |
|  | 5 or more | 1 (0.0%) |
| *Laceration* | 0 | 2627 (99.9%) |
|  | 1 | 2 (0.1%) |
| *Organ crushing* | 0 | 2509 (95.4%) |
|  | 1 | 96 (3.7%) |
|  | 2 | 17 (0.7%) |
|  | 3 | 7 (0.3%) |
| *Superficial wound* | 0 | 2610 (99.3%) |
|  | 1 | 19 (0.7%) |
| *Luxation* | 0 | 2445 (93.0%) |
|  | 1 | 171 (6.5%) |
|  | 2 | 12 (0.5%) |
|  | 3 | 1 (0.0%) |
| *Contusion* | 0 | 2557 (97.3%) |
|  | 1 | 69 (2.6%) |
|  | 2 | 2 (0.1%) |
|  | 5 or more | 1 (0.0%) |
| **Severe traumatic lesions according to ICD-10 coding, head trauma** | | |
| *Traumatic lesion* | 0 | 1477 (56.2%) |
|  | 1 | 380 (14.5%) |
|  | 2 | 257 (9.8%) |
|  | 3 | 189 (7.2%) |
|  | 4 | 140 (5.3%) |
|  | 5 or more | 186 (7.1%) |
| *Open fracture* | 0 | 2620 (99.7%) |
|  | 1 | 9 (0.3%) |
| *Closed fracture* | 0 | 2218 (84.4%) |
|  | 1 | 407 (15.5%) |
|  | 2 | 4 (0.2%) |
| *Organ traumatic lesion with internal wound* | 0 | 2608 (99.2%) |
|  | 1 | 19 (0.7%) |
|  | 2 | 2 (0.1%) |
| *Organ traumatic lesion without internal wound* | 0 | 1894 (72.0%) |
|  | 1 | 466 (17.7%) |
|  | 2 | 200 (7.6%) |
|  | 3 | 62 (2.4%) |
|  | 4 | 7 (0.3%) |
| *Organ traumatic lesion without details on internal wound* | 0 | 2025 (77.0%) |
|  | 1 | 318 (12.1%) |
|  | 2 | 164 (6.2%) |
|  | 3 | 93 (3.5%) |
|  | 4 | 22 (0.8%) |
|  | 5 or more | 7 (0.3%) |
| *Traumatic lesion of limbs, nerves, vessels, muscles or tendons* | 0 | 2165 (82.4%) |
|  | 1 | 415 (15.8%) |
|  | 2 | 47 (1.8%) |
|  | 3 | 2 (0.1%) |
| *Open wound* | 0 | 2611 (99.3%) |
|  | 1 | 18 (0.7%) |
| *Internal bleeding* | 0 | 2101 (79.9%) |
|  | 1 | 368 (14.0%) |
|  | 2 | 125 (4.8%) |
|  | 3 | 22 (0.8%) |
|  | 4 | 13 (0.5%) |
| **Severe traumatic lesions according to ICD-10 coding, chest trauma** | | |
| *Traumatic lesion* | 0 | 1182 (45.0%) |
|  | 1 | 572 (21.8%) |
|  | 2 | 357 (13.6%) |
|  | 3 | 242 (9.2%) |
|  | 4 | 136 (5.2%) |
|  | 5 or more | 140 (5.3%) |
| *Open fracture* | 0 | 2622 (99.7%) |
|  | 1 | 7 (0.3%) |
| *Closed fracture* | 0 | 2473 (94.1%) |
|  | 1 | 156 (5.9%) |
| *Organ traumatic lesion with internal wound* | 0 | 2512 (95.6%) |
|  | 1 | 90 (3.4%) |
|  | 2 | 22 (0.8%) |
|  | 3 | 4 (0.2%) |
|  | 4 | 1 (0.0%) |
| *Organ traumatic lesion without internal wound* | 0 | 1515 (57.6%) |
|  | 1 | 608 (23.1%) |
|  | 2 | 324 (12.3%) |
|  | 3 | 108 (4.1%) |
|  | 4 | 54 (2.1%) |
|  | 5 or more | 20 (0.8%) |
| *Organ traumatic lesion without details on internal wound* | 0 | 1981 (75.4%) |
|  | 1 | 419 (15.9%) |
|  | 2 | 163 (6.2%) |
|  | 3 | 52 (2.0%) |
|  | 4 | 11 (0.4%) |
|  | 5 or more | 3 (0.1%) |
| *Traumatic lesion of limbs, nerves, vessels, muscles or tendons* | 0 | 2469 (93.9%) |
|  | 1 | 135 (5.1%) |
|  | 2 | 24 (0.9%) |
|  | 3 | 1 (0.0%) |
| *Organ crushing* | 0 | 2626 (99.9%) |
|  | 1 | 3 (0.1%) |
| *Internal bleeding* | 0 | 2097 (79.8%) |
|  | 1 | 292 (11.1%) |
|  | 2 | 198 (7.5%) |
|  | 3 | 37 (1.4%) |
|  | 4 | 5 (0.2%) |
| **Severe traumatic lesions according to ICD-10 coding, abdominal trauma** | | |
| *Traumatic lesion* | 0 | 2020 (76.8%) |
|  | 1 | 341 (13.0 %) |
|  | 2 | 174 (6.6%) |
|  | 3 | 53 (2.0%) |
|  | 4 | 24 (0.9%) |
|  | 5 or more | 17 (0.7%) |
| *Organ traumatic lesion with internal wound* | 0 | 2521 (95.9%) |
|  | 1 | 87 (3.3%) |
|  | 2 | 13 (0.5%) |
|  | 3 | 6 (0.2%) |
|  | 4 | 2 (0.1%) |
| *Organ traumatic lesion without internal wound* | 0 | 2408 (91.6%) |
|  | 1 | 209 (8.0%) |
|  | 2 | 10 (0.4%) |
|  | 3 | 1 (0.0%) |
|  | 4 | 1 (0.0%) |
| *Organ traumatic lesion without details on internal wound* | 0 | 2490 (94.7%) |
|  | 1 | 125 (4.8%) |
|  | 2 | 12 (0.5%) |
|  | 3 | 1 (0.0%) |
|  | 4 | 1 (0.0%) |
| *Traumatic lesion of limbs, nerves, vessels, muscles or tendons* | 0 | 2248 (85.5%) |
|  | 1 | 273 (10.4%) |
|  | 2 | 94 (3.6%) |
|  | 3 | 13 (0.5%) |
|  | 4 | 1 (0.0%) |
| **Severe traumatic lesions according to ICD-10 coding, pelvic and spinal trauma** | | |
| *Traumatic lesion* | 0 | 1949 (74.1%) |
|  | 1 | 364 (13.9%) |
|  | 2 | 191 (7.3%) |
|  | 3 | 89 (3.4%) |
|  | 4 | 32 (1.2%) |
|  | 5 or more | 4 (0.2%) |
| *Open fracture* | 0 | 2616 (99.5%) |
|  | 1 | 10 (0.4%) |
|  | 2 | 2 (0.1%) |
|  | 3 | 1 (0.0%) |
| *Closed fracture* | 0 | 2099 (79.8%) |
|  | 1 | 260 (9. 9%) |
|  | 2 | 163 (6.2%) |
|  | 3 | 83 (3.2%) |
|  | 4 | 24 (0.9%) |
| *Organ crushing* | 0 | 2628 (100.0%) |
|  | 1 | 1 (0.0%) |
| *Traumatic lesion of limbs, nerves, vessels, muscles or tendons* | 0 | 2462 (93.7%) |
|  | 1 | 159 (6.1%) |
|  | 2 | 6 (0.2%) |
|  | 3 | 2 (0.1%) |
| **Severe traumatic lesions according to ICD-10 coding, kidney trauma** | | |
| *Traumatic lesion* | 0 | 2454 (93.3%) |
|  | 1 | 141 (5.4%) |
|  | 2 | 33 (1.3%) |
|  | 3 | 1 (0.0%) |
| *Organ traumatic lesion with internal wound* | 0 | 2620 (99.7%) |
|  | 1 | 9 (0.34%) |
| *Organ traumatic lesion without internal wound* | 0 | 2483 (94.5%) |
|  | 1 | 140 (5.3%) |
|  | 2 | 6 (0.2%) |
| *Organ traumatic lesion without details on internal wound* | 0 | 2580 (98.1%) |
|  | 1 | 49 (1.9%) |
| **Severe traumatic lesions according to ICD-10 coding, urinary system and genital organs (excepted for kidney)** | | |
| *Traumatic lesion* | 0 | 2574 (97.9%) |
|  | 1 | 43 (1.6%) |
|  | 2 | 8 (0.3%) |
|  | 3 | 4 (0.2%) |
| *Organ traumatic lesion with internal wound* | 0 | 2617 (99.5%) |
|  | 1 | 11 (0.4%) |
|  | 2 | 1 (0.0%) |
| *Organ traumatic lesion without internal wound* | 0 | 2593 (98.6%) |
|  | 1 | 34 (1.3%) |
|  | 2 | 2 (0.1%) |
| *Organ traumatic lesion without details on internal wound* | 0 | 2609 (99.2%) |
|  | 1 | 20 (0.8%) |
| **Severe traumatic lesions according to ICD-10 coding, upper limb trauma** | | |
| *Traumatic lesion* | 0 | 2500 (95.1%) |
|  | 1 | 98 (3.7%) |
|  | 2 | 21 (0.8%) |
|  | 3 | 6 (0.2%) |
|  | 4 | 2 (0.1%) |
|  | 5 or more | 2 (0.1%) |
| *Open fracture* | 0 | 2560 (97.4%) |
|  | 1 | 55 (2.1%) |
|  | 2 | 10 (0.4%) |
|  | 3 | 2 (0.1%) |
|  | 4 | 1 (0.0%) |
|  | 5 or more | 1 (0.0%) |
| *Open fracture* | 0 | 2624 (99.8%) |
|  | 1 | 3 (0.1%) |
|  | 2 | 1 (0.0%) |
|  | 3 | 1 (0.0%) |
| *Organ crushing* | 0 | 2617 (99.5%) |
|  | 1 | 11 (0.4%) |
|  | 2 | 1 (0.0%) |
| *Traumatic lesion of limbs, nerves, vessels, muscles or tendons* | 0 | 2578 (98.1%) |
|  | 1 | 40 (1.5%) |
|  | 2 | 7 (0.3%) |
|  | 3 | 3 (0.1%) |
|  | 4 | 1 (0.0%) |
| **Severe traumatic lesions according to ICD-10 coding, lower limb trauma** | | |
| *Traumatic lesion* | 0 | 2186 (83.2%) |
|  | 1 | 233 (8.9%) |
|  | 2 | 143 (5.4%) |
|  | 3 | 43 (1.6%) |
|  | 4 | 13 (0.5%) |
|  | 5 or more | 11 (0.4%) |
| *Open fracture* | 0 | 2509 (95.4%) |
|  | 1 | 102 (3.9%) |
|  | 2 | 13 (0.5%) |
|  | 3 | 5 (0.2%) |
| *Closed fracture* | 0 | 2314 (88.0%) |
|  | 1 | 203 (7.7%) |
|  | 2 | 96 (3.7%) |
|  | 3 | 16 (0.6%) |
| *Open fracture* | 0 | 2604 (99.1%) |
|  | 1 | 19 (0.7%) |
|  | 2 | 5 (0.2%) |
|  | 3 | 1 (0.0%) |
| *Organ crushing* | 0 | 2582 (98.2%) |
|  | 1 | 40 (1.5%) |
|  | 2 | 6 (0.2%) |
|  | 3 | 1 (0.0%) |
| *Traumatic lesion of limbs, nerves, vessels, muscles or tendons* | 0 | 2562 (97.5%) |
|  | 1 | 49 (1.9%) |
|  | 2 | 16 (0.6%) |
|  | 3 | 2 (0.1%) |
| **Traumatic lesions according to ICD-10 coding and AIS topography** | | |
| *External* | 0 | 2594 (98.7%) |
|  | 1 | 32 (1.2%) |
|  | 2 | 1 (0.0%) |
|  | 3 | 2 (0.1%) |
| *Head and neck* | 0 | 1001 (38.1%) |
|  | 1 | 439 (16.7%) |
|  | 2 | 285 (10.8%) |
|  | 3 | 255 (9.7%) |
|  | 4 | 187 (7.11%) |
|  | 5 or more | 462 (17.6%) |
| *Face* | 0 | 1692 (64.4%) |
|  | 1 | 508 (19.3%) |
|  | 2 | 189 (7.2%) |
|  | 3 | 116 (4.4%) |
|  | 4 | 71 (2.7%) |
|  | 5 or more | 53 (2.0%) |
| *Thorax* | 0 | 988 (37.6%) |
|  | 1 | 465 (17.7%) |
|  | 2 | 357 (13.6%) |
|  | 3 | 297 (11.3%) |
|  | 4 | 199 (7.6%) |
|  | 5 or more | 323 (12.3%) |
| *Abdomen* | 0 | 1408 (53.6%) |
|  | 1 | 363 (13.8%) |
|  | 2 | 265 (10.1%) |
|  | 3 | 200 (7.6%) |
|  | 4 | 143 (5.4%) |
|  | 5 or more | 250 (9.5%) |
| *Extremities or pelvic girdle* | 0 | 1400 (53.3%) |
|  | 1 | 465 (17.7%) |
|  | 2 | 301 (11.5%) |
|  | 3 | 180 (6.9%) |
|  | 4 | 104 (4.0%) |
|  | 5 or more | 179 (6.8%) |
| **Overall traumatic lesions according to ICD-10 coding and established classification of severity of lesions** | | |
| *GHM26* | No | 1341 (51.0%) |
|  | Yes | 1288 (49.0%) |
| *Number of diagnostic codes of severe trauma* | 0 | 512 (19.5%) |
|  | 1 | 912 (34.7%) |
|  | 2 | 461 (17.5%) |
|  | 3 | 267 (10.2%) |
|  | 4 | 207 (7.9%) |
|  | 5 or more | 270 (10.3%) |
| *Number of diagnostic codes of moderate trauma* | 0 | 272 (10.4%) |
|  | 1 | 405 (15.4%) |
|  | 2 | 615 (23.4%) |
|  | 3 | 465 (17.7%) |
|  | 4 | 298 (11.3%) |
|  | 5 or more | 574 (21.8%) |
| *Number of diagnostic codes of non-severe trauma* | 0 | 491 (18.7%) |
|  | 1 | 554 (21.1%) |
|  | 2 | 437 (16.6%) |
|  | 3 | 341 (13.0%) |
|  | 4 | 296 (11.3%) |
|  | 5 or more | 510 (19.4%) |
| **Traumatic lesions according to ICD-10 coding and AIS topography by severity according to established classification** | | |
| *Head and neck, severe lesion* | 0 | 1493 (56.8%) |
|  | 1 | 413 (15.7%) |
|  | 2 | 258 (9.8%) |
|  | 3 | 194 (7.4%) |
|  | 4 | 140 (5.3%) |
|  | 5 or more | 131 (5.0%) |
| *Head and neck,moderately severe lesion* | 0 | 1382 (52.6%) |
|  | 1 | 653 (24.8%) |
|  | 2 | 335 (12.7%) |
|  | 3 | 155 (5.9%) |
|  | 4 | 76 (2.9%) |
|  | 5 or more | 28 (1.1%) |
| *Head and neck, mild lesion* | 0 | 2219 (84.4%) |
|  | 1 | 331 (12.6%) |
|  | 2 | 60 (2.3%) |
|  | 3 | 14 (0.5%) |
|  | 4 | 4 (0.2%) |
|  | 5 or more | 1 (0.0%) |
| *Face, severe lesion* | 0 | 2600 (98.9%) |
|  | 1 | 27 (1.0%) |
|  | 2 | 2 (0.1%) |
| *Face, moderately severe lesion* | 0 | 2020 (76.8%) |
|  | 1 | 554 (21.1%) |
|  | 2 | 51 (1.9%) |
|  | 3 | 3 (0.1%) |
|  | 4 | 1 (0.0%) |
| *Face, mild lesion* | 0 | 2005 (76.3%) |
|  | 1 | 330 (12.6%) |
|  | 2 | 170 (6.5%) |
|  | 3 | 69 (2.6%) |
|  | 4 | 34 (1.3%) |
|  | 5 or more | 21 (0.8%) |
| *Thorax, severe lesion* | 0 | 1952 (74.3%) |
|  | 1 | 520 (19. 8%) |
|  | 2 | 131 (5.0%) |
|  | 4 | 4 (0.2%) |
|  | 5 or more | 1 (0.0%) |
| *Thorax, moderately severe lesion* | 0 | 1409 (53.6%) |
|  | 1 | 622 (23.7%) |
|  | 2 | 306 (11.6%) |
|  | 3 | 179 (6.8%) |
|  | 4 | 68 (2.6%) |
|  | 5 or more | 45 (1.7%) |
| *Thorax, mild lesion* | 0 | 1425 (54.2%) |
|  | 1 | 792 (30.1%) |
|  | 2 | 327 (12.4%) |
|  | 3 | 78 (3.0%) |
|  | 4 | 7 (0.3%) |
| *Abdomen, severe lesion* | 0 | 2117 (80.5%) |
|  | 1 | 403 (15.3%) |
|  | 2 | 93 (3.5%) |
|  | 3 | 15 (0.6%) |
|  | 5 or more | 1 (0.0%) |
| *Abdomen, moderately severe lesion* | 0 | 1613 (61.4%) |
|  | 1 | 391 (14.9%) |
|  | 2 | 275 (10.5%) |
|  | 3 | 174 (6.6%) |
|  | 4 | 93 (3.5%) |
|  | 5 or more | 83 (3.2%) |
| *Abdomen, mild lesion* | 0 | 2060 (78.4%) |
|  | 1 | 473 (18.0%) |
|  | 2 | 88 (3.4%) |
|  | 3 | 8 (0.3%) |
| *Extremities or pelvic girdle, severe lesion* | 0 | 2564 (97.5%) |
|  | 1 | 55 (2.1%) |
|  | 2 | 7 (0.3%) |
|  | 3 | 2 (0.1%) |
|  | 4 | 1 (0.0%) |
| *Extremities or pelvic girdle, moderately severe lesion* | 0 | 2358 (89.7%) |
|  | 1 | 210 (8.0%) |
|  | 2 | 44 (1.7%) |
|  | 3 | 12 (0.5%) |
|  | 4 | 3 (0.1%) |
|  | 5 or more | 2 (0.1%) |
| *Extremities or pelvic girdle, mild lesion* | 0 | 1445 (55.0%) |
|  | 1 | 506 (19.3%) |
|  | 2 | 278 (10.6%) |
|  | 3 | 180 (6.9%) |
|  | 4 | 93 (3.5%) |
|  | 5 or more | 127 (4.8%) |

For each label, the number of code is reported. Values are reported as numbers and relative percentages. After admission to intensive care unit, trauma patients who neither die nor are discharged directly home are referred to either a surgical unit or a medical ward.

## **Supplementary Material 5**: Relevant diagnoses for TRISS calculation

| **Variable** | | **Value** |
| --- | --- | --- |
| *Shock requiring fluid resuscitation or catecholamines (admission)* | No | 1899 (72.2%) |
|  | Yes | 730 (27.8%) |
| *Coma* | No | 2310 (87.9%) |
|  | Yes | 319 (12.1%) |
| *SAPS II (admission)* | Min / Max | 0 / 128 |
|  | Med [IQR] | 30 [17 ; 48] |
|  | Moy (std) | 34.81 (22.5) |
|  | N (MV) | 2594 (35) |

The data considered for this analysis are those from the coding of the first service. IQR, Inter Quartile Range; MV, Missing value; std, standard deviation, SAPS II, Simplified Acute Physiology Score 2.

## **Supplementary Material 6**: Correlation between identical variables from the medical record and from the hospital coding**.** Spearman's correlation coefficient for SAPS II: 0.88

### S6.A: SAPS II

### S6.B: Shock

| **Shock (Hospital coding)** | | |
| --- | --- | --- |
| Shock (Données hospitalières) | No | Yes |
| No | 63.2 | 13.1 |
| Yes | 12.9 | 10.8 |

Consistency of Shock diagnoses, from the medical record and the hospital. Results are expressed as percentages of the overall cohort without missing data (n = 2071). Cohen's kappa coefficient: 0.28 CI95[0.25,0.32]. Shock codes were validated against systolic and diastolic blood pressure values recorded at admission in the Traumabase. Shock was defined as systolic blood pressure <90 mmHg or diastolic blood pressure <60 mmHg.

### S6.C: Coma

| **Coma (Hospital coding)** | | |
| --- | --- | --- |
| Coma (medical record) | No | Yes |
| No | 58.7 | 3.8 |
| Yes | 23.3 | 14.2 |

Consistency of Coma diagnoses, from the medical record and the hospital coding. Results are expressed as percentages of the overall cohort without missing data (n = 261). Cohen's kappa coefficient: 0.36 CI95[0.32,0.39]. Coma codes were validated against the Glasgow Coma Scale (GCS) recorded at admission. Patients were classified as comatose if the GCS was <9 or if they were sedated

## **Supplementary Material 7:** **Cross-description of AIS from the medical record and Hospital coding, based on GHM26 trauma severity codes**. Here we present the cumulative counts of unique codes from list D130 over the entire hospitalization, classified according to the AIS anatomical regions and stratified by the AIS severity score of the corresponding region. An alternative approach would have been to examine the “severe” codes listed in GHM26 (lists D131 to D138). However, no codes for facial or external injuries are included in these lists. The line represents the linear regression of AIS as a function of the number of diagnoses. The confidence intervals were estimated by bootstrap with 9,999 iterations. ρ, Spearman’s rank correlation. S7.A: Correlation of number of unique diagnoses of head and neck trauma according to hospital coding and head and neck AIS. S7.B: Correlation of number of unique diagnoses of face trauma according to hospital coding and face AIS. S7.C: Correlation of number of unique diagnoses of chest trauma according to hospital coding and chest AIS. S7.D: Correlation of number of unique diagnoses of abdominal trauma according to hospital coding and abdomen AIS. S7.E: Correlation of number of unique diagnoses of extremities trauma according to hospital coding and extremities AIS. S7.F: Correlation of number of unique diagnoses of extremities trauma according to hospital coding and External AIS.

## **Supplementary Material 8: Predictive models with aggregated covariates for hospital coding**. The covariates 'Shock', Coma' and 'Penetrating lesion', mean that at least one hospital code relative to these three diagnoses is present during the whole hospital stay. The covariates 'Head and neck', 'Face', 'Chest', 'Abdomen', 'Extremities' are the cumulative numbers of diagnoses of traumatic lesions in these anatomical area during the whole hospitalization stay. The SAPSII is the value entered by the first hospitalization unit.

### S8.A: Coefficients from the linear and ridge regression

|  | **Linear regression** | **Ridge regression** |
| --- | --- | --- |
| **Variables** | **Coefficient** | |
| Intercept | -2.73 | -2.65 |
| Age | 0.15 | 0.18 |
| Shock | 0.16 | 0.14 |
| Coma | 0.25 | 0.13 |
| Penetrating lesion | -0.38 | -0.06 |
| Head and neck | 0.43 | 0.42 |
| Face | -0.15 | -0.13 |
| Chest | 0.23 | 0.22 |
| Abdomen | 0.12 | 0.12 |
| Extremities | -0.10 | -0.09 |
| SAPS II | 1.63 | 1.47 |
| Model performances measures | **Penalty term lambda** | |
|  | - | 0.18 |
|  | **Apparent R squared** | |
|  | 0.63 | 0.63 |
|  | **Optimism-adjusted R squared** | |
|  | 0.63 | 0.63 |

### S8.B: Extreme gradient boosting regression

## S8.B1: Percentage of explained variability by each covariate in the extreme gradient boosting model

This is a Machine Learning regression method based on random forests (classification by repeatedly applying decision trees). These methods are based on the idea that estimating the model multiple times and combining the results helps minimize prediction error better than estimating a single model. The model uses six parameters to control its fit to the data: 1/ Eta: Shrinkage parameter; this parameter controls how much information from one tree will be reused to estimate the next; 2/ Nrounds: The number of trees built to classify the individuals ; 3/ Subsample ratio of columns when constructing each tree: This is the proportion of variables randomly selected to choose each node in the decision tree ; 4/ Maximum depth: The maximum depth of the tree. The more variables used to classify individuals, the deeper the tree, but the less generalizable it will be to another population; 5/ Minimum sum of instance weight needed in a child: The minimum number of observations required in a leaf after a split by a node; and Subsample: The proportion of total observations used to build each tree.

The covariates 'Shock', Coma' and 'Penetrating lesion', mean that at least one hospital code relative to these three diagnoses is present during the whole hospital stay. The covariates 'Head and neck', 'Face', 'Chest', 'Abdomen', 'Extremities' are the cumulative numbers of diagnoses of traumatic lesions in these anatomical area during the whole hospitalization stay. The SAPSII is the value entered by the first hospitalization unit.

## S8.B2: Parameters of the extreme gradient boosting model

| Parameters | Value |
| --- | --- |
| Eta | 0.4 |
| Maximum depth | 3.0 |
| Subsample ratio of columns when constructing each tree | 0.6 |
| Minimum sum of instance weight needed in a child | 1.0 |
| Subsample | 1.0 |
| Number of rounds | 150.0 |

## Supplementary Material 9: Predictive models with individual codes as covariates

### S9.A: Coefficients from the Ridge regression

| Label | Code | Coefficient |
| --- | --- | --- |
| (Intercept) | - | -3.38 |
| SAPS II | - | 1.36 |
| Coma | R402 | 0.54 |
| Traumatic cerebral edema | S061 | 0.48 |
| Fracture of sternum | S222 | 0.47 |
| Traumatic brain injury with prolonged coma | S067 | 0.41 |
| Injury of heart, unspecified with or without hemopericardium | S269 | 0.40 |
| Diffuse traumatic brain injury | S062 | 0.36 |
| Fracture of third cervical vertebra | S122 | 0.31 |
| Multiple fractures of lumbar spine and pelvis | S327 | 0.30 |
| Traumatic pneumothorax | S270 | 0.30 |
| Fracture of base of skull | S021 | 0.26 |
| Traumatic subarachnoid hemorrhage | S066 | 0.25 |
| Injury of other intra-abdominal organs | S368 | -0.25 |
| Fracture of clavicle | S420 | -0.24 |
| Epidural hemorrhage | S064 | 0.24 |
| Injury of liver and gallbladder and bile duct | S361 | 0.24 |
| Age | - | 0.24 |
| Traumatic ischemia of muscle | T796 | -0.21 |
| Traumatic secondary and recurrent hemorrhage and seroma | T792 | 0.21 |
| Injury of kidney | S370 | 0.20 |
| Traumatic shock | T794 | 0.19 |
| Traumatic hemopneumothorax | S272 | 0.19 |
| Fracture of one rib | S223 | 0.17 |
| Fracture of orbital floor | S023 | -0.15 |
| Fracture of shaft of tibia | S822 | -0.14 |
| Traumatic subcutaneous emphysema | T797 | -0.14 |
| Flail chest | S225 | 0.14 |
| Fracture of acetabulum | S324 | 0.13 |
| Traumatic subdural hemorrhage | S065 | 0.11 |
| Fracture of thoracic vertebra | S220 | -0.11 |
| Focal traumatic brain injury | S063 | -0.11 |
| Other and unspecified injuries of lung | S273 | 0.11 |
| Traumatic hemothorax | S271 | 0.10 |
| Unspecified fracture of femur | S729 | 0.08 |
| Fracture of pubis | S325 | 0.07 |
| Fracture of sacrum | S321 | 0.06 |
| Fracture of ilium | S323 | 0.06 |
| Open wound of head | S017 | -0.06 |
| Fracture of scapula | S421 | 0.05 |
| Multiple fractures of ribs | S224 | 0.04 |
| Fracture of nasal bones | S022 | 0.03 |
| Unspecified intracranial injury | S069 | 0.03 |
| Hypovolemic shock | R571 | 0.03 |
| Fracture of malar, maxillary and zygoma bones | S024 | 0.03 |
| Other shock | R578 | 0.02 |
| Injury of spleen | S360 | -0.01 |
| Fracture of lumbar vertebra | S320 | -0.00 |

Penalty term lambda: 0.19;

Apparent R squared: 0.64;

Optimism-adjusted R squared: 0.63.

In this analysis, we examined the performance of models using individual codes as covariates. The list of codes corresponds to the codes from Table D-130 of GHM26, as well as the individual codes for coma, shock, and penetrating injuries (not aggregated into presence/absence categories or cumulative injury sums for a given area). Some codes were non-informative (no patients had the code) or very minimally informative (very few patients had the code), so they were removed from the regressions, leaving a total of 54 individual 4-digit codes introduced into the models (the fifth digit was excluded as models that included it showed highly correlated variables, and its external validity might be compromised due to varying coding practices). Here, only Ridge regression and extreme gradient boosting were applied, given the large number of covariates included in the models.

### S9.B: Extreme gradient boosting regression

## S9.B1: Percentage of explained variability by each covariate in the extreme gradient boosting model

## S9.B2: Parameters of the extreme gradient boosting model

| Variables | Value |
| --- | --- |
| Eta | 0.4 |
| Maximum depth | 3.0 |
| Subsample ratio of columns when constructing each tree | 0.6 |
| Minimum sum of instance weight needed in a child | 1.0 |
| Subsample | 1.0 |
| Number of rounds | 150.0 |
